# Insights into the role of dopamine in rhizosphere microbiome assembly

**DOI:** 10.1038/s41467-026-76211-1

**Published:** 2026-07-29

**Authors:** Yezhang Ding, Hunter K. Vogel, Yi Zhai, Hans K. Carlson, Peter F. Andeer, Vlastimil Novak, Nakian Kim, Benjamin P. Bowen, Amber N. Golini, Suzanne M. Kosina, Devin Coleman-Derr, John P. Vogel, Trent R. Northen

**Affiliations:** 1 Environmental Genomics and Systems Biology Division, Lawrence Berkeley National Laboratory, Berkeley, CA USA; 2 The DOE Joint Genome Institute, Lawrence Berkeley National Laboratory, Berkeley, CA USA; 3 Department of Plant and Microbial Biology, University of California, Berkeley, CA USA; 4 Plant Gene Expression Center, UC Berkeley, Albany, CA USA

**Keywords:** Secondary metabolism, Metabolomics, Plant genetics, Microbiome

## Abstract

Dopamine plays a critical role in animal physiology and interactions with gut microbes. In plants, dopamine is known to function in plant defense and abiotic stress tolerance; however, its role in mediating plant-microbiome interactions remains unexplored. In this study, we show that dopamine is one of the most abundant exometabolites with natural variation in root exudates across diverse *Brachypodium distachyon* lines, suggesting a potential role in rhizosphere microbial assembly. To further investigate this, we colonized ten natural *B. distachyon* lines with a 16-member bacterial synthetic community (SynCom), collected metabolomic and 16S rRNA sequencing data, and performed an association analysis. Our results reveal that dopamine levels in root exudates were significantly associated with the abundance of six SynCom members in a hydroponic system. In vitro growth studies demonstrated that dopamine had a significant effect on the growth of the same six bacterial isolates. Additionally, treating soil directly with dopamine enriched Actinobacteria, consistent with both the SynCom-dopamine correlations and the isolate growth results. Collectively, our study underscores the selective influence of dopamine on rhizosphere microbial communities, with implications for precision microbiome management.

## Abstract (web summary)

Dopamine is a neurotransmitter best known for its role in humans. Here the authors show that, in a model grass species, dopamine influences the composition of root-associated microbial communities.

## Introduction

In recent years, there has been a growing interest in the field of plant-microbiome interactions, primarily due to their significant potential to enhance agricultural production by fostering plant growth, plant defense, and soil health^1,2^. The rhizosphere is one of Earth’s most complex ecosystems, serving as a hotspot that fosters extraordinary microbial diversity. The composition of microbial communities in the rhizosphere varies with plant developmental stage, plant genotype, and soil environment^3–6^. Root exudates have been considered key drivers in mediating interactions of plants with rhizosphere microbes^7,8^.

Plant roots exude up to 20% of photosynthetically fixed carbon and 15% of the nitrogen absorbed by plants^8,9^. Root exudates include a diverse array of primary and specialized metabolites as well as complex polymers that shape crucial interactions within the rhizosphere^8,9^. The composition of root exudates is influenced by a combination of plant genetics, soil properties, microbial interactions, and environmental conditions^8,10^. Hydroponic systems are often used for studying root exudate composition and dynamics due to their controlled environment, direct access to exudates, non-destructive sampling methods, precision in experimental setup, and suitability for investigating specific plant-microbe interactions^11–14^. For example, the exudate dynamics of three phylogenetically distinct plant species, *Arabidopsis thaliana*, *Brachypodium distachyon*, and *Medicago truncatula*, were studied in hydroponics, revealing both significant differences between species and a core metabolome shared by all three species^14^.

Plant specialized metabolites are considered a potential mechanism shaping the plant-microbiome interactions due to their chemical diversity and antimicrobial properties^15,16^. Recent investigations have revealed that plant specialized metabolites, such as coumarin, camalexin, triterpenes, flavonoids, and benzoxazinoids, play a crucial role in mediating interactions between plants and the soil microbiome^16,17^. Pathway mutants are often used to discover the relationship between plant specialized metabolites and the root microbiome^17^. However, the use of genetic mutants relies on preexisting knowledge of the biosynthetic pathways and tends to be biased towards the study of readily identifiable candidate metabolites. In contrast, employing natural variation in natural lines allows for the relatively unbiased discovery of unexpected links between root exometabolites and the rhizosphere microbial community.

Catecholamines, such as dopamine and norepinephrine, are known to affect the human gut microbial growth^18–21^. These metabolites can be produced by commensal gut microorganisms and also derived from dietary sources^22^. Different human bacteria respond differently when exposed to these metabolites^20^. In addition, catecholamines can influence the expression of bacterial genes involved in virulence, stress response, and biofilm formation, thereby impacting the overall growth and function of the gut microbiota^23,24^. Many plant species also produce dopamine and its related compounds^25^. In plants, dopamine can promote plant disease resistance and enhance plant tolerance against abiotic stresses^26^. Recently, dopamine was shown to be present in the spent medium of the model grass *B. distachyon*^27^. However, its role in mediating plant-microbiome interactions has not been explored.

To uncover specialized metabolites involved in plant-microbiome interactions in *B. distachyon*, we inoculated diverse lines with a 16-member bacterial SynCom in a hydroponic system, collected metabolomic and 16S rRNA sequencing data, and conducted an association analysis. We found that the level of dopamine, one of the most abundant exometabolites, varied in root exudates of diverse *Brachypodium* lines. Correlation analysis with metabolomics and 16S rRNA sequencing data revealed that dopamine levels in root exudates were significantly associated with the abundance of two Actinobacteria and four Proteobacteria. Consistently, in vitro growth studies demonstrated that dopamine had a direct growth effect on the six SynCom bacteria. Furthermore, treating soil directly with dopamine specifically enriched Actinobacteria, supporting both the SynCom-dopamine correlations and the isolate growth results. Taken together, these findings suggest a mechanism through which plants use dopamine to modulate soil microbial communities.

## Results

### Natural variation in root exudates modulates a SynCom composition

To investigate the effects of natural variation in root exudates on plant-microbiome interactions, we selected ten *B. distachyon* lines that are the parental lines for a recombinant inbred line (RIL) population known to exhibit a large degree of phenotypic diversity^28^. These genetically and phenotypically diverse inbred *B. distachyon* lines were grown in a hydroponics-based system (for the workflow see Supplementary Fig. 1), with a 16-member bacterial SynCom isolated from a *Panicum virgatum* (switchgrass) rhizosphere (Supplementary Data 1)^29^. After an additional three weeks of incubation, we observed significant increases in the final OD_600_ of the plant spent medium as compared to that of the control (No Plants) (Supplementary Fig. 2). Consistently, we noted that these hydroponically grown *B. distachyon* lines displayed distinct growth phenotypes both in the absence and presence of the SynCom (Supplementary Fig. 3).

**Fig. 1:**
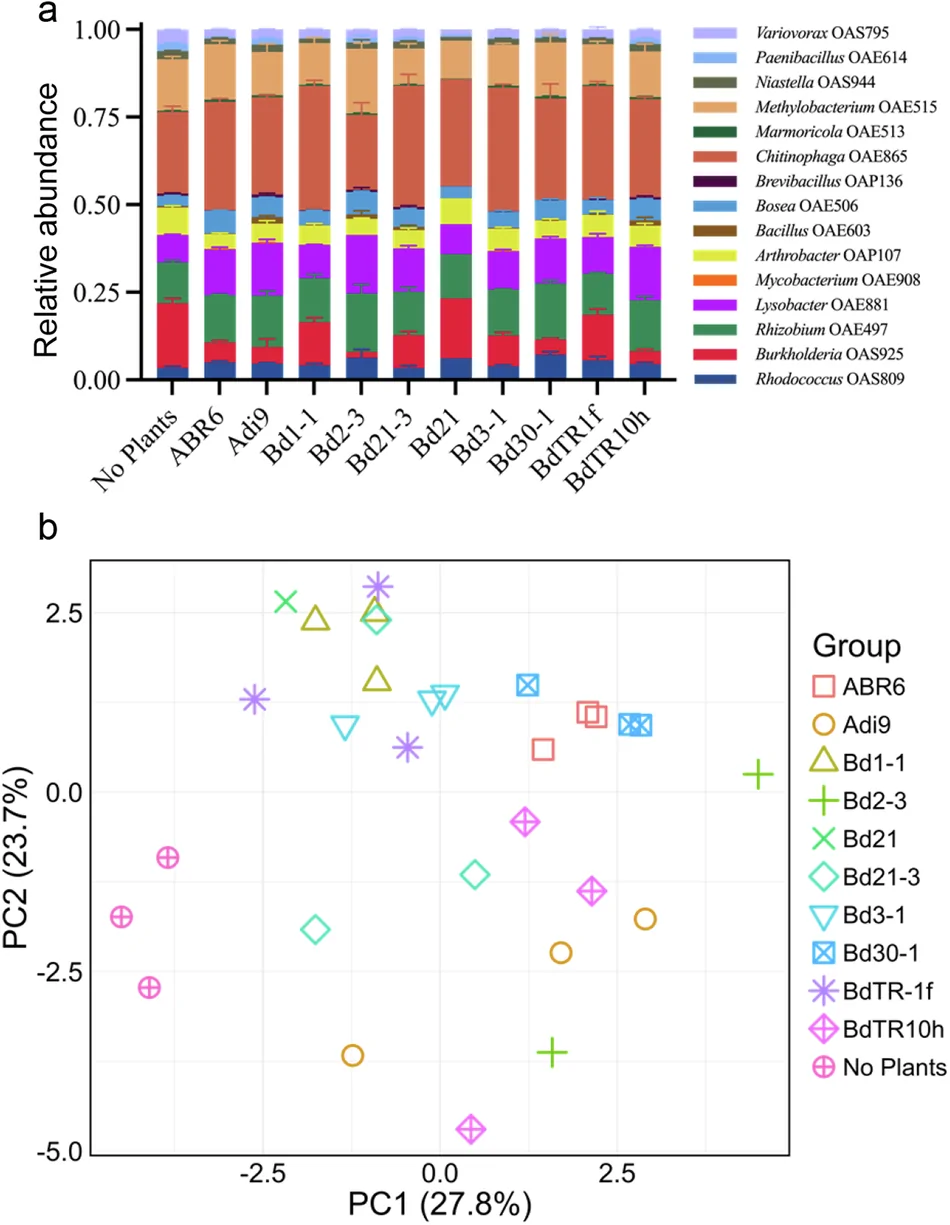
Distinct microbiome profiles across the ten *B. distachyon* lines. **a** Relative abundance of the SynCom members in the plant spent medium. Bacterial pellets were collected by centrifuging the plant medium for 16S rRNA sequencing. The SynCom inoculant was also added to the vessels filled with a plant-spent medium without plants (No Plants). Error bars represent mean ± SEM (*n* = 3 biologically independent samples except for Bd2-3 (*n* = 2) and Bd21 (*n* = 1)). **b** Principal coordinate analysis showing the difference in the SynCom composition in the plant spent medium of ten *Brachypodium* lines. The relative abundances of the SynCom members were used for the principal coordinate analysis.

We next examined how host genetic diversity shapes rhizosphere community composition. To do this, we performed 16S rRNA gene profiling on the rhizosphere communities, which revealed differential relative abundances of the SynCom members in the plant spent medium of certain *B. distachyon* lines (Fig. 1a and Supplementary Fig. 4; Supplementary Data 2). Notably, one bacterial strain, *Mucilaginibacter* OAE612, was not recovered by 16S rRNA gene amplicon sequencing, possibly due to unsuitable growth conditions for the bacterium. Principal coordinate analysis further revealed distinct microbiome profiles in the plant spent medium of some *B. distachyon* lines (Fig. 1b).

To explore chemical variation in root exudates of these *B. distachyon* lines, non-targeted metabolite profiling was conducted using liquid chromatography with tandem mass spectrometry (LC-MS/MS)^27^. In total, 3644 metabolic features were detected (Supplementary Data 3). The nonmetric multidimensional scaling ordination (NMDS) plot suggests distinct metabolite abundance levels in root exudates of certain *B. distachyon* lines (e.g., separation between ABR6, Bd3-1, and Adi9) in the absence of the SynCom (Supplementary Fig. 5). In comparison, the exudate metabolite profiles were less diverse in the presence of the SynCom (Supplementary Fig. 5), indicating that the SynCom presence buffered against differences in metabolite composition between *B. distachyon* lines that occurred in the absence of microbial partners.

To better understand the observed metabolic variation, we compared the similarities and differences in metabolic features detected in root exudates of these *B. distachyon* lines in the absence of the SynCom. Features detected in two or more lines account for 96% of total features while only 4% were found in all lines (Supplementary Fig. 6a). We also found that the features unique to an individual line accounted for 18% of all features; for each individual line, the number varied between 3 and 12% of all features (Supplementary Fig. 6b). Collectively, these results demonstrate that these *B. distachyon* lines have distinct metabolic profiles in their root exudates in the absence of the SynCom, providing evidence for the distinct microbiome profile observed in the plant spent medium.

Metabolic feature changes were also examined in the root exudates of individual lines in response to the SynCom treatment. The NMDS ordination plot revealed that the inoculated plants formed a separate group (Supplementary Fig. 5). We also observed that the patterns of metabolic signal changes following the SynCom treatment were significantly different across these ten *B. distachyon* lines (Supplementary Fig. 7). The proportion of increased features varied from 6 to 27% of all features, while decreased features ranged from 2 to 18% (Supplementary Fig. 7). Among these lines, BdTR1f, Bd21, and Bd1-1 had fewer features increased by the SynCom (6%, 6%, and 8%, respectively), whereas Bd30-1, BdTR1f, Bd2-3, and Bd21-3 had only 1%, 2%, 3%, and 5% of features decreased, respectively (Supplementary Fig. 7). Overall, we did not observe any increased or decreased features shared across all ten lines (Supplementary Fig. 8). Taken together, the metabolic changes in the root exudates in response to the SynCom varied across these *B. distachyon* genetic lines, providing further evidence for the observed distinct microbiome profile.

### Dopamine is a dominant exometabolite correlated with the SynCom composition and plant growth in a hydroponic system

**Fig. 2:**
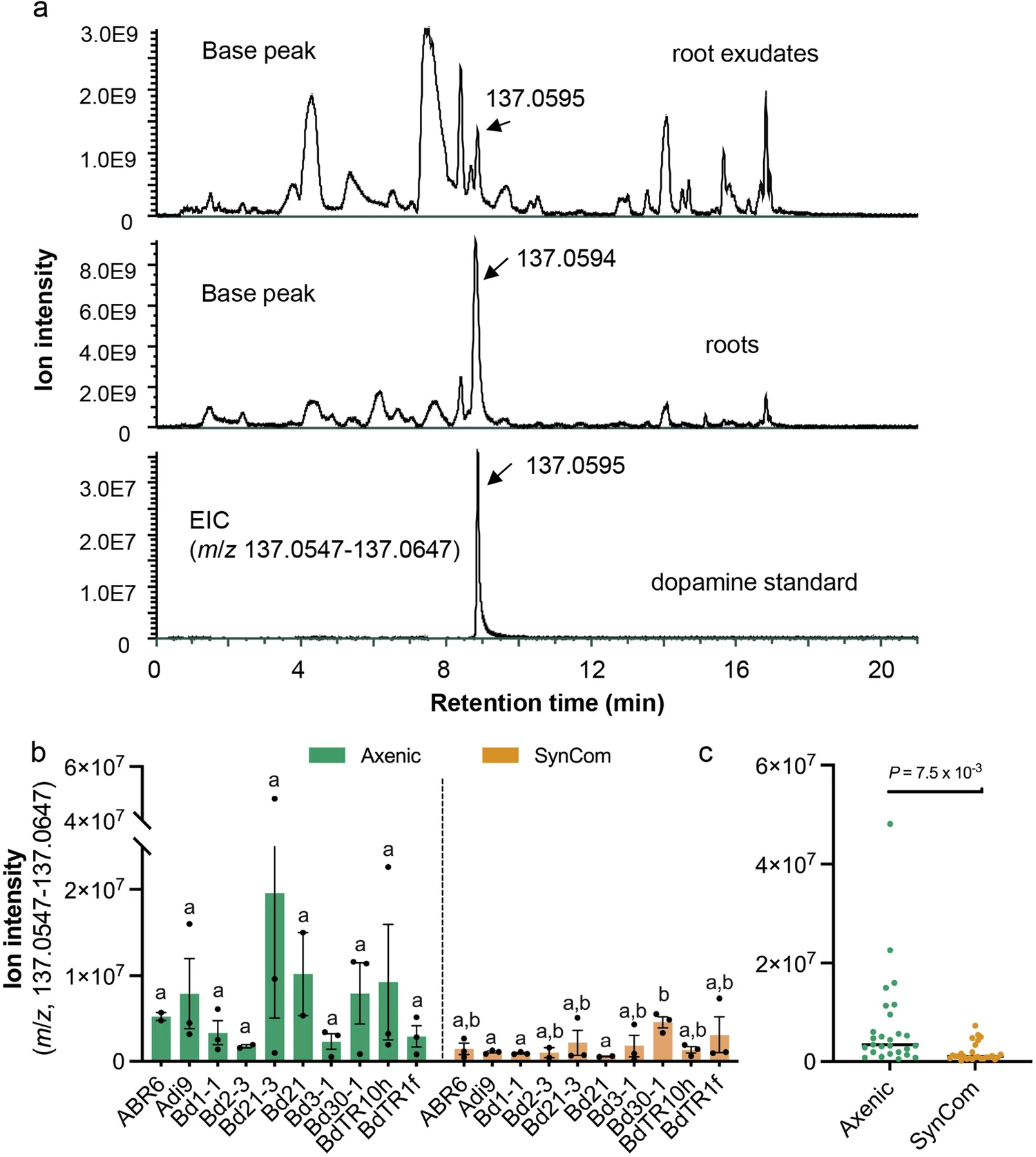
Dopamine is abundant in *B. distachyon* root exudates. **a** Representative LC-MS base peak chromatograms show dopamine as one of the most abundant metabolites in both *B. distachyon* exudates (concentrated) and roots, as well as extracted ion chromatogram (EIC) of the major dopamine ion [M + H - NH₃]⁺. *B. distachyon* Bd21-3 plants were grown in a hydroponic system. Plant spent media and the roots were collected from 4-week-old plants for LC-MS/MS analysis. **b** shows dopamine levels across ten *B. distachyon* lines in the absence and presence of a 16-member SynCom. Ten *B. distachyon* lines were grown in a hydroponic system in the absence and presence of a 16-member bacterial SynCom. Plant spent media were collected for LC-MS/MS analysis at week 4. Error bars represent the mean ± SEM of the peak height (*m*/*z* 137.0547-137.0647) (*n* = 3 biologically independent samples except for Bd2-3 and Bd21 (*n* = 2)). Statistical analyses were conducted separately for dopamine levels under axenic conditions and in the presence of the SynCom. Different letters (**a**, **b**) indicate significant differences as determined by two-sided Welch’s ANOVA followed by Dunnett’s T3 multiple comparison test (*P* < 0.05). **c** Shows a **c**ombined analysis (*n* = 28 biologically independent samples for the axenic group and *n* = 29 biologically independent samples for the SynCom group) in the absence and presence of the SynCom. Overall, the SynCom negatively affected the dopamine levels in root exudates. The *P-*value was calculated using a two-sided student’s *t* test.

Previously, we observed that dopamine was present in the spent medium of *B. distachyon*^27^. In this study, we found that the dopamine peak is one of the most intense peaks detected in both the *B. distachyon* roots and the root exudates (Fig. 2a and Supplementary Fig. 9). We also detected dopamine in the rhizosphere soil of *B. distachyon* Bd21-3 (Supplementary Fig. 10). In the initial experiment, dopamine levels in root exudates under axenic conditions showed substantial variability across the ten *B. distachyon* lines, and statistically significant differences between lines were not consistently observed (Fig. 2b and Supplementary Data 4). To further examine potential variation among lines, we performed an additional axenic experiment with increased biological replication (*n* = 6 per line), which revealed line-to-line variation in dopamine abundance in root exudates (Supplementary Fig. 11). In contrast, SynCom inoculation was consistently associated with reduced dopamine abundance across lines (Fig. 2b, 2c), suggesting potential microbial utilization of dopamine and/or altered dopamine production or exudation in response to microbial colonization. In humans, dopamine is known to play a role in regulating gut microbe growth^21^, suggesting a possible role for dopamine in regulating rhizosphere microbial assembly.

**Fig. 3:**
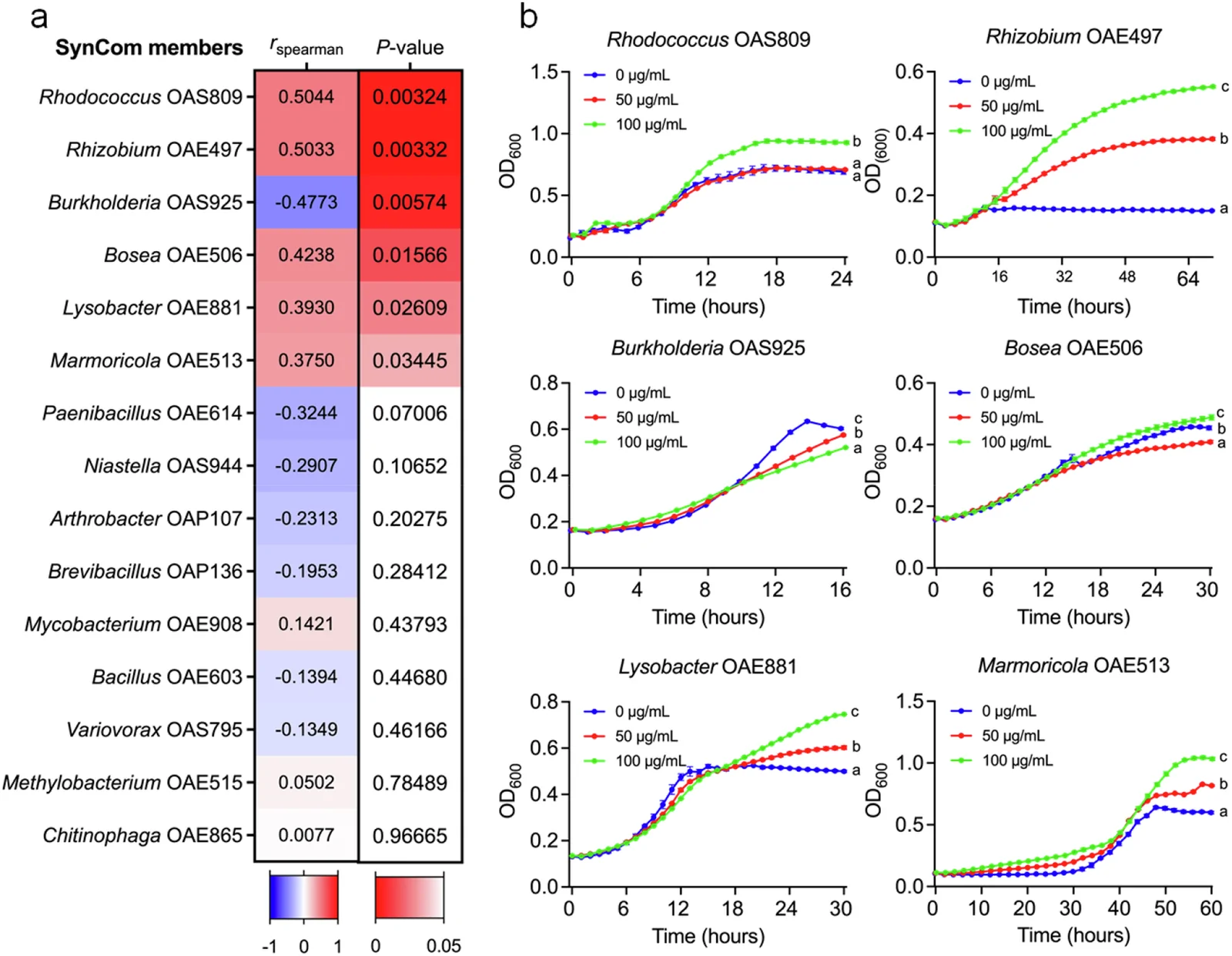
Dopamine regulates the SynCom composition in a hydroponic system. **a** Spearman correlation (*n* = 28) showing that dopamine levels were significantly associated with the relative abundance of six SynCom members either positively or negatively. Statistical significance was assessed using two-sided Spearman’s rank correlation tests. No multiple-comparison adjustment was applied. Correlations with *P* < 0.05 are highlighted in red. **b** In vitro **b**acterial growth assays showing that dopamine regulates microbial growth. Average (*n* = 4 biologically independent samples; ± SEM) bacterial growth estimates (OD_600_) of six SynCom members in liquid media (R2A or 1/10 R2A) in the presence of dopamine at 0 (blue), 50 (red), and 100 (green) µg/mL. Within plots, different letters represent significant differences for the final data point (one-way ANOVA, Tukey’s test corrections for multiple comparisons, *P* < 0.05).

To explore the possible function of dopamine in modulating plant-microbiome interactions, we performed an association analysis using the metabolomics and 16S rRNA amplicon sequencing data and found that dopamine levels in root exudates were significantly associated with the abundance of six SynCom members in the plant spent medium of the ten *B. distachyon* lines (Fig. 3a). Dopamine was positively associated with the relative abundances of two Actinobacteria, *Rhodococcus* OAS809 and *Marmoricola* OAE513, and three Proteobacteria, *Rhizobium* OAE497, *Lysobacter* OAE881, and *Bosea* OAE506, whereas it was negatively associated with another Proteobacteria, *Burkholderia* OAS925 (Fig. 3a).

To further investigate the mechanism underlying the potential role of dopamine in regulating the SynCom composition, we next performed in vitro assays to examine its growth effect on the six bacterial isolates whose abundances were significantly associated with the dopamine levels in the plant spent medium. To determine physiologically relevant concentrations for the assays, we measured dopamine concentration inside the roots since accurately estimating dopamine levels in root exudates is challenging. Based on an internal calibration curve of a [ring-^13^C_6_]-labeled dopamine (Supplementary Fig. 12a), we estimated that dopamine concentration could reach up to 2.71 ± 0.18 mg/g fresh weight in *B. distachyon* roots (Supplementary Fig. 12b). Next, we grew these six bacteria individually in liquid culture media supplemented with dopamine at two physiologically relevant concentrations and measured bacterial growth based on optical density at 600 nm (OD_600_) over time (Fig. 3b). Consistent with our association analysis, we observed that dopamine at 100 μg/mL significantly stimulated the growth of all five isolates whose abundances were positively associated with dopamine while inhibiting the growth of *Burkholderia* OAS925, whose abundance was negatively correlated with dopamine (Fig. 3b). These results demonstrate that dopamine may play an important role in modulating microbial abundance within a rhizosphere community context, likely by impacting microbe growth. Together with our observation that dopamine may be utilized by members of the microbial community (Fig. 2c), our findings suggest that dopamine may function both as a microbial growth substrate and as a potential modulator of microbial communities through other mechanisms.

Interestingly, we also observed that dopamine levels in exudates were positively associated with plant morphological phenotypes in the hydroponic system, including root biomass, shoot biomass, root length, and total biomass (Supplementary Fig. 13a). Plant growth assays confirmed that dopamine was able to promote the growth of hydroponically grown *B. distachyon* plants at the two lower levels used in our experiment (Supplementary Fig. 13b). Our result is consistent with previous studies showing that dopamine can promote plant growth under various stress conditions^26^.

### Exogenous application of dopamine alters soil microbiota

**Fig. 4:**
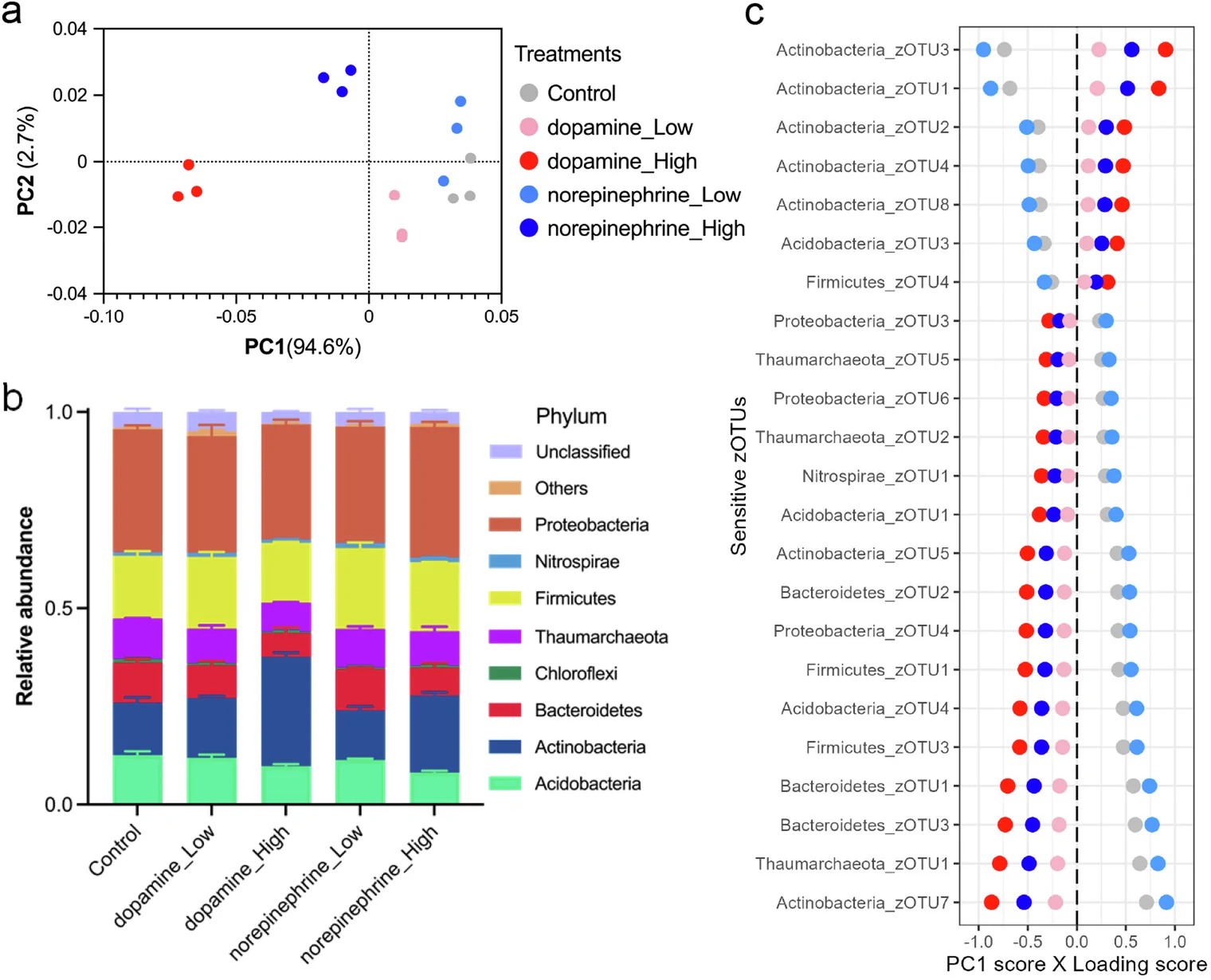
Dopamine modulates the soil microbiome. **a** The Principal coordinate analysis plot showing differences in bacterial community structures between samples. The color code depicts the different treatments (*n* = 3 biologically independent samples). **b** Relative abundance of bacterial phyla in the soil of the control or treatment with plant metabolites, dopamine and norepinephrine. Low abundant phyla with <1% of the total reads in all samples are summarized as “Others”. Error bars represent mean ± SEM (*n* = 3 biologically independent samples). **c** General trend of soil microbial response to dopamine and norepinephrine treatments. The relative responses of sensitive zOTUs to treatment are represented as their loading scores to principal component 1 (PC1) multiplied by the least-squares means (lsmeans) of PC1 by treatment level. Analysis was done on zOTUs with relative abundance >0.1% (*n* = 3 biologically independent samples) and those with loadings score to PC1 > 0.1 are displayed.

Given that dopamine is correlated with the SynCom composition and regulates soil bacterial growth in vitro, we hypothesized that dopamine likely impacts soil microbial communities in a native context. Since the genes involved in dopamine biosynthesis remain unknown in plants, we elected to test this by adding dopamine in dry form into 20 g of agricultural soil at two physiologically relevant concentrations (1.5 mg and 3.0 mg per 20 g of dry soil) and maintaining the soil with a water content of approximately 24.5%. We also included norepinephrine as a control for carbon (C) and nitrogen (N) source input into the soil since norepinephrine is a direct derivative of dopamine and has the same C/N ratio; however, norepinephrine was only found at a trace level in *B. distachyon* roots and was not detected in root exudates under our experimental conditions (Supplementary Fig. 14). The treatment was repeated twice a week over a period of 6 weeks. Based on sequencing analysis of the V4/V5 region of the 16S rRNA gene, zero-radius operational taxonomic units (zOTUs) were used to analyze soil bacterial diversity and membership across the soil treatments (Supplementary Data 5). Principal coordinate analysis revealed that both the chemical types and their concentrations were drivers in shaping the soil microbiome (Fig. 4a). The soil samples treated with the high dose of dopamine and norepinephrine formed two separate groups (Fig. 4a and Supplementary Fig. 15), suggesting that dopamine and norepinephrine at the high dose significantly, but differently altered the soil microbiota (Supplementary Fig. 15).

Taxonomic analysis revealed that in the control soil samples, six phyla, Proteobacteria, Firmicutes, Actinobacteria, Acidobacteria, Thaumarchaeota, and Bacteroidetes, were the most abundant, with average relative abundances of 31.3%, 16.0%, 13.5%, 12.5%, 10.2%, and 10.2%, respectively, while others were less abundant (<2%) (Fig. 4b and Supplementary Fig. 16). Among all bacterial phyla, only Actinobacteria were significantly enriched in soils treated with both dopamine and norepinephrine at the high dose; however, dopamine had a greater effect than norepinephrine (Fig. 4b and Supplementary Fig. 16). Additionally, we observed that Acidobacteria significantly decreased in the soil treated with the high dose of norepinephrine compared to those in the control soil, but there was no significant difference in the relative abundance of Acidobacteria between treatments with dopamine and norepinephrine (Supplementary Fig. 16). These results underscore the selective impact of these metabolites, particularly dopamine, on soil Actinobacteria communities.

The treatment with dopamine and norepinephrine also resulted in characteristic changes at the zOTU level within multiple bacterial phyla. With principal component analysis, a notable trend was detected among zOTUs with relative abundance ≥0.1%, represented by principal component 1 (PC1). The responses of zOTUs to treatments were divided into two groups: the first group included dopamine (both low and high) and norepinephrine_High, while the second group consisted of the Control and norepinephrine_Low (Fig. 4c). Further statistical analysis identified eight sensitive zOTUs that statistically (|loading score | ≥ 0.10) contributed to this trend, and some of them were differentially enriched among treatments (*P* < 0.05) (Supplementary Fig. 17), suggesting that these metabolites had a specific role in modulating the soil microbial communities. The relative abundances of Actinobacteria_zOTU1 and Actinobacteria_zOTU3 were significantly increased by both dopamine_High and norepinephrine_High (Fig. 4c and Supplementary Fig. 17). Additionally, Actinobacteria_zOTU2 and Actinobacteria_zOTU4 were enriched by dopamine_High and norepinephrine_High, respectively. Conversely, Proteobacteria*_*zOTU4, Bacteriodetes_zOTU1, Thaumarchaeota_zOTU1, and Actinobacteria_zOTU7 were more abundant in the Control and low norepinephrine treatments and were negatively impacted by high dopamine (Fig. 4c, Supplementary Fig. 17). Taken together, these results suggest that treatment with these root-derived metabolites, particularly dopamine, specifically altered the bacterial community structures in the soil.

## Discussion

Root exudates play a crucial role in mediating interactions between plants and soil microbiota^8,30^. Genetic variation in root exudation is a key factor in the adaptability and ecological success of plants, influencing both individual plant growth and broader ecosystem dynamics^31,32^. In this study, we investigated the natural variation in root exudates of diverse *B. distachyon* lines and their influence on the composition of a synthetic microbial community in a hydroponic system. Our findings demonstrate the significant impact of intraspecific root exudate variation on shaping the composition and activity of a synthetic microbial community. The observed differences in exudate metabolic profiles across diverse *Brachypodium* lines underscore the role of genetic variation in governing root exudate composition, which subsequently influences microbial recruitment and community assembly, thereby contributing to the establishment of distinct root microbiome profiles. Through correlation analysis of exudate metabolomics and 16S rRNA sequencing data, we identified a dominant root exometabolite, dopamine, which significantly impacts soil microbial communities. Our results align with previous studies demonstrating the importance of plant genetic variation in modulating root exudate composition and subsequently affecting microbiome dynamics^33–35^. Understanding the natural variation in root exometabolites and its link to microbiome structure provides valuable insights into plant-microbiome interactions and can guide strategies for harnessing microbial consortia to enhance plant health and productivity in agricultural systems.

The distinct metabolite profiles and SynCom compositions observed across these lines suggest that plant genotype influences both exudate chemistry and microbial recruitment. Additionally, to minimize environmental variability, we conducted all experiments under controlled hydroponic conditions, ensuring uniform nutrient availability, temperature, and light exposure. While hydroponic systems provide a controlled environment, they do not fully replicate soil complexity. To address this, we complemented our findings with soil treatment experiments, which further validated dopamine’s role in shaping microbial communities. These complementary approaches strengthen our conclusions by demonstrating that dopamine-mediated microbial shifts are observed across different conditions.

Integrative analysis of microbiome-metabolome data sets has proven to be the most promising strategy for the discovery of microbe-metabolite links in the human gut^36,37^. In this study, we demonstrate the utility of integrative analysis for uncovering microbe-metabolite links in the model grass *B. distachyon*. We first incubated diverse *Brachypodium* lines with a 16-member SynCom in a hydroponic system and collected exudate metabolomic data and 16S rRNA sequencing data. Using this data, we identified a dominant exometabolite, dopamine, in exudates that significantly associated with the abundance of six SynCom members (Fig. 3). The use of a hydroponic system in this study was crucial because it provided a controlled environment, facilitated exudate collection, and ensured adequate growth and sampling opportunities for the SynCom members^12–14^, enabling robust associations between metabolites and microbes. Though there are limitations in the correlation-based analysis to identify key microbiome-metabolite links, statistically significant correlations can be valuable for generating hypotheses and directing experimental research efforts. We acknowledge that dopamine levels measured under axenic conditions exhibited high variability. Root exudation is increasingly recognized as a dynamic trait influenced by plant genotype, developmental stage, environmental conditions, and interactions with microorganisms^38^. Therefore, the variability observed in dopamine exudation across independent experiments may reflect the combined effects of genetic factors, environmental influences, and genotype-by-environment interactions, rather than a strictly genotype-determined trait. Despite this, dopamine levels showed significant differences between certain lines in the presence of the SynCom (Fig. 2b). To further examine the role of dopamine in plant-microbiome interactions, we performed in vitro bacterial growth and soil treatment assays (Figs. 3, 4), both of which support the importance of dopamine in modulating soil microbial communities.

Functional tests in soil revealed that catecholamine-related metabolites, including dopamine and norepinephrine, can restructure soil microbial communities, particularly by enriching Actinobacteria populations. Although both compounds induced partially overlapping responses at the phylum level, deeper community analyses revealed distinct treatment-specific effects at the zOTU and β-diversity levels, suggesting that structurally related catecholamines may exert both shared and compound-specific influences on rhizosphere microbial communities. Importantly, dopamine was consistently detected at high abundance in both roots and root exudates, whereas norepinephrine was detected only at trace levels in roots and was not reliably detected in root exudates under our experimental conditions. This difference in endogenous abundance limited our ability to perform comparable correlation analyses for norepinephrine and supports the emphasis on dopamine as the dominant detectable catecholamine associated with microbial community variation in *B. distachyon*.

Actinobacteria play essential roles in soil ecosystems, contributing to organic matter decomposition, nutrient cycling, and plant growth promotion^39^. Many Actinobacteria species, such as Streptomyces, produce bioactive compounds that enhance plant disease resistance and improve soil health. Additionally, Actinobacteria are known to be enriched under drought conditions, suggesting a potential role in stress adaptation^39,40^. Therefore, their observed enrichment in dopamine-treated soil may provide a mechanism for increasing these beneficial effects. Although Actinobacteria include many taxa with well-documented beneficial traits^39^, functional validation of dopamine-driven enrichment remains to be established and will be an important direction for future investigation. The underlying mechanism of dopamine modulating rhizosphere microbiota could be similar to that involved in regulating human gut microbial growth, such as affecting gene expression and biofilm formation^23,35^. Dopamine has been shown to modulate quorum sensing pathways in bacteria, which regulate biofilm formation and stress responses^41^. This suggests that dopamine could influence microbial community assembly by altering microbial gene expression and interspecies interactions. Most Actinobacteria are known to produce extracellular polysaccharides and secondary metabolites that contribute to biofilm development and microbial resilience^39^. Additionally, the ability of dopamine to influence Actinobacteria abundance may stem from its role as a potential carbon and nitrogen source and/or its redox properties that affect microbial competition and survival in the rhizosphere^42^. Further studies are needed to determine the precise bacterial pathways regulated by dopamine and their functional consequences in the rhizosphere.

Actinobacteria are known for their diverse metabolic capabilities and beneficial roles in plant health. Previous studies revealed the enrichment of Actinobacteria in drought-treated soils across various environments^43,44^, within drought-affected rhizospheres^45,46^, and within the host roots^40^, suggesting that Actinobacteria play an important role in plant stress response. Recently, two Actinobacteria (*Pseudarthrobacter* sp. L1D14 and *Pseudarthrobacter picheli* L1D33) were reported to promote plant growth in a hydroponic system^47^. This study extends these findings by demonstrating dopamine-mediated enrichment of Actinobacteria in both hydroponic and soil environments, suggesting a potential mechanism for plants to modulate soil microbial communities.

Plant root exudates are complex mixtures of metabolites that collectively influence rhizosphere microbial communities. While our study focuses on dopamine, its interactions with other exuded compounds may play a crucial role in shaping microbial recruitment and activity. Flavonoids, phenolic acids, organic acids, and amino acids are among the key metabolites found in root exudates, each contributing to microbial signaling, gene regulation, and metabolic adaptation^8^. These metabolites can modulate microbial responses to dopamine by affecting its bioavailability, degradation, or uptake pathways^48^. For example, phenolic compounds with redox activity may interact with dopamine, altering its chemical stability and microbial utilization. Additionally, organic acids secreted by roots can influence soil pH and metal availability, indirectly impacting microbial community composition and dopamine-mediated effects^42^. By considering dopamine in the broader context of root exudate chemistry, we propose that its selective enrichment of specific microbial taxa, such as Actinobacteria, may be influenced by co-exuded metabolites that act synergistically or antagonistically. Future studies are needed to disentangle these complex metabolite-microbe interactions and uncover how plants fine-tune their rhizosphere microbiomes through diverse chemical cues.

Our findings provide new insights into the potential role of dopamine in shaping rhizosphere microbial communities, reinforcing the growing recognition of plant-derived secondary metabolites as important mediators of plant-microbiome interactions^42^. Our results further suggest a complex and dynamic interaction in which dopamine both influences and is influenced by rhizosphere microbial communities. These findings have significant implications for agriculture and ecology, as leveraging plant-derived metabolites could provide new strategies for promoting beneficial microbes, enhancing nutrient cycling, and improving plant stress tolerance^49^. Understanding the ecological roles of dopamine in the rhizosphere may also contribute to the development of bioinoculants or microbiome engineering approaches for improving crop resilience under climate change conditions. Although our findings suggest that dopamine plays a role in modulating rhizosphere microbial communities, further experimental validation is needed to confirm its functional effects on microbial physiology and community dynamics, particularly under natural field conditions.

## Methods

### Plant materials and plant growth

Ten inbred *B. distachyon* lines (Adi9, ABR6, Bd1-1, Bd2-3, Bd21, Bd21-3, Bd3-1, Bd30-1, BdTR10h, and BdTR1f) were used in this study^50–53^. Dehusked seeds were surface sterilized in 70% (v/v) ethanol for 30 s and 5 min in 6% (w/v) sodium hypochlorite, followed by five washes with sterile water. The seeds were subsequently stratified on 1.5% (w/v) agar plates containing 0.5 × Murashige & Skoog (0.5 × MS salts, MSP01, Caisson Laboratories, USA) and placed in darkness at 4 °C for three days. The plates containing the seeds were then moved to a growth chamber with a temperature of 22 °C, photosynthetic photon flux density at 150 μmol m^−2^ s^−1^, and a 16 h light/8 h dark photoperiod for two days.

The culture vessels (PTL-100™, PhytoTech Labs) were rinsed five times with MilliQ water and autoclaved. Two seedlings of *B. distachyon* were placed onto a plastic floating holder and then transferred to each vessel with 40 mL of 0.5 × MS. Plants were grown in a 16 h light/8 h dark regime at 22 °C and 50% relative humidity with 150 μmol m^−2^ s^−1^ illumination for four weeks. The culture vessels without plants filled with the growth medium (0.5 × MS salts) were incubated in the same conditions as the controls. For plant growth with dopamine (Sigma-Aldrich), seven-day-old *B. distachyon* Bd21-3 seedlings were treated with dopamine at concentrations of 25, 50, and 100 μM for three weeks. Bd21-3 was selected for the follow-up experiments primarily because it is one of the most widely used reference lines and is well-suited for hydroponic growth and physiological characterization. In week 4, *B. distachyon* Bd21-3 plants were harvested and photographed. Root and shoot fresh biomass were measured. Root length and shoot length were quantified using the SmartRoot plugin (version 4.21) in ImageJ (version 2.0.0). The sterility of the hydroponic setup was examined before exudate collection by plating 50 μL of medium on Luria-Bertani (LB) plates, followed by seven-day incubation at 30 °C.

### Bacterial strains and synthetic community inoculation

Sixteen bacterial strains used in this study were isolated from the rhizosphere and bulk soil surrounding a *Panicum virgatum* (switchgrass) plant in Oklahoma, United States^29^. Strains were cultured at 30 °C and 200 rpm in the following liquid media: *Lysobacter* OAE881, *Burkholderia* OAS925, *Variovorax* OAS795, *Chitinophaga* OAE865, *Bosea* OAE506, *Rhodococcus* OAS809, *Marmoricola* OAE513, *Paenibacillus* OAE614, *Methylobacterium* OAE515, *Arthrobacter* OAP107, *Mucilaginibacter* OAE612, and *Brevibacillus* OAP136 in Reasoner’s 2 A (R2A) medium^54,55^, *Niastella* OAS944, *Rhizobium* OAE497, and *Mycobacterium* OAE908 in 0.1 × R2A, and *Bacillus* OAE603 in Luria-Bertani (LB) medium (Thermo Fisher, USA) (Supplementary Data 1).

Bacterial cultures were centrifuged at 3600 *g* for 20 min and washed 3 times with 0.5× MS. Individual bacterial strains were resuspended to have an OD_600_ at 1.0, and then equal volumes were pooled together to make the 16-member SynCom. The SynCom inoculant (800 μL) was applied to the 0.5× MS growth medium of half of the culture vessels containing *B. distachyon* (*n* = 2 or 3) at week one to have a final OD_600_ of approximately 0.02. The SynCom inoculant was also added to the vessels filled with plant-spent medium without plants (No Plants). The vessels containing only 0.5× MS growth medium were used as technical controls (TeCtrl). Following inoculation, plants were grown for an additional 3 weeks under a 16 h light/8 h dark cycle, 22 °C, and 50% humidity until harvest.

### Exudate harvesting and bacterial cell collection

Four-week-old plants were carefully removed from the culture vessel together with the floating holder, and the plant spent medium was collected into a 50 mL Falcon centrifuge tube. OD_600_ was measured to determine the SynCom bacterial growth. The plant-spent medium was then centrifuged at 3600 *g* for 20 min to collect bacterial cells. The supernatants were filtered with 0.2 μm PES membrane filters (Pall Corporation, NY, USA), freeze-dried (Labconco Freeze-Zone), and stored at −80 °C for metabolite analysis. The bacterial pellets were resuspended in 20% glycerol and stored at −80 °C prior to DNA extraction.

### LC-MS/MS analyses of *B. distachyon* root exudates

The plant spent media from 4-week-old plants (approximately 30 mL) were frozen at −80 °C and subsequently freeze-dried using a Labconco Freeze-Zone. The dried material was resuspended by rinsing the tubes with 1 mL of LC-MS grade methanol (Sigma-Aldrich), which was then transferred to 2-mL tubes and dried in a Thermo Speed-Vac concentrator. The dried material was then resuspended in 300 μL of LC-MS grade methanol containing internal standards. The solution was vortexed for 2 × 10 s, bath-sonicated in ice water for 15 min, and centrifuged at 10,000 g for 5 min at 10 °C to pellet the insoluble materials. The supernatants were then filtered using 0.22-μm polyvinylidene difluoride microcentrifuge filtration devices (Pall Corporation, NY, USA) at 10,000 *g* for 5 min at 10 °C. The filtrates were used for metabolite analysis. Polar metabolites were separated using hydrophilic interaction chromatography (HILIC) and detected on a Thermo Q Exactive Hybrid Quadrupole-Orbitrap Mass Spectrometer. Briefly, an InfinityLab Poroshell 120 HILIC-Z, 2.1 × 150 mm, 2.7 μm column equipped on an Agilent 1290 HPLC stack was used for separations. Data was collected using data-dependent MS2 acquisition to select the top two most intense ions not previously fragmented within 7 s. Internal and external standards were used for quality control purposes. Method parameters are defined in Supplementary Data 6.

### Dopamine identification using LC-MS/MS

Dopamine was identified in *B. distachyon* root exudates and tissues using high-resolution LC-MS/MS. Sample extracts were separated using a HILIC column and then analyzed on a Thermo Q Exactive Orbitrap mass spectrometer (See Supplementary Data 6 for details on the LC-MS/MS conditions). The in-source fragment of dopamine (*m*/*z* 137.0597, [M + H - NH_3_]^+^) was observed to be one of the major MS1 ions and was used for relative quantification. The dopamine precursor ion at *m*/*z* 154.0862 ([M + H]^+^) was also detected but was consistently less abundant in the MS1 scans. Dopamine identification was confirmed based on matching the exact mass, retention time, and MS/MS fragmentation pattern to an authentic dopamine standard (Sigma-Aldrich) analyzed under identical conditions. Additional confirmation was provided by spiking [ring-^13^C_6_]-labeled dopamine (Cambridge Isotope Laboratories) into the extraction buffer as an internal standard. Base peak chromatograms and extracted ion chromatograms were prepared using the FreeStyle software (FreeStyle™ 1.5, Thermo Fisher Scientific).

In addition, Metatlas (https://github.com/biorack/metatlas) was used to compare dopamine *m*/*z*, retention time, and fragmentation spectra from samples to the reference standard^56^. Annotation evidence is provided in Supplementary Data 7.

### Untargeted metabolomics analysis

The MZmine2 version 2.39 workflow was used for picking up features for positive and negative polarities^57^. A baseline filter was initially performed, which accepted features with a retention time (RT) of >0.6 min, a maximum peak height of >1 × 10^6^, and >10 x the maximal peak height in extraction (ExCtrl, *n* = 3) and technical controls (TeCtrl, *n* = 3). To remove the metabolites from the SynCom bacterial members, features were further filtered to remove those with a maximum peak height of >1 × 10^6^ detected in No Plants controls which were filled with a root spent medium and the SynCom. We added +0.1 to all filtered features during the fold change calculations to avoid errors when dividing by zero. These parameters were used for feature filtering for all *Brachypodium* lines in the presence and absence of the SynCom. Non-metric multidimensional scaling (NMDS) analysis was performed to visualize differences in exudate profiles of ten different *B. distachyon* lines in the absence and presence of the SynCom. NMDS analysis was conducted using the *metaMDS* function from the *vegan* R package, and the resulting ordination plot was generated using *ggplot2* in R.

### Correlation analysis of dopamine with the SynCom members and plant phenotypes

To determine the covariance between dopamine and the composition of the microbial communities in the plant-spent media, we performed association analysis based on the relative levels of dopamine (ion intensity of 137.0547-137.0647 *m*/*z*, [M + H - NH_3_]^+^) and the relative abundances of SynCom members in the plant-spent medium of the ten *B. distachyon* lines. Spearman correlations and *P*-values were calculated using the *rcorr* function of the R package *Hmisc* for dopamine-SynCom member pairs. Positive and negative correlations correspond to positive and negative links, respectively. Additionally, Spearman correlations and *P*-values were also calculated for dopamine-phenotype pairs.

### Soil treatment

Soil (Yolo silt loam) was collected from an agricultural field at UC Davis (38°32'16.9“N, 121°46'00.2“W). After collection, the soil was dried at room temperature and stored at 4 °C for 2–3 months, and then sieved (2 mm mesh) before starting the experiment. The metabolites (dopamine and norepinephrine) at two doses (1.5 mg and 3.0 mg) were directly mixed into 20 g of dried soil without solvent dissolution, and then 6.5 mL of water was added to achieve a water content of approximately 24.5% by weighing each replicate and adding water accordingly, and the treatments were noted with dopamine_Low, dopamine_High, norepinephrine_Low, and norepinephrine_High, respectively. The treatments were repeated twice a week over a period of 6 weeks, and the water content was maintained at approximately 24.5%. Control soil received the same handling and water addition but without the addition of dopamine or norepinephrine. The soil was covered with foil and incubated at room temperature. Three replicates were prepared per treatment. Soil samples were harvested at week 6 and stored at −20 °C prior to DNA extraction.

### DNA extraction, 16S rRNA amplicon generation, and sequencing

Total DNA was extracted from samples using the FastDNA SPIN Kit for Soil (MP Biomedicals, United States) as described in the manufacturer’s protocol. Genomic DNA was then eluted in 40 μL of nuclease-free water. The V4/V5 region of the 16S rRNA gene was amplified by PCR from each of the purified DNA samples using the 515 F/926 R primers based on those from the Earth Microbiome Project^58,59^, but with in-line dual Illumina indexes. The resulting amplicons were sequenced on an Illumina MiSeq using 600 bp v3 reagents. Custom Perl scripts were employed for read processing, which included merging with Pear^60^, filtering reads (zero-radius operational taxonomic units, zOTUs) with more than one expected error using Usearch^61^, demultiplexing using inline indexes, and filtering rare reads and chimeras with Unoise^62^. 16S sequences were determined by comparing them against the RDP database for taxonomy assignment^63^. In this study, zOTU is interchangeable with ESV (exact sequence variant) and ASV (amplicon sequence variant).

### Processing and statistical analysis of 16S rRNA counts

Phylum-level tables were generated based on the taxonomic classifications performed above, with all zOTU counts assigned to a given phylum summed in the Supplementary Data 5. The denoised table of zOTU counts and phylum tables were trimmed to remove low abundance (i.e., zOTUs only present in 1 or 2 samples with fewer than 10 reads). The resulting datasets were modeled and then analyzed using the DESeq2 software program with default settings. Pairwise comparisons were conducted using the contrast function across all combinations of treatments. Comparisons with an adjusted *P*-value greater than 0.05 were discarded. Hierarchical clustering was performed using the Seaborn package in Python with default parameters. Notably, Bd21 16S rRNA data is limited to a single replicate, whereas other lines had two or three replicates available.

### Quantification of dopamine in *Brachypodium* roots

*Brachypodium* Bd21-3 root tissues were harvested from 4-week-old hydroponically grown plants and stored at −80 °C for further analysis. Before extraction, root tissues (approximately 0.1 g fresh weight) were ground into powder using bead milling. 500 μL extraction buffers [95% (70% methanol, methanol:H_2_O, 70:30, v/v) and 5% 1 M hydrochloric acid] with a [ring-^13^C_6_]-labeled dopamine (Cambridge Isotope Laboratories) at concentrations of 0.5 mM, 1.0 mM, 2.0 mM, 4.0 mM, and 8.0 mM were then added into the root powders (*n* = 2) for dopamine extraction overnight at 4 °C. This approach enables accurate quantification of endogenous dopamine within a dynamic range of 0.38 to 6.13 mg/g fresh tissues. The samples were subsequently centrifuged for 10 min at 15,000 g. The supernatants were further filtered using 0.22-μm polyvinylidene difluoride microcentrifuge filtration devices (Pall Corporation, NY, USA) by centrifuging (10,000 *g* for 5 min at 10 °C) and diluted 100-fold before LC-MS/MS analysis using hydrophilic interaction chromatography (HILIC) on a Thermo Q Exactive Hybrid Quadrupole-Orbitrap Mass Spectrometer.

The dopamine calibration curve was obtained by analyzing the above samples on LC-MS/MS. The analytical curve was described by a linear regression model *y* = **a***x* + **b**, where *y* represents the analytical response, *x* is the concentration of analyte in μM, **a** denotes the slope of the curve, and **b** is the intercept. The calibration curve equation and the *R*^2^ determination were established based on the linear regression of the peak height of the [ring-^13^C_6_]-labeled dopamine (*m*/*z*, 143.0746-143.0846, [M + H - NH_3_]^+^) over its concentration. The calibration curve (*y* = 5.69E5*x* + 4.26E5) showed good linearity in the range of 5.0–80.0 μM tested with the coefficient of determination *R*^2^ = 0.9996 (Supplementary Fig. 12a). The levels of endogenous dopamine (*m*/*z*, 137.0547-137.0647, [M + H - NH_3_]^+^) in *Brachypodium* roots were calculated based on the [ring-^13^C_6_]-labeled dopamine calibration curve (*n* = 12). The plant endogenous dopamine concentrations were converted to milligrams of ^12^C-dopamine per gram fresh weight.

### In vitro bacterial growth

In vitro bacterial growth assays with dopamine were performed using the Clinical and Laboratory Standards Institute M38-A2 guidelines^64^. In brief, a 96-well microtiter plate-based method using a Synergy4 (BioTech Instruments) reader was used to monitor bacterial growth at 30 °C in liquid media through periodic measurements of changes in optical density (OD_600_) for 72 h. Bacterial cultures were centrifuged at 3600 *g* for 20 min and washed 3 times with the corresponding growth media. Each plate well contained 200 µL of initial bacterial inoculum (OD_600_ at approximately 0.01) supplemented with dopamine at two different concentrations (50 and 100 µg/mL). Methanol was used as a solvent for making dopamine solutions. Our controls were blank medium and bacterial-inoculated medium with 0.1% methanol (*n* = 4).

### Statistical analysis

Statistical analyses were conducted using JMP Pro v.13.0 (SAS Institute) and Prism v.9.0 (GraphPad). Group differences were assessed using one-way ANOVA when variances were homogeneous and Welch’s ANOVA when variances were unequal, followed by Tukey’s or Dunnett’s T3 post hoc tests, respectively. Welch’s ANOVA and Dunnett’s T3 tests were used for datasets exhibiting heteroscedasticity, including root exudate metabolite measurements, because these approaches do not assume equal variances among groups and are more robust for datasets with unequal variances and modest sample sizes. Statistical analyses for dopamine measurements under axenic and SynCom conditions were conducted separately to evaluate variation among plant lines within each biological condition independently. Student’s unpaired two-tailed *t*-tests were conducted for pairwise comparisons. A *P*-value of <0.05 was considered to be statistically significant.

The beta-diversity of soil microbial communities was calculated as Bray-Curtis Dissimilarity using the *vegan* package, and permutational multivariate analysis of variance (PERMANOVA) was performed using the *adonis2* package to test their significant differences between treatments, both done in the R environment (version 4.4.1). The sensitive zOTUs were selected using the modified selection pipeline^65^. Briefly, principal component analysis was performed on zOTUs with maximum relative abundance of at least 0.1%, after scaling their relative abundances using the package *zCompositions*. The principal component (PC) scores were analyzed with one-way ANOVA and PC with significant differences between treatments (*P* < 0.05) were selected. Then, zOTUs with an absolute value of the loading score larger than |0.1| were selected as sensitive taxa. The lsmeans of PC scores for each treatment were multiplied by the loading scores of each sensitive taxa to visualize their responses to the treatments. Then, hypothesis testing was performed on the relative abundances of these sensitive taxa by treatment using one-way ANOVA and post-hoc Tukey’s test. The figures for PERMANOVA and sensitive taxa were created using *ggplot2* in the R environment^66^.

### Reporting summary

Further information on research design is available in the Nature Portfolio Reporting Summary linked to this article.

## Supplementary information


Supplementary Information
Description of Additional Supplementary Information
Supplementary Data 1
Supplementary Data 2
Supplementary Data 3
Supplementary Data 4
Supplementary Data 5
Supplementary Data 6
Supplementary Data 7
Reporting Summary
Transparent Peer Review file


## Source data


Source data


## Data Availability

The LC-MS/MS raw files generated in this study have been deposited in the Mass Spectrometry Interactive Virtual Environment (MassIVE, https://massive.ucsd.edu/) under accession number MSV000095543. The exudate LC-MS/MS dataset from the ten *Brachypodium* lines grown under axenic conditions with six replicates has been deposited in GNPS2. The 16S rRNA gene sequencing datasets have been deposited in the NCBI Sequence Read Archive (SRA) under BioProject PRJNA1484685. Source data are provided with this paper.

## Code availability

All software packages utilized in this study are publicly available and are described in the Methods. Custom Perl scripts used in this study are available upon request.
