# Supplementary material for: Insights into the role of dopamine in rhizosphere microbiome assembly
Source: Nat Commun. 2026 Jul 29;17:9212. doi: 10.1038/s41467-026-76211-1 (PMC13522444; doi:10.1038/s41467-026-76211-1)
Supplement: Supplementary file 1 [file 41467_2026_76211_MOESM10_ESM.pdf]

nature portfolio

Corresponding author(s): Trent R. Northen, John P. Vogel, Yezhang Ding

Last updated by author(s): Sep 24, 2025

Reporting Summary

Nature Portfolio wishes to improve the reproducibility of the work that we publish. This form provides structure for consistency and transparency in reporting. For further information on Nature Portfolio policies, see our Editorial Policies and the Editorial Policy Checklist.

Statistics

For all statistical analyses, confirm that the following items are present in the figure legend, table legend, main text, or Methods section.

n/a	Confirmed	
☐	☒	The exact sample size (*n*) for each experimental group/condition, given as a discrete number and unit of measurement
☐	☒	A statement on whether measurements were taken from distinct samples or whether the same sample was measured repeatedly
☐	☒	The statistical test(s) used AND whether they are one- or two-sided<br>*Only common tests should be described solely by name; describe more complex techniques in the Methods section.*
☒	☐	A description of all covariates tested
☐	☒	A description of any assumptions or corrections, such as tests of normality and adjustment for multiple comparisons
☐	☒	A full description of the statistical parameters including central tendency (e.g. means) or other basic estimates (e.g. regression coefficient) AND variation (e.g. standard deviation) or associated estimates of uncertainty (e.g. confidence intervals)
☐	☒	For null hypothesis testing, the test statistic (e.g. *F*, *t*, *r*) with confidence intervals, effect sizes, degrees of freedom and *P* value noted<br>*Give P values as exact values whenever suitable.*
☒	☐	For Bayesian analysis, information on the choice of priors and Markov chain Monte Carlo settings
☒	☐	For hierarchical and complex designs, identification of the appropriate level for tests and full reporting of outcomes
☒	☐	Estimates of effect sizes (e.g. Cohen's *d*, Pearson's *r*), indicating how they were calculated

Our web collection on statistics for biologists contains articles on many of the points above.

Software and code

Policy information about availability of computer code

Data collection	LC–MS/MS data were acquired on a Thermo Q Exactive Hybrid Quadrupole-Orbitrap mass spectrometer operating in data-dependent MS^2 mode, with the two most intense precursor ions sequentially selected for fragmentation if not already fragmented within the preceding 7 s.
Data analysis	Data and statistical analyses were conducted using GraphPad Prism v10.2.3 (GraphPad Software, Inc.) and JMP Pro v15.0 (SAS Institute Inc.).For metabolite analyses, raw LC-MS/MS data were processed using the MetAtlas workflow (https://github.com/biorack/metatlas) for feature detection and annotation. Extracted ion chromatograms (EICs) and tandem MS spectra were generated using Thermo FreeStyle software. Dopamine and related metabolites were identified by comparing retention times, accurate masses, and MS/MS fragmentation patterns against authentic standards. Quantification was performed using calibration curves with linear regression models. Metabolite levels were normalized to tissue fresh weight. Group differences were assessed using one-way ANOVA when variances were homogeneous and Welch's ANOVA when variances were unequal, followed by Tukey's or Dunnett's T3 post hoc tests, respectively. Student's unpaired two-tailed t-tests were conducted for pairwise comparisons. A P-value of < 0.05 was considered to be statistically significant. Perl scripts were employed for 16 S rRNA sequence read processing, which included merging with Pear, filtering reads (zero-radius operational taxonomic units, zOTUs) with more than one expected error using Usearch, demultiplexing using inline indexes, and filtering rare reads and chimeras with Unoise. 16S sequences were determined by comparing them against the RDP database for taxonomy assignment. The beta-diversity of soil microbial communities was calculated as Bray-Curtis Dissimilarity using the vegan package, and permutational multivariate analysis of variance (PERMANOVA) was performed using the adonis2 package to test their significant differences between treatments, both done in the R environment (version 4.4.1.). The sensitive zOTUs were selected using the modified selection pipeline. Briefly, principal component analysis was performed on zOTUs with maximum relative abundance of at least 0.1%, after scaling their relative abundances using the package zCompositions. The principal component (PC) scores were analyzed with one-way ANOVA and PC with significant differences between treatments (P-value < 0.05) were selected. Then, zOTUs with an absolute value of the loading score larger than |0.1| were selected as sensitive taxa. The lsmeans of PC scores for each treatment were multiplied by the loading scores of each sensitive taxa to visualize their

responses to the treatments. Then, hypothesis testing was performed on the relative abundances of these sensitive taxa by treatment using one-way ANOVA and post-hoc Tukey's test. The figures for PERMANOVA and sensitive taxa were created using ggplot2 in the R environment. Spearman correlations and P-values were calculated using the rcorr function of the R package Hmisc for dopamine-SynCom member pairs.

For manuscripts utilizing custom algorithms or software that are central to the research but not yet described in published literature, software must be made available to editors and reviewers. We strongly encourage code deposition in a community repository (e.g. GitHub). See the Nature Portfolio guidelines for submitting code & software for further information.

Data

Policy information about availability of data

All manuscripts must include a data availability statement. This statement should provide the following information, where applicable:
- Accession codes, unique identifiers, or web links for publicly available datasets
- A description of any restrictions on data availability
- For clinical datasets or third party data, please ensure that the statement adheres to our policy

The LC-MS/MS raw files generated in this study have been deposited in the Mass Spectrometry Interactive Virtual Environment (MassIVE, https://massive.ucsd.edu/) under accession number MSV000095543 [https://massive.ucsd.edu/ProteoSAFe/dataset.jsp?accession=MSV000095543]. The exudate LC-MS/MS dataset from the ten Brachypodium lines grown under axenic conditions with six replicates has been deposited in GNPS2 [https://gnps2.org/result?task=69201e7e49004f6689bfff298c1d0327&viewname=filesummary&resultdisplay_type=task]. The 16S rRNA gene sequencing datasets have been deposited in the NCBI Sequence Read Archive (SRA) under BioProject PRJNA1484685 [https://www.ncbi.nlm.nih.gov/sra/PRJNA1484685]. Source data are provided with this paper.

Research involving human participants, their data, or biological material

Policy information about studies with human participants or human data. See also policy information about sex, gender (identity/presentation), and sexual orientation and race, ethnicity and racism.

Reporting on sex and gender	*Use the terms sex (biological attribute) and gender (shaped by social and cultural circumstances) carefully in order to avoid confusing both terms. Indicate if findings apply to only one sex or gender; describe whether sex and gender were considered in study design; whether sex and/or gender was determined based on self-reporting or assigned and methods used.*<br>*Provide in the source data disaggregated sex and gender data, where this information has been collected, and if consent has been obtained for sharing of individual-level data; provide overall numbers in this Reporting Summary. Please state if this information has not been collected.*<br>*Report sex- and gender-based analyses where performed, justify reasons for lack of sex- and gender-based analysis.*
Reporting on race, ethnicity, or other socially relevant groupings	*Please specify the socially constructed or socially relevant categorization variable(s) used in your manuscript and explain why they were used. Please note that such variables should not be used as proxies for other socially constructed/relevant variables (for example, race or ethnicity should not be used as a proxy for socioeconomic status).*<br>*Provide clear definitions of the relevant terms used, how they were provided (by the participants/respondents, the researchers, or third parties), and the method(s) used to classify people into the different categories (e.g. self-report, census or administrative data, social media data, etc.)*<br>*Please provide details about how you controlled for confounding variables in your analyses.*
Population characteristics	*Describe the covariate-relevant population characteristics of the human research participants (e.g. age, genotypic information, past and current diagnosis and treatment categories). If you filled out the behavioural & social sciences study design questions and have nothing to add here, write "See above."*
Recruitment	*Describe how participants were recruited. Outline any potential self-selection bias or other biases that may be present and how these are likely to impact results.*
Ethics oversight	*Identify the organization(s) that approved the study protocol.*

Note that full information on the approval of the study protocol must also be provided in the manuscript.

Field-specific reporting

Please select the one below that is the best fit for your research. If you are not sure, read the appropriate sections before making your selection.

[x] Life sciences [ ] Behavioural & social sciences [ ] Ecological, evolutionary & environmental sciences

For a reference copy of the document with all sections, see nature.com/documents/nr-reporting-summary-flat.pdf

Life sciences study design

All studies must disclose on these points even when the disclosure is negative.

Sample size	No specific statistical methods were used to determine sample size for the experiments reported in this manuscript. Instead, sample sizes were chosen based on prior studies, previous experience, variability encountered, availability of plants and space considerations.
Data exclusions	No data were excluded from analysis in this study.
Replication	When technical problems were eliminated and methods described were followed all experiments could be reliably reproduced.

Randomization	Plants were grown in the growth chambers and randomly used for experiments.
Blinding	The experiments did not involve blinding in this study.

Reporting for specific materials, systems and methods

We require information from authors about some types of materials, experimental systems and methods used in many studies. Here, indicate whether each material, system or method listed is relevant to your study. If you are not sure if a list item applies to your research, read the appropriate section before selecting a response.

Materials & experimental systems

n/a	Involved in the study
☒	☐ Antibodies
☒	☐ Eukaryotic cell lines
☒	☐ Palaeontology and archaeology
☒	☐ Animals and other organisms
☒	☐ Clinical data
☒	☐ Dual use research of concern
☐	☒ Plants

Methods

n/a	Involved in the study
☒	☐ ChIP-seq
☒	☐ Flow cytometry
☒	☐ MRI-based neuroimaging

Dual use research of concern

Policy information about dual use research of concern

Hazards

Could the accidental, deliberate or reckless misuse of agents or technologies generated in the work, or the application of information presented in the manuscript, pose a threat to:

No	Yes
☒	☐ Public health
☒	☐ National security
☒	☐ Crops and/or livestock
☒	☐ Ecosystems
☒	☐ Any other significant area

Experiments of concern

Does the work involve any of these experiments of concern:

No	Yes
☒	☐ Demonstrate how to render a vaccine ineffective
☒	☐ Confer resistance to therapeutically useful antibiotics or antiviral agents
☒	☐ Enhance the virulence of a pathogen or render a nonpathogen virulent
☒	☐ Increase transmissibility of a pathogen
☒	☐ Alter the host range of a pathogen
☒	☐ Enable evasion of diagnostic/detection modalities
☒	☐ Enable the weaponization of a biological agent or toxin
☒	☐ Any other potentially harmful combination of experiments and agents

Plants

Seed stocks	Seeds of B. distachyon accessions used in this study were obtained from the Vogel lab at Joint Genome Institute.
Novel plant genotypes	NONE
Authentication	NONE
