# Supplementary material for: Insights into the role of dopamine in rhizosphere microbiome assembly
Source: Nat Commun. 2026 Jul 29;17:9212. doi: 10.1038/s41467-026-76211-1 (PMC13522444; doi:10.1038/s41467-026-76211-1)
Supplement: Supplementary file 2 [file 41467_2026_76211_MOESM11_ESM.pdf]

nature portfolio

# Peer Review File

# Insights into the role of dopamine in rhizosphere microbiome assembly

Corresponding Author: Dr Yezhang Ding

**This file contains all reviewer reports in order by version, followed by all author rebuttals in order by version.**

Version 0:

Reviewer comments:

Reviewer #1

(Remarks to the Author)
This study delves into the role of dopamine in the assembly of the plant rhizosphere microbiome. It was discovered that dopamine levels in root exudates vary significantly across different Brachypodium distachyon lines and were notably associated with the abundance of six members of a synthetic community (SynCom). Further in vitro growth studies had demonstrated that dopamine significantly influences the growth of these six bacterial isolates. Soil treatment experiments had shown that the direct application of dopamine enriches for Actinobacteria, aligning with the SynCom-dopamine correlations and isolate growth outcomes. The research underscores the selective impact of dopamine on rhizosphere microbial communities, offering potential for precision microbiome management.
Advantages:
1、The study introduces a pioneering and compelling investigation into the role of dopamine in the assembly of the plant rhizosphere microbiome, thereby opening new frontiers for research within the domain of plant microbiology.
2、The experimental design is meticulously crafted, leveraging a hydroponic system in conjunction with a synthetic microbial community to meticulously control experimental parameters, thereby ensuring the robustness and reliability of the findings.
3、The results have been meticulously analyzed, encompassing a spectrum of methodologies. This includes correlation analysis, corroborated by in vitro experiments, and soil treatment trials, all of which serve to substantiate the influence of dopamine on microbial communities.
Disadvantages:
1、The manuscript mentions that dopamine could promote the growth of Brachypodium distachyon under hydroponic conditions. Could the authors consider the specific pathways through which dopamine might exert this effect?
2、In the soil treatment experiments, dopamine significantly enriched for Actinobacteria. Could the authors provide more insights into the ecological significance of this phenomenon? Such as the role of Actinobacteria in soil ecosystems and how dopamine might modulate these roles?
3、The discussion suggests that dopamine may regulate rhizosphere microbial communities by affecting microbial gene expression and biofilm formation. Could the authors further elaborate on the molecular mechanisms of these processes?
4、The manuscript references dopamine but plant root exudates contain other metabolites as well. Could the authors discuss the interactions between these metabolites and dopamine, and how they collectively influence the structure and function of microbial communities?
5、In the hydroponic system, the complexity of root exudate-microbe interactions been considered? Are there control experiments to rule out the impact of the system itself on the results?
6、When performing association analyses, were other factors that might influence microbial community composition considered, such as plant genotype or environmental conditions?
7、The manuscript mentions correlations between dopamine and the abundance of specific bacterial groups. Could the authors further explore the potential mechanisms underlying these relationships?
8、The discussion should delve deeper into the significance of the findings, including how they connect with the existing body of knowledge and the potential implications for agriculture and ecology.
9、The description of experimental materials and methods in the paper is somewhat brief. It is recommended that the authors provide more detailed procedural steps and conditions in the supplementary materials to facilitate the reproduction of the experiments by other researchers.

Reviewer #2

(Remarks to the Author)
The manuscript submitted by Ding et al investigates the link between plant root exudate chemistry and the composition of the rhizosphere microbiome. Multiple experimental approaches have been undertaken, including: 1) Metabolomic profiling of root exudate composition from diverse Brachypodium lines; 2) Compositional profiling of the a 16-member bacterial SynCom inoculated onto those lines; 3) In vitro growth assays of individual bacteria supplemented with varying concentrations of dopamine; 4) Compositional analysis of a soil microbiome following incubation with dopamine. The main conclusion emphasised in the body text of the study is that plant roots exude large quantities of dopamine, which modulates the microbial community by enriching for certain taxa such as Actionbacteria.

The topic of the manuscript is important to the field, where there are ongoing efforts across a large number of laboratories aiming to define which plant metabolites exert the most influence on microbiome composition. In my opinion, the applied methods are appropriate tools to answer the questions being asked, and the untargeted metabolomics approach is technologically sophisticated and appears to be generally well-conducted.

From my reading of the manuscript, I can identify three main points that the authors wish to emphasise: 1) That Actinobacteria are enriched by the presence of dopamine; 2) That Brachypodium lines exhibit distinct root exudate composition and SynCom assembly; 3) That differential dopamine levels in the root exudates can (partially) explain the differential recruitment of bacterial strains into the rhizosphere.

As a reviewer, I am differentially convinced by each of these conclusions. Regarding the first conclusion (Dopamine favours certain strains, particularly Actinobacteria), I am strongly convinced that this conclusion is supported by the data reported in figures 3 & 4 and Supp Figures 13 & 14. To my knowledge, this is a novel finding for the field which could stimulate future research.

Regarding the second point (Brachypodium lines exhibit distinct exudate profiles & SynCom assembly), I am unconvinced that this conclusion is supported by the presented data. One of my major criticisms here, is that neither of the PCA in Figure 1b and the NMDS in Supp Figure 4 show the replicates, so it is hard to interpret whether these genotype-linked variations are robust and reproducible. From a graphic design perspective, I understand that the authors wanted to present fewer data points for ease of interpretation, but my opinion is that the authors must use these figures to present the variability in these measurements. This is particularly important for exudate analysis, which is notoriously prone to experimental variability. Therefore, I strongly advise that both the PCA in Figure 1b and the NMDS in Supp Figure 4 should be amended, to show every replicate data point (as was done in the PCA of Figure 4a).

Regarding the third point (Exuded plant root dopamine modulates bacterial colonisation), I am again unconvinced that this conclusion is supported by the presented data. There are two major reasons for this: 1) In Fig 2a, it is clear that there is an intense peak on the TICs of the first two chromatograms which coelutes with dopamine. However, from the presented data, I am unconvinced that dopamine is solely responsible for this peak. Therefore, I would like to see the complexity of the spectra which elute at this retention time, to know whether it is 'pure' dopamine, or a coeluting substance. 2) In the data presented in Figure 2b, it is clear that dopamine is depleted in the presence of the SynCom versus Axenic (presumably because it is consumed as a growth substrate). But I am unconvinced that the Brachypodium lines show reproducible differences in their dopamine levels. When I inspect the variance in the measurements of the green data series in Figure 2b, it appears that the SEM is roughly 75% of the mean. To me, such variance is indicative of a highly variable measurement which I would interpret with caution.

To summarise, the presented data have generally convinced me that Brachypodium plants exude dopamine, and also that dopamine can modulate microbiome composition. These are both novel and interesting findings. However, I am unconvinced whether Brachypodium lines show reproducible differences in their dopamine exudation, and therefore I do not see any evidence that dopamine explains any role in differential microbiome assembly between Brachypodium lines. As mentioned by the authors, there are no genetic knockouts of plant dopamine biosynthesis, which therefore makes it is difficult to design targeted experiments which dissect dopamine's role in microbiome assembly. But the correlative evidence present in this study is in my opinion insufficient to make a link between plant genetics, dopamine exudation, and microbiome composition.

Some minor points:
- In Supp Figure 9, I am a little bit confused by the differential units used to quantify the 13-C dopamine standard versus the endogenous dopamine in plant roots (mol/L versus g/gFW). This prevents me from judging whether their measured plant dopamine levels fall within the linear range of their calibration curve.
- In Supp Figure 10 Panel A, I suspect that the reported calibrations are being disproportionately driven by the single data point in the bottom-left corner of the scatter plots. Have the authors considered removing that single data point and then recomputing whether the correlation is still significant?
- In Supp Figure 10 Panel B, it seems that the growth response to exogenous dopamine follows an 'Inverse U-shape', whereby lower dopamine concentrations stimulate root growth, before higher concentrations seem to tend towards being toxic. Is this reflected in the figure caption 'Dopamine has a positive effect on plant growth'?
- Again in Supp Figure 10 Panel B, I cannot read the units of dopamine that were applied. (αM)?

Version 1:

Reviewer comments:

Reviewer #1

(Remarks to the Author)
Thanks for the efforts, the manuscript is better now, while I still have some comments for its improvement. In the Results section (lines 219-222), without additional data, this section overstates the mechanistic evidence for dopamine's growth-promoting effect by citing previous literature. However, no experimental evidence is provided in the current study to support these mechanistic explanations. This weakens the Results section, as it blends speculation with data presentation. The authors should: provide direct experimental data (e.g., hormone quantification, ROS assays, nitrogen assimilation measurements) to substantiate these proposed mechanisms.

While Actinobacteria are enriched upon dopamine treatment, the study does not provide any data to confirm that this enrichment translates to a beneficial functional outcome, such as enhanced plant growth promotion, pathogen suppression, or nutrient cycling.

In Fig 2b, under axenic conditions (without SynCom inoculation), dopamine levels vary widely across the ten B. distachyon lines. This large variability raises doubts about whether dopamine can be considered a key driver of the microbial shifts reported in Result 1. To strengthen the causal link, I strongly recommend the use of plant mutants impaired in dopamine biosynthesis or signaling, which would provide direct evidence of dopamine's role in shaping microbial community assembly. In addition, the number of biological replicates per line is only 2-3, and replicate variability is high. This substantially reduces the reliability of the conclusions. The authors should increase the number of replicates to improve statistical power and robustness of the findings.

In Fig 2c, the consistent and significant depletion of dopamine in the presence of the SynCom strongly suggests that dopamine is primarily utilized as a nutrient source by the microbial community. Furthermore, the marked reduction in variation of dopamine levels across the ten B. distachyon lines upon inoculation indicates that microbial metabolism is the dominant factor controlling dopamine abundance in the rhizosphere, overwhelming any underlying plant genotypic variation. It points to a model where dopamine is a common substrate that is consumed, and its level is a consequence of microbial activity rather than a cause of specific microbial enrichment.

The data presented in Fig. 4b suggest that the effects of dopamine and norepinephrine on the soil microbial community at the phylum level are strikingly similar. This observation challenges the manuscript's overarching narrative that specifically highlights dopamine. The authors dismiss the role of norepinephrine based on its trace level in B. distachyon roots compared to dopamine. However, this soil treatment experiment directly demonstrates that the soil microbiome is highly responsive to exogenous norepinephrine. This raises a critical question: could norepinephrine play a role just as important as dopamine? The current data cannot rule out this possibility. To substantiate the claim of dopamine's distinct importance, a parallel and comparative analysis is essential. Specifically, the authors should perform the same correlation analysis between norepinephrine (and other relevant exometabolites) and the SynCom member abundances that was conducted for dopamine. If dopamine shows uniquely strong and specific correlations with the bacterial, it would strengthen their case. Conversely, if norepinephrine shows similar correlative patterns, it would necessitate a significant revision of the discussion, framing the effect as a more general catecholamine phenomenon rather than a dopamine-specific story.

Specifically, the text states: 1) "Features unique to an individual line accounted for 18% of all features; for each individual line, the number varied between 6% to 12% of all features" (related to Supplementary Fig. 6b); and 2) "The proportion of increased features varied from 6% to 27%... while decreased features ranged from 2% to 18%" (related to Supplementary Fig. 7). However, Supplementary Fig. 6b is a simple pie chart without any numerical labels or a data table, making it impossible for a reader to confirm the stated 18% value or the per-line percentages. Similarly, Supplementary Fig. 7 consists of ten volcano plots, a format designed to show significance versus magnitude of change for individual features, but not to convey the overall proportion of features that were increased or decreased in a given line. The authors can modify these figures to directly visualize the quantitative summaries they describe in the text.

Supplementary figures use a mix of mol/L and g/gFW for dopamine quantification, which can be confusing. A consistent unit or a clear explanation for the difference should be provided in the figure legends.

The stress value for the NMDS plot (Supplementary Fig. 5) is not provided, which is necessary to interpret the reliability of the ordination.

The choice of Bd21-3 for detailed follow-up experiments (dopamine quantification in roots, plant growth assays) is not justified regarding its representativeness among the ten lines.

Dopamine was likely dissolved in a solvent (e.g., methanol, water) for soil application. A control with the solvent alone should have been included to rule out non-specific effects.

Reviewer #2

(Remarks to the Author)
I thank the authors for their thoughtful, respectful and comprehensive response to both reviewers. Having carefully read the authors' response to reviewers document, I now feel much more comfortable that the conclusions written in the manuscript text are supported by the measurements conducted.

I have only one small point of dissatisfaction: It involves Figure Panel 2b. For these data, I do not feel that the conclusions reported in the text (including the caption text) are representative of the measurements. I have various points of critique:

1) In the Caption Text of Figure 2, it states: "b shows that dopamine levels vary across ten B. distachyon lines" . In my interpretation, this is factually incorrect - according to the ANOVA-Post-Hoc-Test, the dopamine levels in all of the axenic Brachypodium lines are encompassed by the letter "a", indicating that dopamine levels do NOT reproducibly vary from line to line.

2) In the body text line 181, it states: "we observed natural variation in the dopamine levels in root exudates of the ten B. distachyon lines" . Again, I don't feel that this conclusion is supported by the data, which is indicative of technical variation rather than biological.

3) In the body text lines 185-186, it states that: "dopamine levels in the presence of the SynCom were generally consistent across replicates and exhibited significant differences between certain plant lines (Fig. 2b) . In my interpretation, this statement is in a 'grey zone', whereby the statement is factually true, but doesn't represent the major findings of the experiment. To clarify, root exudates are a notoriously variable sample type. It is to be expected that these measurements of dopamine levels in root exudates in an n=3 experiment will be highly variable. But in my interpretation, the primary finding is of this experiment is actually presented in Figure 2c, where the authors convincingly show that SynCom presence results in lower dopamine levels versus axenic, presumably because some of the strains have consumed the plant-exuded dopamine as a metabolic substrate. In contrast, I am not convinced that the authors have reproducibly measured biologically meaningful line-to-line dopamine variation. I accept that the chosen test has resulted in certain SynCom-treated lines being annotated with either 'a' or 'b', but upon closer inspection, these effect sizes are extremely small and probably biologically insignificant.

4) About the choice of statistics for Figure Panel 2B, I am not sure whether the choice of tests is appropriate. The caption text states: "statistical analyses were conducted separately for dopamine levels under axenic conditions and in the presence of the SynCom. Different letters (a-b) indicate significant differences as determined by Welch's ANOVA followed by Dunnett's T3 multiple comparison test ($P < 0.05$)." If the authors really want to present this conclusion in the published manuscript, can they please justify the following: (a) Why were axenic- and SynCom- treated split and not bundled? (b) Why was Welch's ANOVA used instead of an alternative test? (c) Why was Dunnett's test used instead of an alternative test?

Again, I thank the authors for their extensive modifications to the revised manuscript, which overall have reassured me that their conclusions are indeed supported by their measurements.

Version 2:

Reviewer comments:

Reviewer #1

(Remarks to the Author)
Thanks for the efforts, the current version is good and satisfactory, there is no more additional comments.

Reviewer #2

(Remarks to the Author)
I thank the authors for their extensive comments to both reviewers, and the effort taken to conduct the additional experiment measuring root exudate LC-MS/MS profiles across Brachypodium genotypes.

I have only one single comment: Can the authors please incorporate some qualifying text, reflecting the robustness/plasticity of the link between plant genotype versus dopamine exudation?

To explain, I have re-inspected the data in Fig 2b, as well as the newly acquired data in Supplementary Fig. 11. My conclusion from these two datasets is that root dopamine exudation is NOT a heritable trait which can be robustly linked to plant genotype. I fully accept that the data in Supplementary Fig 11 do indeed show lower variance of dopamine exudation within the 6x replicates of each Brachypodium line, when compared to the previous n=3 data. However, a brief comparison of the two datasets shows that the genotypes now have a dramatically different rank order of dopamine exudation strength. This is exemplified by Bd 2-3, which seems to have jumped from the weakest dopamine exuder in the initial experiment to the strongest exuder in the follow-up experiment. To me, this low replicability indicates that dopamine exudation strength is not robustly linked to Brachypodium genotype.

Potentially dopamine exudation is a plastic phenotypic trait. Potentially there are complex GxE interactions which shape dopamine exudation levels. I would appreciate some rewording of the text to clarify this to the reader.

In particular, the newly included line 186 "which supported reproducible differences in dopamine abundance among lines" is for me too strongly worded. I recommend that the authors rephrase the text from lines 176-187. In my view, no strong conclusions about genotypic variation in dopamine exudation can be made from this set of measurements.

**Open Access** This Peer Review File is licensed under a Creative Commons Attribution 4.0 International License, which permits use, sharing, adaptation, distribution and reproduction in any medium or format, as long as you give appropriate credit to the original author(s) and the source, provide a link to the Creative Commons license, and indicate if changes were made.
In cases where reviewers are anonymous, credit should be given to 'Anonymous Referee' and the source.
The images or other third party material in this Peer Review File are included in the article's Creative Commons license, unless indicated otherwise in a credit line to the material. If material is not included in the article's Creative Commons license and your intended use is not permitted by statutory regulation or exceeds the permitted use, you will need to obtain permission directly from the copyright holder.
To view a copy of this license, visit https://creativecommons.org/licenses/by/4.0/

# Responses to Reviewers' Comments

We thank both reviewers for their thoughtful evaluation of our manuscript entitled "**Insights into the role of dopamine in rhizosphere microbiome assembly**" (NCOMMS-24-49252). We are grateful for the positive feedback on our manuscript as well as the additional constructive comments provided. In response, we have further revised the manuscript as detailed in the point-by-point replies below. Please note that the line numbers cited correspond to the revised version, and all edits in the manuscript are highlighted in blue.

REVIEWER COMMENTS
Reviewer #1 (Remarks to the Author):

This study delves into the role of dopamine in the assembly of the plant rhizosphere microbiome. It was discovered that dopamine levels in root exudates vary significantly across different Brachypodium distachyon lines and were notably associated with the abundance of six members of a synthetic community (SynCom). Further in vitro growth studies had demonstrated that dopamine significantly influences the growth of these six bacterial isolates. Soil treatment experiments had shown that the direct application of dopamine enriches for Actinobacteria, aligning with the SynCom-dopamine correlations and isolate growth outcomes. The research underscores the selective impact of dopamine on rhizosphere microbial communities, offering potential for precision microbiome management.

Advantages:
1、The study introduces a pioneering and compelling investigation into the role of dopamine in the assembly of the plant rhizosphere microbiome, thereby opening new frontiers for research within the domain of plant microbiology.
2、The experimental design is meticulously crafted, leveraging a hydroponic system in conjunction with a synthetic microbial community to meticulously control experimental parameters, thereby ensuring the robustness and reliability of the findings.
3、The results have been meticulously analyzed, encompassing a spectrum of methodologies. This includes correlation analysis, corroborated by in vitro experiments, and soil treatment trials, all of which serve to substantiate the influence of dopamine on microbial communities.

We sincerely thank the reviewer for their thoughtful evaluation of our manuscript and for the positive feedback and recognition of our study's contribution to understanding dopamine's role in rhizosphere microbiome assembly.

Disadvantages:
1、The manuscript mentions that dopamine could promote the growth of *Brachypodium distachyon* under hydroponic conditions. Could the authors consider the specific pathways through which dopamine might exert this effect?

Great suggestion. Dopamine's role in promoting *Brachypodium distachyon* growth under hydroponic conditions could be attributed to several potential mechanisms. One possibility is its interaction with plant hormone pathways, such as auxin signaling, which are crucial for root and

shoot development. Dopamine has been reported to influence auxin transport and homeostasis, potentially enhancing root architecture and nutrient uptake (Liu et al., 2020). Additionally, dopamine may act as an antioxidant, helping to regulate reactive oxygen species (ROS) levels (Cao et al., 2020), thereby improving stress resilience and overall growth. Another plausible pathway involves dopamine's effect on nitrogen metabolism, as it has been suggested to enhance nitrate assimilation and amino acid biosynthesis (Kulma & Szopa, 2007), both of which are essential for biomass accumulation. Further studies, such as transcriptomic or metabolomic analyses, could help elucidate the specific molecular pathways through which dopamine enhances growth in *Brachypodium distachyon*.

We added the following text in the revised manuscript (Lines 219-222):

"Dopamine's growth-promoting effect may stem from its interaction with plant hormone pathways, its role as an antioxidant in regulating ROS (reactive oxygen species) levels, and its enhancement of nitrogen metabolism[26,30,31]."

**References:**

26. Liu, Q., Gao, T., Liu, W., Liu, Y., Zhao, Y., Liu, Y., Li, W., Ding, K., Ma, F., & Li, C. (2020). Functions of dopamine in plants: a review. *Plant Signaling & Behavior, 15*(12), 1827782. https://doi.org/10.1080/15592324.2020.1827782
30. Gao, T., Zhang, Z., Liu, X., Wu, Q., Chen, Q., Liu, Q., Van Nocker, S., Ma, F., & Li, C. (2020). Physiological and transcriptome analyses of the effects of exogenous dopamine on drought tolerance in apple. *Plant Physiology and Biochemistry, 148*, 260–272.
31. Kulma, A., & Szopa, J. (2007). Catecholamines are active compounds in plants. *Plant Science, 172*(3), 433–440. https://doi.org/10.1016/j.plantsci.2006.10.011

2、In the soil treatment experiments, dopamine significantly enriched for Actinobacteria. Could the authors provide more insights into the ecological significance of this phenomenon? Such as the role of Actinobacteria in soil ecosystems and how dopamine might modulate these roles?

We appreciate the reviewer's insightful question regarding the ecological significance of dopamine-mediated Actinobacteria enrichment.

We added the following text in the discussion (Lines 327-333):

"Actinobacteria play essential roles in soil ecosystems, contributing to organic matter decomposition, nutrient cycling, and plant growth promotion[42]. Many Actinobacteria species, such as Streptomyces, produce bioactive compounds that enhance plant disease resistance and improve soil health. Additionally, Actinobacteria are known to be enriched under drought conditions, suggesting a potential role in stress adaptation[42,43]. Therefore, their observed enrichment in dopamine-treated soil may provide a mechanism for increasing these beneficial effects."

**References:**

42. Barka, E. A., et al. (2016). Taxonomy, physiology, and natural products of Actinobacteria. *Microbiol. Mol. Biol. Rev*., 80(1), 1-43.

43. Naylor, D., et al. (2017). Drought and host selection influence bacterial community dynamics in the grass root microbiome. *ISME J.,* 11(11), 2691-2704.

3、The discussion suggests that dopamine may regulate rhizosphere microbial communities by affecting microbial gene expression and biofilm formation. Could the authors further elaborate on the molecular mechanisms of these processes?

We appreciate the reviewer's suggestion to elaborate on the molecular mechanisms by which dopamine regulates rhizosphere microbial communities, particularly through its effects on microbial gene expression and biofilm formation.

We have added the following text to the discussion section (Lines 335-344):

"Dopamine has been shown to modulate quorum sensing pathways in bacteria, which regulate biofilm formation and stress responses[44]. This suggests that dopamine could influence microbial community assembly by altering microbial gene expression and interspecies interactions. Most Actinobacteria are known to produce extracellular polysaccharides and secondary metabolites that contribute to biofilm development and microbial resilience[42]. Additionally, the ability of dopamine to influence Actinobacteria abundance may stem from its role as a potential carbon and nitrogen source and/or its redox properties that affect microbial competition and survival in the rhizosphere[41]. Further studies are needed to determine the precise bacterial pathways regulated by dopamine and their functional consequences in the rhizosphere."

We believe this addition strengthens the manuscript by addressing the molecular basis of dopamine's impact on rhizosphere microbial communities. We thank the reviewer for this insightful suggestion.

**References:**
41. Zhalnina, K., et al. (2018). Dynamic root exudate chemistry and microbial substrate preferences drive patterns in rhizosphere microbial community assembly. *Nature Microbiology*, 3, 470–480.
42. Barka, E. A., Vatsa, P., Sanchez, L., Gaveau-Vaillant, N., Jacquard, C., Meier-Kolthoff, J. P., ... & Clément, C. (2016). Taxonomy, physiology, and natural products of Actinobacteria. *Microbiology and Molecular Biology Reviews*, 80(1), 1–43.
44. Xiang, S. L., Xu, K. Z., Yin, L. J., Rao, Y., Wang, B., & Jia, A. Q. (2024). Dopamine, an exogenous quorum sensing signaling molecule or a modulating factor in Pseudomonas aeruginosa? *Biofilm*, 8, 100208. https://doi.org/10.1016/j.bioflm.2024.100208

4、The manuscript references dopamine but plant root exudates contain other metabolites as well. Could the authors discuss the interactions between these metabolites and dopamine, and how they collectively influence the structure and function of microbial communities?

Good point. We have expanded the discussion to address how dopamine functions within the broader chemical context of root exudates (Lines 356-370).

"Plant root exudates are complex mixtures of metabolites that collectively influence rhizosphere microbial communities. While our study focuses on dopamine, its interactions with other exuded compounds may play a crucial role in shaping microbial recruitment and activity. Flavonoids, phenolic acids, organic acids, and amino acids are among the key metabolites found in root exudates, each contributing to microbial signaling, gene regulation, and metabolic adaptation[8]. These metabolites can modulate microbial responses to dopamine by affecting its bioavailability, degradation, or uptake pathways[40]. For example, phenolic compounds with redox activity may interact with dopamine, altering its chemical stability and microbial utilization. Additionally, organic acids secreted by roots can influence soil pH and metal availability, indirectly impacting microbial community composition and dopamine-mediated effects[41]. By considering dopamine in the broader context of root exudate chemistry, we propose that its selective enrichment of specific microbial taxa, such as Actinobacteria, may be influenced by co-exuded metabolites that act synergistically or antagonistically. Future studies are needed to disentangle these complex metabolite-microbe interactions and uncover how plants fine-tune their rhizosphere microbiomes through diverse chemical cues."

**References:**

8. Sasse, J., Martinoia, E., & Northen, T. (2018). Feed your friends: Do plant exudates shape the root microbiome? *Trends in Plant Science*, 23(1), 25-41.
40. Voges, M. J. E. E., Bai, Y., Schulze-Lefert, P., & Sattely, E. S. (2019). Plant-derived coumarins shape the composition of an Arabidopsis synthetic root microbiome. *Proceedings of the National Academy of Sciences*, 116(25), 12558-12565.
41. Zhalnina, K., Louie, K. B., Hao, Z., Mansoori, N., da Rocha, U. N., Shi, S., Cho, H., Karaoz, U., Loqué, D., Bowen, B. P., Firestone, M. K., Northen, T. R., & Brodie, E. L. (2018). Dynamic root exudate chemistry and microbial substrate preferences drive patterns in rhizosphere microbial community assembly. *Nature Microbiology*, 3, 470–480.

5、In the hydroponic system, the complexity of root exudate-microbe interactions been considered? Are there control experiments to rule out the impact of the system itself on the results?

We appreciate the reviewer's insightful question regarding the complexity of root exudate-microbe interactions in the hydroponic system and the potential influence of the system itself on our results. We have modified the text as shown below to comment on this limitation of our study. We also think that by studying the effect of dopamine in soils, this limitation is partially mitigated, but we agree that additional studies are needed.

We added one sentence in Lines 299-300: "While hydroponic systems provide a controlled environment, they do not fully replicate soil complexity."

6、When performing association analyses, were other factors that might influence microbial community composition considered, such as plant genotype or environmental conditions?

We appreciate the reviewer's important question. In this study, our results support that plant genotype plays a role in shaping both exudate metabolic profiles and microbial community composition. The goal of the correlation analysis is to link plant metabolites in different lines to

community composition, so plant genotype is not a confounding factor. To minimize environmental variability, all experiments were conducted under controlled hydroponic conditions, ensuring consistent nutrient availability, temperature, and light exposure across replicates.

We added the following text in the manuscript to address this in Lines 296-304:

"The distinct metabolite profiles and SynCom compositions observed across these lines suggest that plant genotype influences both exudate chemistry and microbial recruitment. Additionally, to minimize environmental variability, we conducted all experiments under controlled hydroponic conditions, ensuring uniform nutrient availability, temperature, and light exposure. While hydroponic systems provide a controlled environment, they do not fully replicate soil complexity. To address this, we complemented our findings with soil treatment experiments, which further validated dopamine's role in shaping microbial communities. These complementary approaches strengthen our conclusions by demonstrating that dopamine-mediated microbial shifts are observed across different conditions."

7、The manuscript mentions correlations between dopamine and the abundance of specific bacterial groups. Could the authors further explore the potential mechanisms underlying these relationships?

Good point. To address this, we expanded the discussion to include potential pathways through which dopamine influences microbial communities as addressed above in our response to Comment 3.

8、The discussion should delve deeper into the significance of the findings, including how they connect with the existing body of knowledge and the potential implications for agriculture and ecology.

We appreciate the reviewer's suggestion to expand the discussion on the significance of our findings. A new paragraph has been added to the Discussion section to highlight these points (Lines 372-382).

"Our findings provide new insights into the potential role of dopamine as a modulator of rhizosphere microbial communities, reinforcing the growing recognition of plant-derived secondary metabolites in shaping soil microbiomes[41]. By demonstrating that dopamine correlates with the abundance of specific bacterial groups, we expand on previous studies showing that root exudates serve as chemical signals influencing microbial assembly. These interactions have significant implications for agriculture and ecology, including optimizing plant-derived metabolites to promote beneficial microbes, enhance nutrient cycling, and improve plant stress tolerance[30]. Understanding the ecological roles of dopamine in the rhizosphere may also contribute to the development of bioinoculants or microbiome engineering approaches for improving crop resilience under climate change conditions. Although our findings suggest that dopamine plays a role in modulating rhizosphere microbial communities, further experimental validation is needed to confirm its functional effects on microbial physiology and community dynamics, particularly under natural field conditions."

**References:**

30. Gao, T., Zhang, Z., Liu, X., Wu, Q., Chen, Q., Liu, Q., Nocker, S. V., Ma, F., & Li, C. (2020). Physiological and transcriptome analyses of the effects of exogenous dopamine on drought tolerance in apple. *Plant Physiology and Biochemistry, 148*, 260–272. https://doi.org/10.1016/j.plaphy.2020.01.022

41. Zhalnina, K., et al. (2018). Dynamic root exudate chemistry and microbial substrate preferences drive patterns in rhizosphere microbial community assembly. *Nature Microbiology, 3*, 470–480.

9、The description of experimental materials and methods in the paper is somewhat brief. It is recommended that the authors provide more detailed procedural steps and conditions in the supplementary materials to facilitate the reproduction of the experiments by other researchers.

Thank you for the suggestion. We recognize the importance of providing detailed experimental materials and methods to ensure reproducibility. In the revised manuscript, we provided more detailed procedural steps and conditions in the Materials and Methods section.

**We added the following text in Lines 457-469:**

**"Dopamine Identification using LC-MS/MS**

Dopamine was identified in *B. distachyon* root exudates and tissues using high-resolution LC-MS/MS. Sample extracts were separated using a HILIC column and then analyzed on a Thermo Q Exactive Orbitrap mass spectrometer (See Supplementary Table 6 for details on the LC-MS/MS conditions). The in-source fragment of dopamine (*m*/*z* 137.0597, $[M + H - NH_3]^+$) was observed to be one of the major MS1 ions and was used for relative quantification. The dopamine precursor ion at *m*/*z* 154.0862 ($[M + H]^+$) was also detected but was consistently less abundant in the MS1 scans. Dopamine identification was confirmed based on matching the exact mass, retention time, and MS/MS fragmentation pattern to an authentic dopamine standard (Sigma-Aldrich) analyzed under identical conditions. Additional confirmation was provided by spiking [ring-$^{13}C_6$]-labeled dopamine (Cambridge Isotope Laboratories) into the extraction buffer as an internal standard. Base peak chromatograms and extracted ion chromatograms were prepared using the FreeStyle software (FreeStyle™ 1.5, Thermo Fisher Scientific)."

**We added the following text in Lines 480-484:**

"Non-metric multidimensional scaling (NMDS) analysis was performed to visualize differences in exudate profiles of ten different *B. distachyon* lines in the absence and presence of the SynCom. NMDS analysis was conducted using the *metaMDS* function from the *vegan* R package, and the resulting ordination plot was generated using *ggplot2* in R."

**We added the following text in Lines 528-532:**

"Pairwise comparisons were conducted using the contrast function across all combinations of treatments. Comparisons with an adjusted *P*-value greater than 0.05 were discarded. Hierarchical clustering was performed using the Seaborn package in Python with default parameters. Notably,

Bd21 16S rRNA data is limited to a single replicate, whereas other lines had two or three replicates available."

**We added the following text in Lines 541-542:**

"This approach enables accurate quantification of endogenous dopamine within a dynamic range of 0.38 to 6.13 mg/g fresh tissues."

**We added the following text in Lines 558-559:**

"The plant endogenous dopamine concentrations were converted to milligrams of $^{12}$C-dopamine per gram fresh weight."

**We added the following text in Lines 574-576:**

"Group differences were assessed using one-way ANOVA when variances were homogeneous and Welch's ANOVA when variances were unequal, followed by Tukey's or Dunnett's T3 post hoc tests, respectively."

Reviewer #2 (Remarks to the Author):

The manuscript submitted by Ding et al investigates the link between plant root exudate chemistry and the composition of the rhizosphere microbiome. Multiple experimental approaches have been undertaken, including: 1) Metabolomic profiling of root exudate composition from diverse Brachypodium lines; 2) Compositional profiling of the a 16-member bacterial SynCom inoculated onto those lines; 3) In vitro growth assays of individual bacteria supplemented with varying concentrations of dopamine; 4) Compositional analysis of a soil microbiome following incubation with dopamine. The main conclusion emphasised in the body text of the study is that plant roots exude large quantities of dopamine, which modulates the microbial community by enriching for certain taxa such as Actionbacteria.

The topic of the manuscript is important to the field, where there are ongoing efforts across a large number of laboratories aiming to define which plant metabolites exert the most influence on microbiome composition. In my opinion, the applied methods are appropriate tools to answer the questions being asked, and the untargeted metabolomics approach is technologically sophisticated and appears to be generally well-conducted.

We sincerely thank the reviewer for their effort in reviewing our manuscript and for their thoughtful comments and positive evaluation of our manuscript.

From my reading of the manuscript, I can identify three main points that the authors wish to emphasise: 1) That Actinobacteria are enriched by the presence of dopamine; 2) That Brachypodium lines exhibit distinct root exudate composition and SynCom assembly; 3) That differential dopamine levels in the root exudates can (partially) explain the differential recruitment of bacterial strains into the rhizosphere.

As a reviewer, I am differentially convinced by each of these conclusions. Regarding the first conclusion (Dopamine favours certain strains, particularly Actinobacteria), I am strongly convinced that this conclusion is supported by the data reported in figures 3 & 4 and Supp Figures 13 & 14. To my knowledge, this is a novel finding for the field which could stimulate future research.

Regarding the second point (Brachypodium lines exhibit distinct exudate profiles & SynCom assembly), I am unconvinced that this conclusion is supported by the presented data. One of my major criticisms here, is that neither of the PCA in Figure 1b and the NMDS in Supp Figure 4 show the replicates, so it is hard to interpret whether these genotype-linked variations are robust and reproducible. From a graphic design perspective, I understand that the authors wanted to present fewer data points for ease of interpretation, but my opinion is that the authors must use these figures to present the variability in these measurements. This is particularly important for exudate analysis, which is notoriously prone to experimental variability. Therefore, I strongly advise that both the PCA in Figure 1b and the NMDS in Supp Figure 4 should be amended, to show every replicate data point (as was done in the PCA of Figure 4a).

We thank the reviewer for their thoughtful and constructive comments. We have revised both Fig. 1b and Supplementary Fig. 5 (in the revised manuscript) to include all replicate data points. These updated figures now more accurately reflect within-line variation in SynCom composition and exudate metabolite profiles. As shown in the revised Fig. 1b, several *Brachypodium* lines, such as Adi9, ABR6, and Bd3-1, exhibit clear and consistent separation on the PCA ordination plot. Additionally, statistical analysis reveals that SynCom member abundances differ significantly between certain lines (new Supplementary Fig. 4).

Similarly, in the revised Supplementary Fig. 5, we also observed that certain lines under axenic conditions, such as Adi9, ABR6, and Bd3-1, exhibit distinct exudate profiles, which are also supported by data present in Supplementary Fig. 6, 7, and 8.

While some lines, such as Bd21-3 and BdTR10h in Fig. 1b, display more dispersed distributions, this likely reflects higher within-line variation and reinforces the importance of including replicates to capture biological variability. Overall, our data support the conclusion that host genotype contributes to shaping both exudate chemistry and rhizosphere microbiota, consistent with previous findings in other plant species (Badri et al., 2013; Sasse et al., 2018; Zhalnina et al., 2018; Deng et al., 2021).

The following are the revised Figure 1b and Supplementary Figure 5 with replicates, as well as new Supplementary Figure 4.

Original Fig. 1b

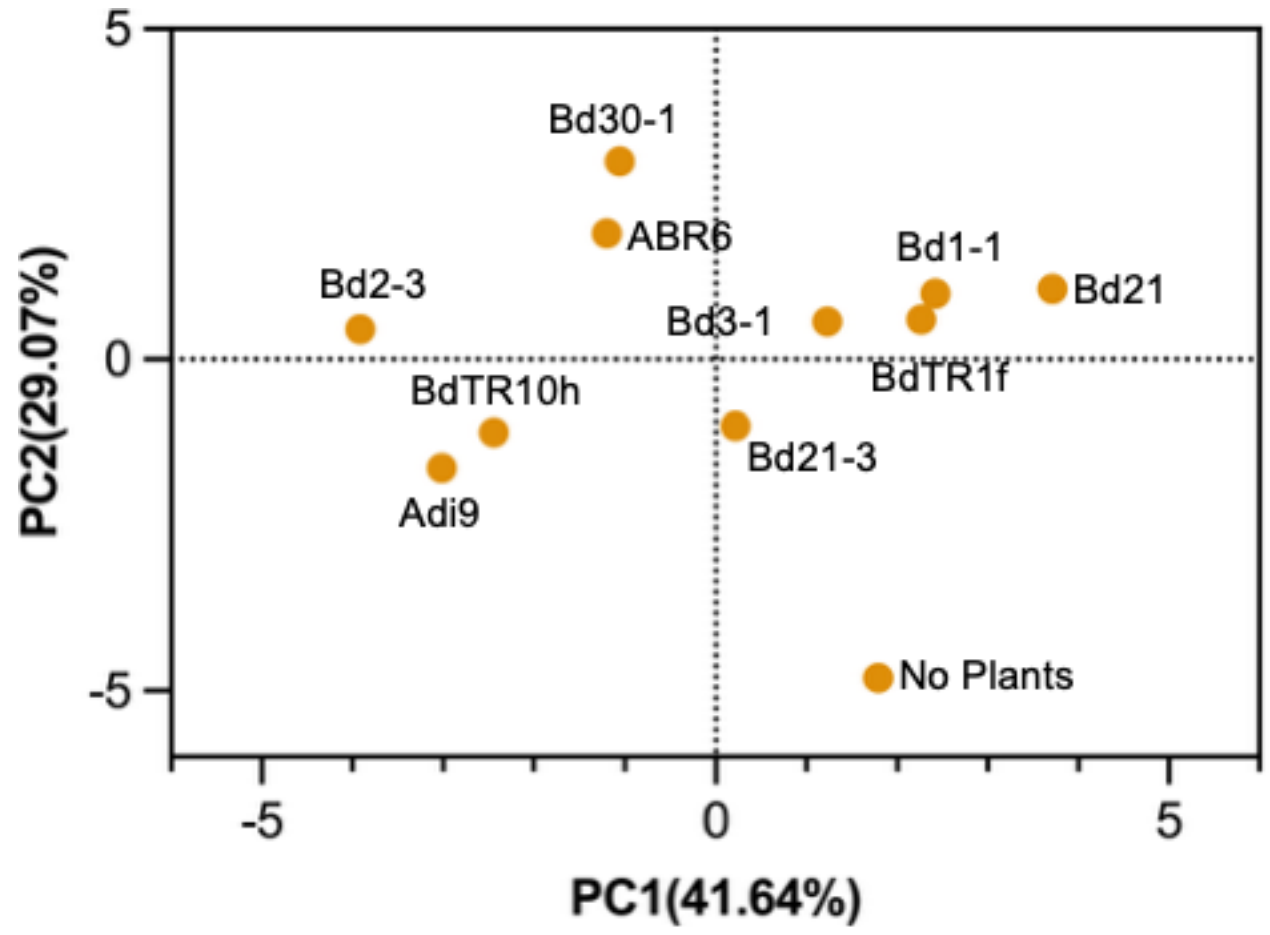


Revised Fig. 1b

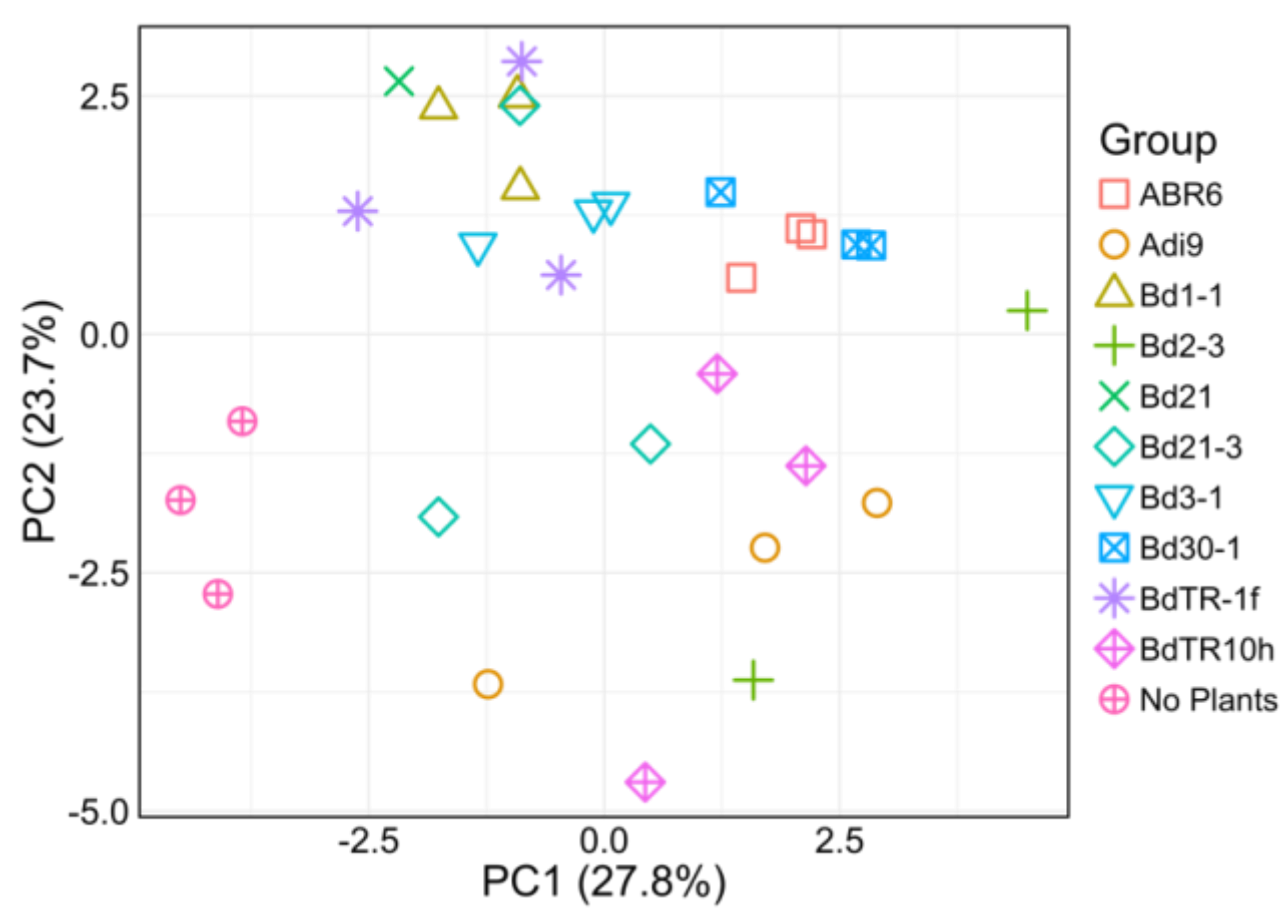

Original Supplementary Fig. 5

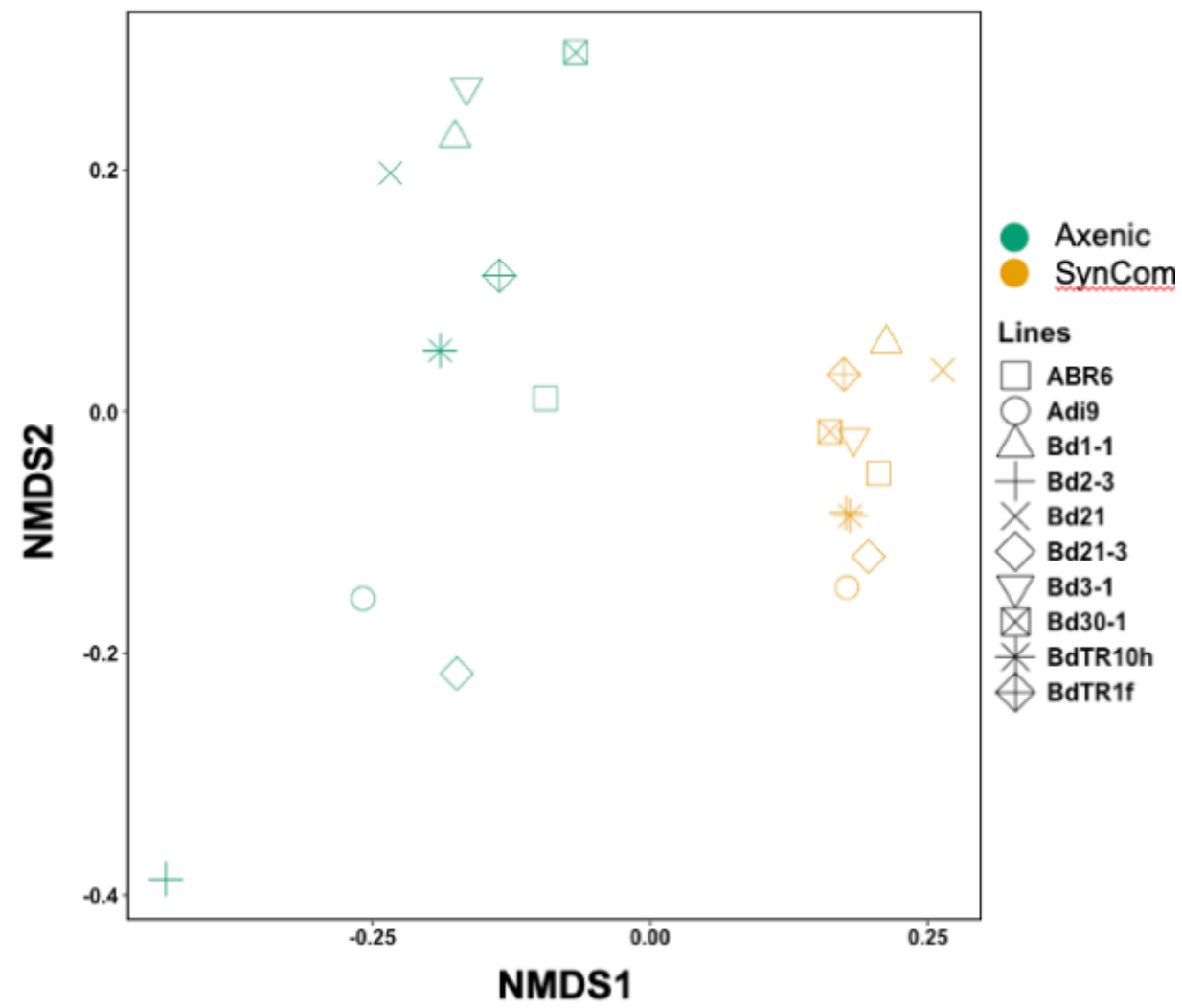


Revised Supplementary Fig. 5

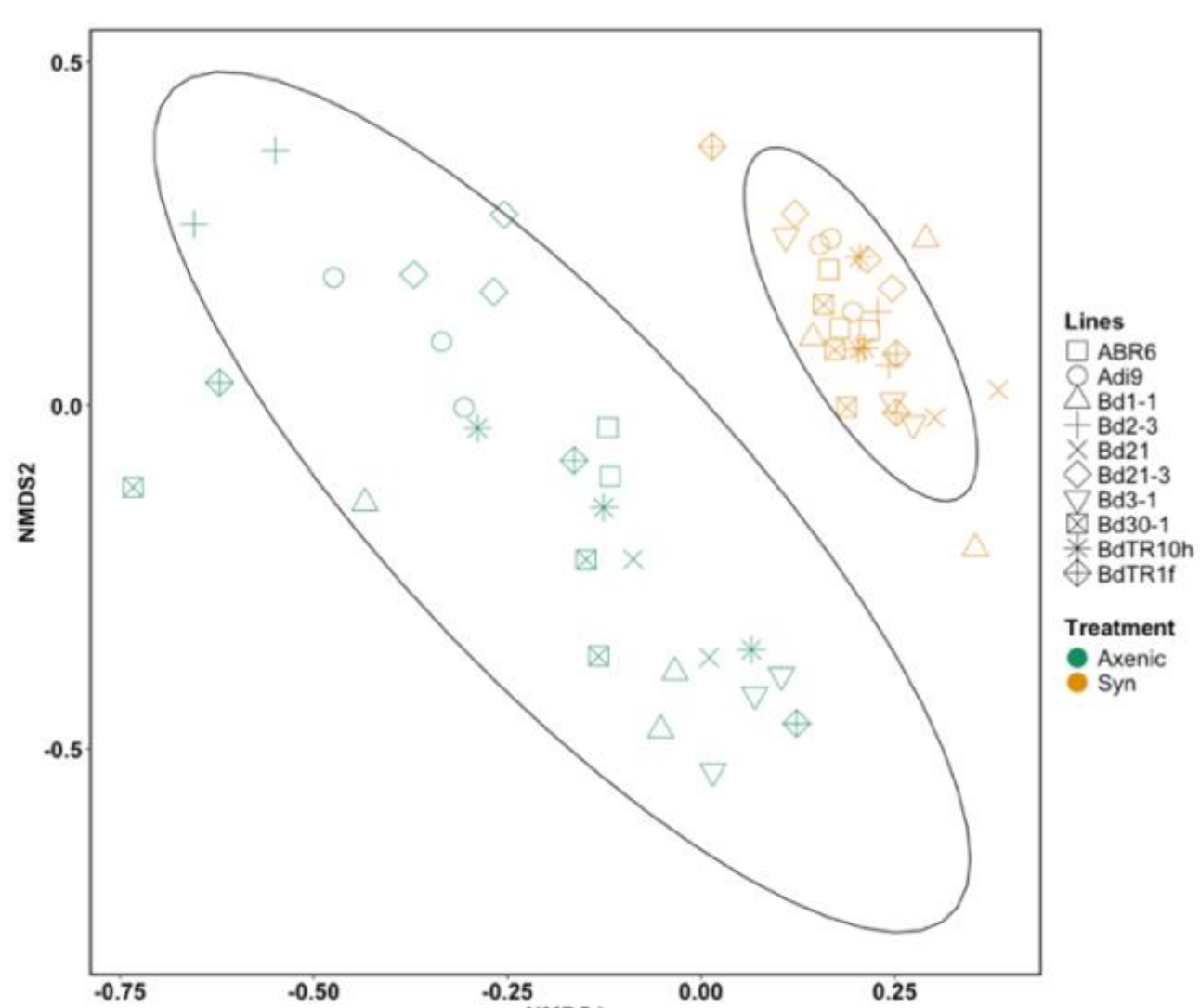

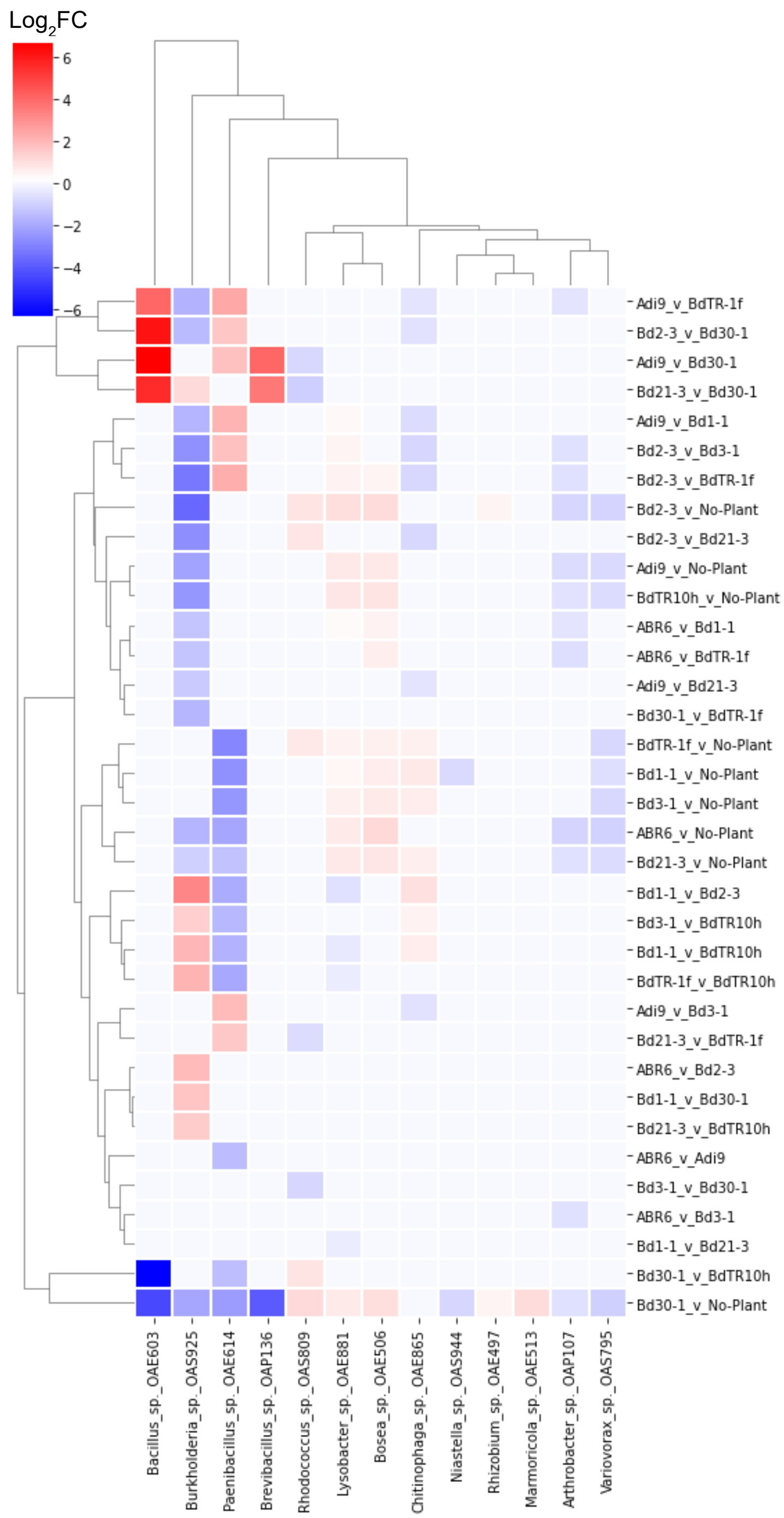


**Supplementary Fig. 4. Pairwise comparisons of SynCom member abundances across *B. distachyon* lines.** Deseq2 was used to compare SynCom member abundances in the spent media of *Brachypodium* lines and No Plant (Ctr). Log$_2$ fold change (Log$_2$FC) values of SynCom members that were significantly different (adjusted *P*-value <0.05) were then clustered (non-significant Log$_2$ FCs were set to 0 for clarity). Red, >0, indicates that the isolate was significantly higher in the second condition listed whereas blue, <0, indicates that the isolate was significantly higher in the first condition listed.

**References:**
Badri, D. V., Zolla, G., Bakker, M. G., Manter, D. K., & Vivanco, J. M. (2013). Genotype-specific exudation shapes rhizosphere microbiome assembly in *Arabidopsis*. *Plant Physiology*, 163, 1877–1890.
Sasse, J., Martinoia, E., & Northen, T. (2018). Feed your friends: Do plant exudates shape the root microbiome? *Trends in Plant Science*, 23, 25–41.
Zhalnina, K., Louie, K. B., Hao, Z., Mansoori, N., da Rocha, U. N., Shi, S., ... & Firestone, M. K. (2018). Dynamic root exudate chemistry and microbial substrate preferences drive patterns in rhizosphere microbial community assembly. *Nature Microbiology*, 3, 470–480.
Deng, S., Wipf, H. M.-L., Pierroz, G., Raizada, M. N., & Dangl, J. L. (2021). Genome wide association study reveals plant loci controlling heritability of the rhizosphere microbiome. *ISME Journal*, 15, 3181–3194.

Regarding the third point (Exuded plant root dopamine modulates bacterial colonisation), I am again unconvinced that this conclusion is supported by the presented data. There are two major reasons for this:
1) In Fig 2a, it is clear that there is an intense peak on the TICs of the first two chromatograms which coelutes with dopamine. However, from the presented data, I am unconvinced that dopamine is solely responsible for this peak. Therefore, I would like to see the complexity of the spectra which elute at this retention time to know whether it is 'pure' dopamine or a coeluting substance.

Thank you for pointing this out. You are correct that the TIC contains signals from many ions (see below). However, the $[M + H - NH_3]^+$ ion of dopamine (*m/z* 137.0547-137.0647) is one of the most abundant ions. To address this, we have revised the Fig. 2a (see below) to display base peak chromatograms instead of TICs along with the dopamine EICs and MS/MS in Supplementary Fig. 9.

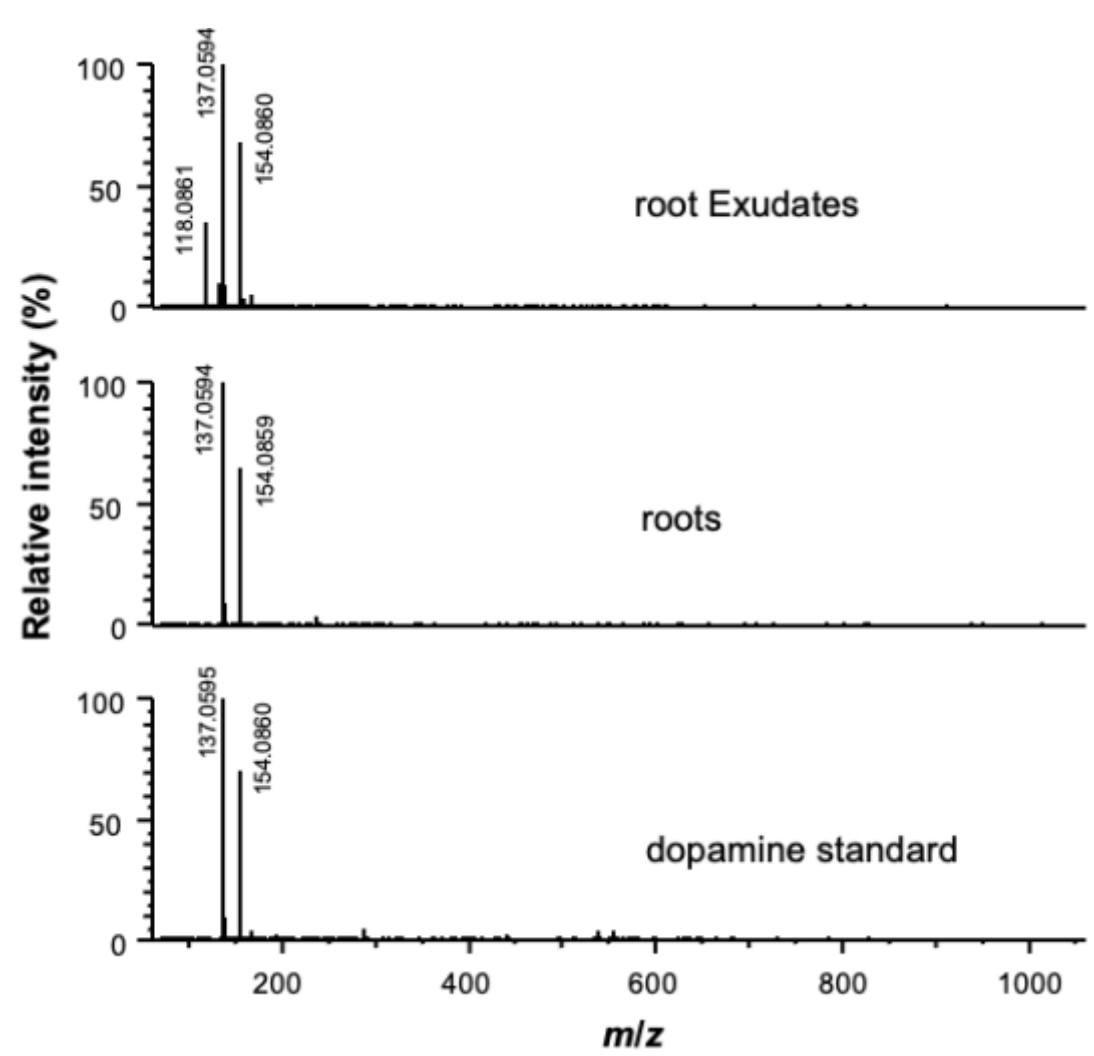


The above figure shows the full MS1 spectra at the retention time of dopamine's highest peak, corresponding to the two TICs (root exudates and roots) and the EIC (the dopamine standard) presented in the original Fig. 2a. Because the revised Fig. 2a now displays base peak chromatograms, the full MS1 spectra are not included in the revised manuscript.

We added the following text in the Methods section (Lines 457-469):

**"Dopamine Identification using LC-MS/MS**

Dopamine was identified in *B. distachyon* root exudates and tissues using high-resolution LC-MS/MS. Sample extracts were separated using a HILIC column and then analyzed on a Thermo Q Exactive Orbitrap mass spectrometer (See Supplementary Table 6 for details on the LC-MS/MS conditions). The in-source fragment of dopamine (*m/z* 137.0597, $[M + H - NH_3]^+$)[56] was observed to be one of the major MS1 ions and was used for relative quantification. The dopamine precursor ion at *m/z* 154.0862 ($[M + H]^+$) was also detected but was consistently less abundant in the MS1 scans. Dopamine identification was confirmed based on matching the exact mass, retention time, and MS/MS fragmentation pattern to an authentic dopamine standard (Sigma-Aldrich) analyzed under identical conditions. Additional confirmation was provided by spiking [ring-$^{13}C_6$]-labeled dopamine (Cambridge Isotope Laboratories) into the extraction buffer as an internal standard. Base peak chromatograms and extracted ion chromatograms were prepared using the FreeStyle software (FreeStyle™ 1.5, Thermo Fisher Scientific)."

**Reference:**

56. Asakawa D, Sugiyama E, Mizuno H, Todoroki K. Study of Substituted Phenethylamine Fragmentation Induced by Electrospray Ionization Mass Spectrometry and Its Application for Highly Sensitive Analysis of Neurotransmitters in Biological Samples. *J Am Soc Mass Spectrom* 32, 2144-2152 (2021).

Original Fig. 2a,

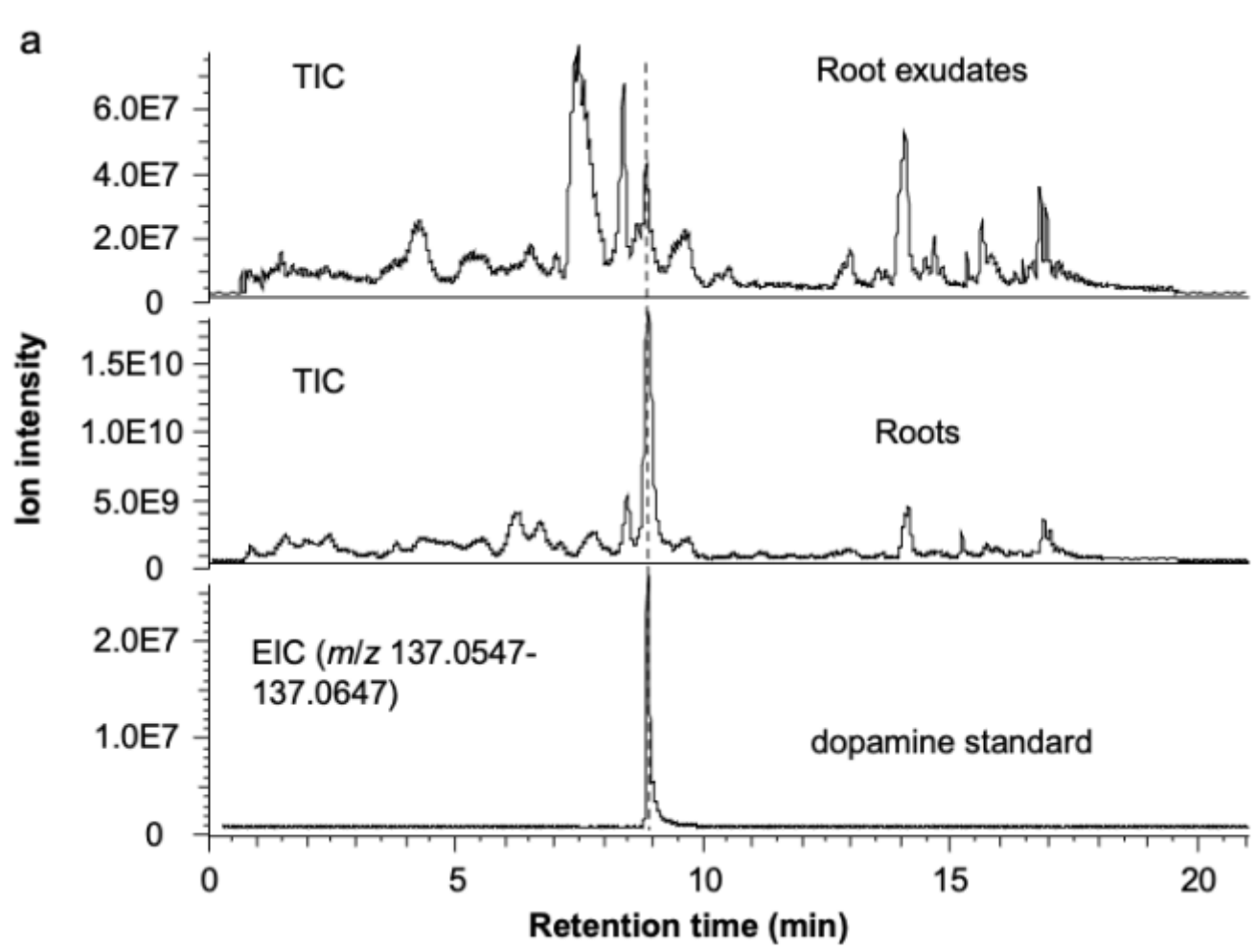


Revised Fig. 2a,

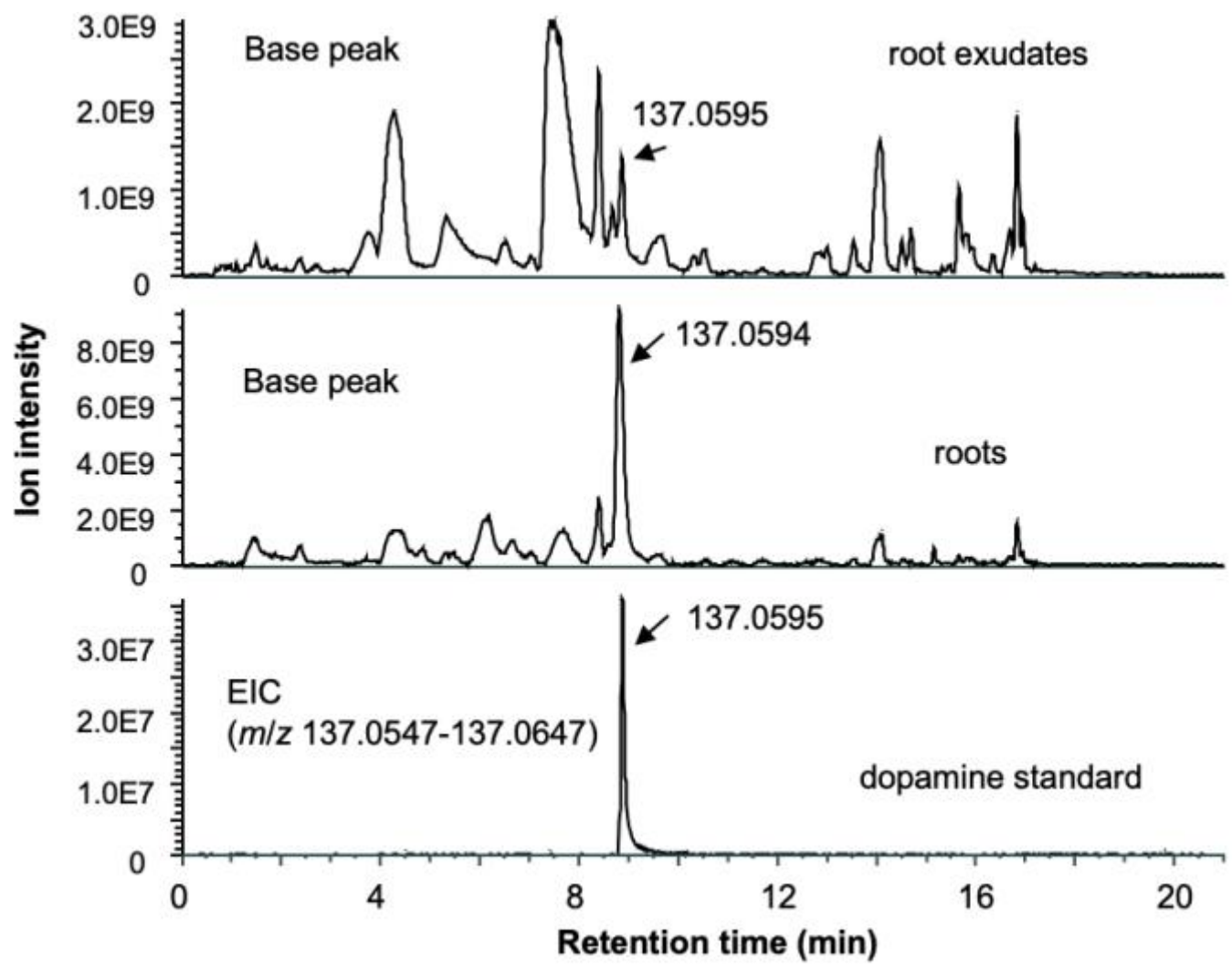


New Supplementary Fig. 9

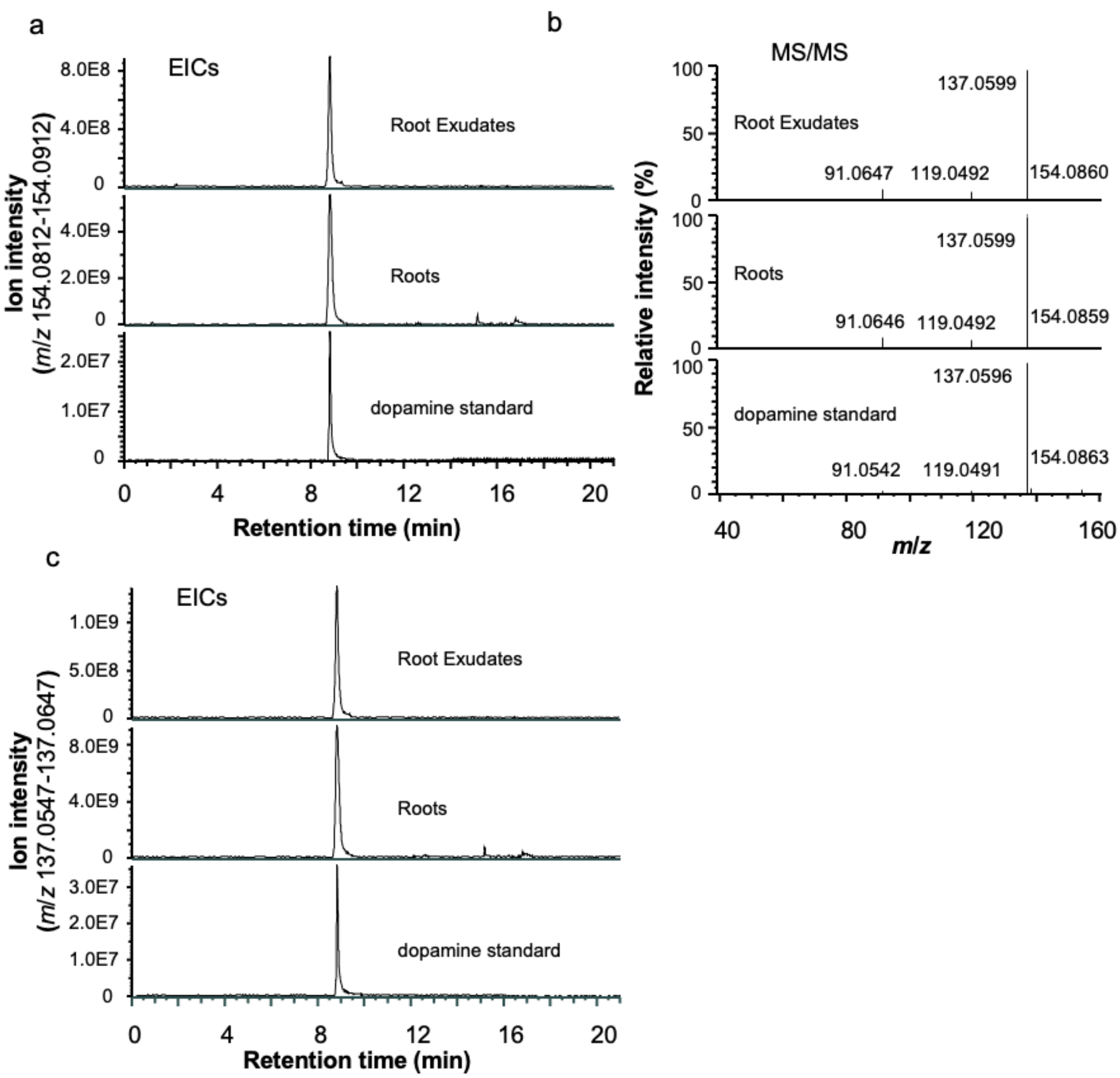


**Supplementary Fig. 9. Identification of dopamine in *B. distachyon* using LC-MS/MS. a** EICs of dopamine (precursor ion: *m/z* 154.086, $[M + H]^+$) in *B. distachyon* root exudates and

roots, aligned with a dopamine standard. **b** MS/MS spectra of dopamine detected in *B. distachyon* root exudates and roots, compared to the dopamine standard. **c** EICs of the dopamine in-source fragment ion (*m/z* 137.060, $[M + H - NH_3]^+$) in roots and root exudates, aligned with the dopamine standard. *B. distachyon* Bd21-3 plants were grown hydroponically, and roots along with the growth medium (containing root exudates) were harvested from 4-week-old plants for LC-MS/MS analysis.

2) In the data presented in Figure 2b, it is clear that dopamine is depleted in the presence of the SynCom versus Axenic (presumably because it is consumed as a growth substrate). But I am unconvinced that the Brachypodium lines show reproducible differences in their dopamine levels. When I inspect the variance in the measurements of the green data series in Figure 2b, it appears that the SEM is roughly 75% of the mean. To me, such variance is indicative of a highly variable measurement which I would interpret with caution.

We appreciate your point and have reanalyzed the data using ANOVA (see below). As you noted, we did not detect significant differences in dopamine abundance in root exudates among the ten *B. distachyon* lines under axenic conditions, likely reflecting high variability across lines. However, in the presence of the SynCom, dopamine levels differed significantly between certain lines, as shown in the revised Fig. 2b (see below).

Original Fig. 2b

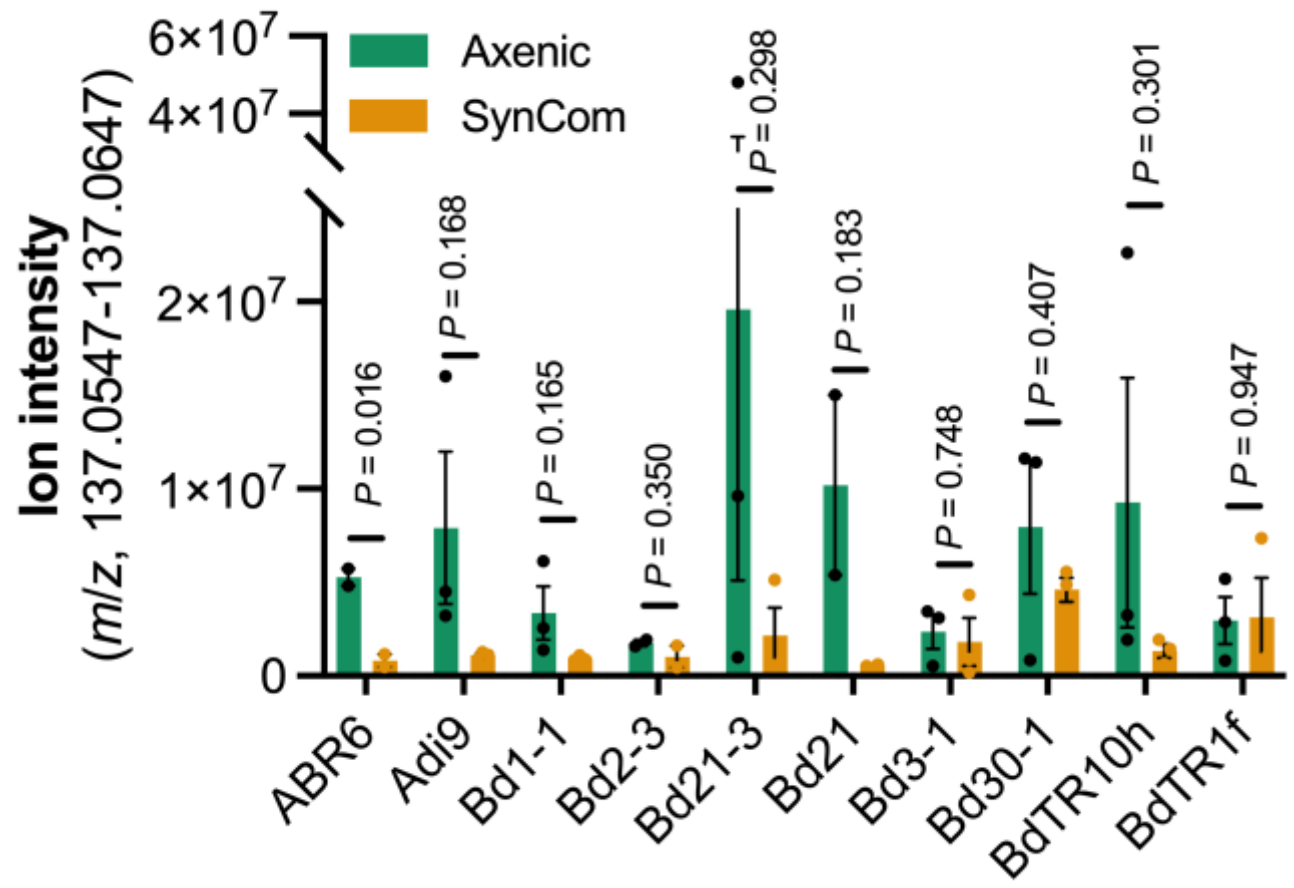


Revised Fig. 2b

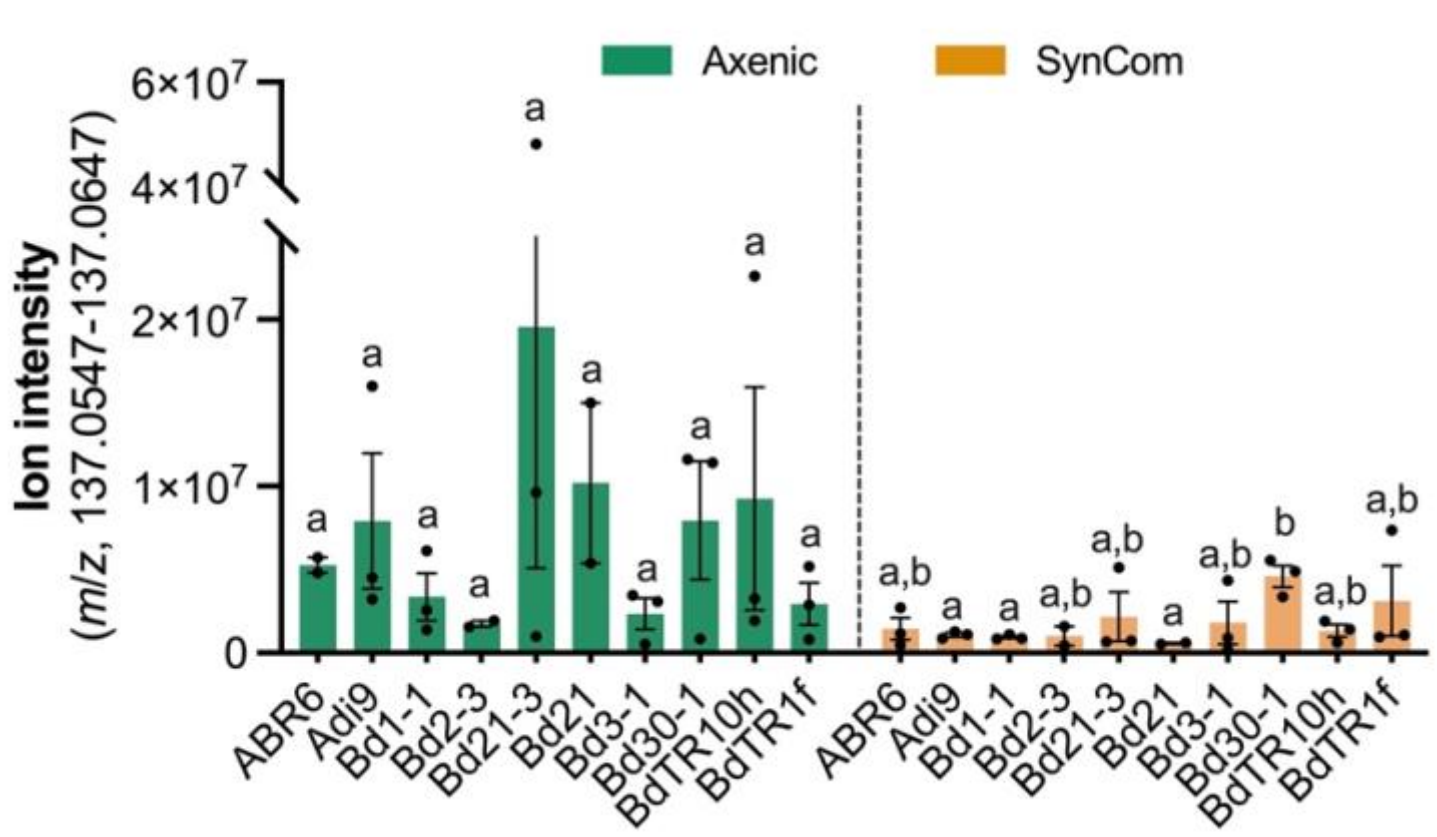

Fig2b legend: **b** shows that dopamine levels vary across ten *B. distachyon* lines in the absence (green) and presence (yellow) of the SynCom. Ten *B. distachyon* lines were grown in a hydroponic system in the absence and presence of a 16-member bacterial SynCom. Plant spent media were collected for LC-MS/MS analysis at week 4. Error bars represent the mean ± SEM of the peak height (*m*/*z* 137.0547-137.0647) ($n$ = 2 or 3). Statistical analyses were conducted separately for dopamine levels under axenic conditions and in the presence of the SynCom. Different letters (a-b) indicate significant differences as determined by Welch's ANOVA followed by Dunnett's T3 multiple comparison test ($P < 0.05$).

**We revised the manuscript accordingly and added the following text (Lines 183-186):**

"While dopamine levels in root exudates under axenic conditions showed relatively high variability, dopamine levels in the presence of the SynCom were generally consistent across replicates and exhibited significant differences between certain plant lines (Fig.2b)."

**We also added the following text in the discussion (Lines 318-320):**

"We acknowledge that dopamine levels measured under axenic conditions were associated with high variability. However, dopamine levels in root exudates in the presence of SynCom exhibited relative consistency across replicates and showed significant differences between certain lines (Fig. 2b)."

To summarise, the presented data have generally convinced me that Brachypodium plants exude dopamine, and also that dopamine can modulate microbiome composition. These are both novel and interesting findings. However, I am unconvinced whether Brachypodium lines show reproducible differences in their dopamine exudation, and therefore I do not see any evidence that dopamine explains any role in differential microbiome assembly between Brachypodium lines. As mentioned by the authors, there are no genetic knockouts of plant dopamine biosynthesis, which therefore makes it is difficult to design targeted experiments which dissect dopamine's role in microbiome assembly. But the correlative evidence present in this study is in my opinion insufficient to make a link between plant genetics, dopamine exudation, and microbiome composition.

We agree that our conclusion is based on multiple lines of evidence rather than direct evidence from plant mutants, and it is essential to state this explicitly in the manuscript. To address this, we have added the following text at the end of the discussion to clarify this limitation.

Lines 379-382: Although our findings suggest that dopamine plays a role in modulating rhizosphere microbial communities, further experimental validation is needed to confirm its functional effects on microbial physiology and community dynamics, particularly under natural field conditions.

Some minor points:
- In Supp Figure 9, I am a little bit confused by the differential units used to quantify the 13-C dopamine standard versus the endogenous dopamine in plant roots (mol/L versus g/gFW). This

prevents me from judging whether their measured plant dopamine levels fall within the linear range of their calibration curve.

We thank the reviewer for this thoughtful comment and acknowledge the potential confusion caused by the use of different units for the standard curve and endogenous dopamine measurements. In the revised manuscript, we have clarified this distinction in the Methods section. Specifically, the calibration curve was generated using [ring-$^{13}C_6$]-labeled dopamine dissolved in the extraction buffer, at concentrations of 0.5, 1.0, 2.0, 4.0, and 8.0 mM (expressed in mmol/L). In contrast, endogenous dopamine was extracted using 500 µL of the same buffer from approximately 100 mg of fresh root tissue and quantified in mg/g fresh weight. The use of molar concentrations for the standard curve was necessary due to the differing molecular weights between the isotope-labeled dopamine and endogenous dopamine. We have now explicitly stated in the Materials and Methods section that all measured endogenous dopamine concentrations fall within the linear dynamic range of the calibration curve (0.38 to 6.12 mg/g fresh tissue), thereby supporting the accuracy and reliability of our quantification.

We made the following changes to the Materials and Methods section.

**We added the following text in Lines 541-542:**

"This approach enables accurate quantification of endogenous dopamine within a dynamic range of 0.38 to 6.13 mg/g fresh tissues."

**Also, we added the following text in Lines 558-559:**

"The plant endogenous dopamine concentrations were converted to milligrams of $^{12}C$-dopamine per gram fresh weight."

- In Supp Figure 10 Panel A, I suspect that the reported calibrations are being disproportionately driven by the single data point in the bottom-left corner of the scatter plots. Have the authors considered removing that single data point and then recomputing whether the correlation is still significant?

We appreciate the reviewer's careful evaluation of Supplementary Fig. 12a (in the revised manuscript). We agree that the data point in the lower-left corner of the scatter plots in original Supplementary Fig. 12a may have a strong influence on the overall correlation. Following the reviewer's suggestion, we reanalyzed the data after removing this data point and found that the positive correlations between dopamine levels and plant growth traits remained statistically significant (Please see the following figures).

Therefore, we have retained the original figures without modifications in the revised manuscript.

Fig. 12a.

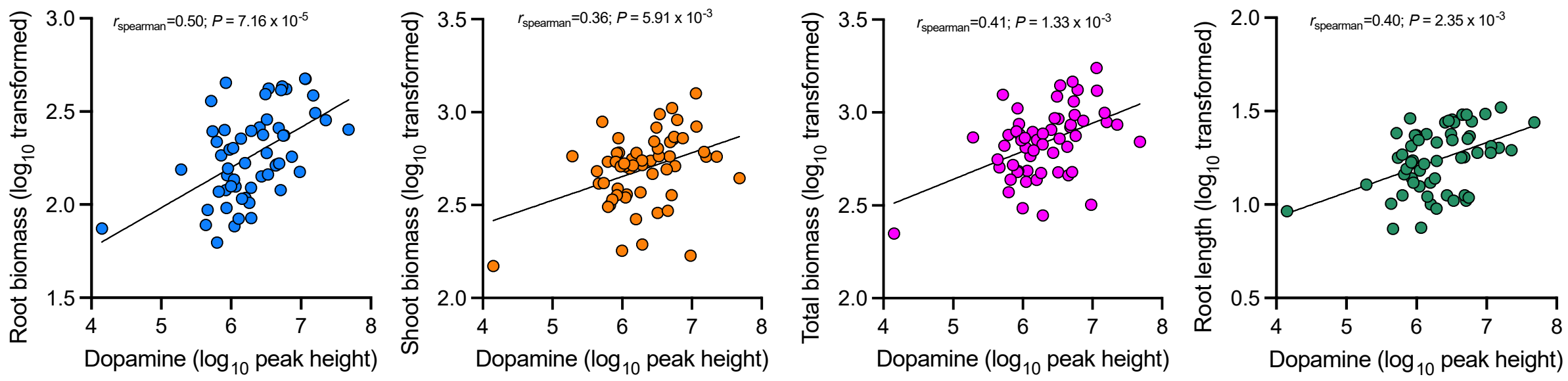


Removing the outlier:

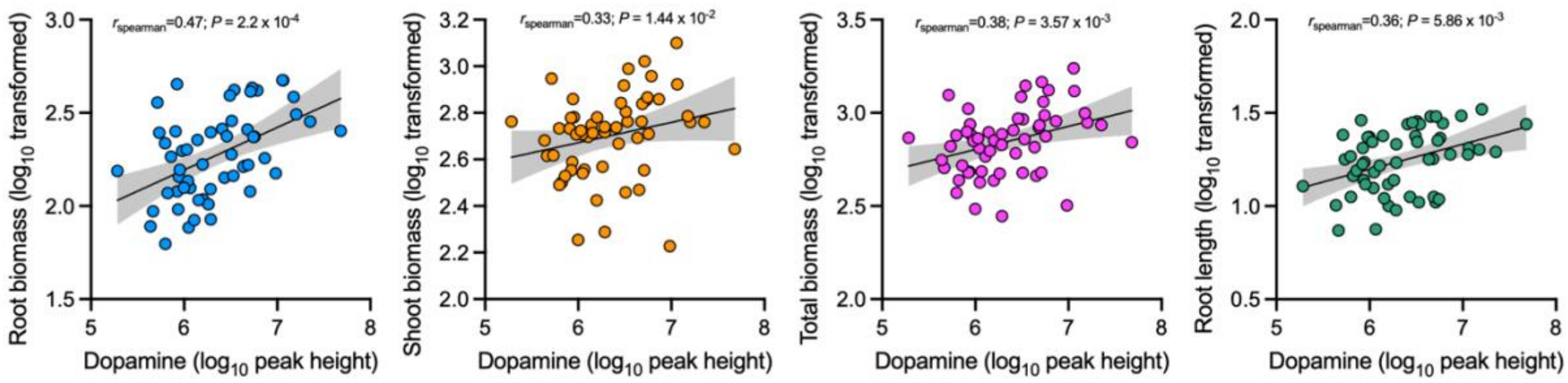


- In Supp Figure 10 Panel B, it seems that the growth response to exogenous dopamine follows an 'Inverse U-shape', whereby lower dopamine concentrations stimulate root growth, before higher concentrations seem to tend towards being toxic. Is this reflected in the figure caption 'Dopamine has a positive effect on plant growth'?

Good point, we have revised the figure caption of Supplementary Fig. 12 to "**Dopamine impacts plant growth in a dose-dependent manner**".

- Again in Supp Figure 10 Panel B, I cannot read the units of dopamine that were applied. (αM)?

We appreciate the reviewer's attention to detail. We have corrected this as shown below (Supplementary Figure 12b).

The revised Supplementary Fig. 12b.

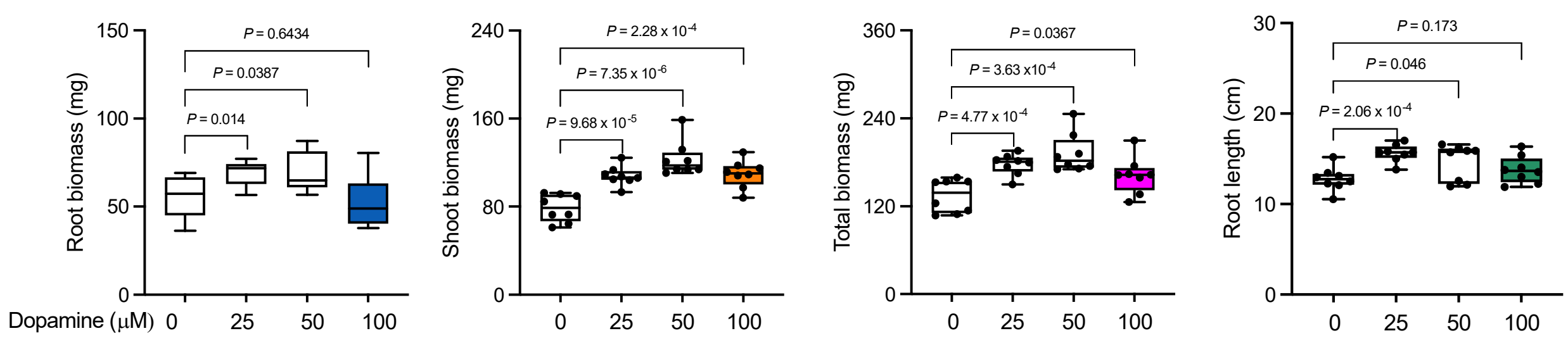

Responses to Reviewers' Comments

We thank both reviewers for their thoughtful evaluation of our manuscript entitled "Insights into the role of dopamine in rhizosphere microbiome assembly" (NCOMMS-24-49252A). We are grateful for the positive feedback on our manuscript as well as the additional constructive comments. In response, we have further revised the manuscript as detailed in the point-by-point replies below. Please note that the line numbers cited correspond to the revised version, and all edits in the manuscript are highlighted in green.

REVIEWER COMMENTS

Reviewer #1 (Remarks to the Author):

Thanks for the efforts, the manuscript is better now, while I still have some comments for its improvement. In the Results section (lines 219-222), without additional data, this section overstates the mechanistic evidence for dopamine's growth-promoting effect by citing previous literature. However, no experimental evidence is provided in the current study to support these mechanistic explanations. This weakens the Results section, as it blends speculation with data presentation. The authors should: provide direct experimental data (e.g., hormone quantification, ROS assays, nitrogen assimilation measurements) to substantiate these proposed mechanisms.

Response: We thank the reviewer for this important and constructive comment. We have removed this sentence (lines 230-233) from the Results section. We agree with the reviewer that direct experimental validation of these proposed mechanisms, such as hormone quantification, ROS measurements, and nitrogen assimilation assays, would substantially strengthen mechanistic understanding of dopamine's biological effects. However, these analyses are beyond the scope of the current study and will be important directions for future investigation.

While Actinobacteria are enriched upon dopamine treatment, the study does not provide any data to confirm that this enrichment translates to a beneficial functional outcome, such as enhanced plant growth promotion, pathogen suppression, or nutrient cycling.

Response: We thank the reviewer for this insightful comment and have revised the text as shown below. We agree that while our soil treatment experiments demonstrate a reproducible enrichment of Actinobacteria upon dopamine application, the current study does not directly test downstream ecosystem-level functions such as plant growth promotion, pathogen suppression, or nutrient cycling.

Our intention was not to claim direct functional outcomes from the observed taxonomic shifts, but rather to report a consistent and selective microbial response, particularly the enrichment of Actinobacteria.

In the revised manuscript, we added the following sentence to the Discussion (Lines 357-360): "Although Actinobacteria include many taxa with well-documented beneficial traits[42], functional

validation of dopamine-driven enrichment remains to be established and will be an important direction for future investigation."

In Fig 2b, under axenic conditions (without SynCom inoculation), dopamine levels vary widely across the ten B. distachyon lines. This large variability raises doubts about whether dopamine can be considered a key driver of the microbial shifts reported in Result 1. To strengthen the causal link, I strongly recommend the use of plant mutants impaired in dopamine biosynthesis or signaling, which would provide direct evidence of dopamine's role in shaping microbial community assembly. In addition, the number of biological replicates per line is only 2-3, and replicate variability is high. This substantially reduces the reliability of the conclusions. The authors should increase the number of replicates to improve statistical power and robustness of the findings.

Response: We thank the reviewer for this careful and constructive assessment and have revised the text and provided a new figure. We agree that the axenic-condition dataset in Fig. 2b shows substantial variability in dopamine levels across the ten *B. distachyon* lines. Regarding the suggestion of genetic validation using mutants impaired in dopamine biosynthesis or signaling, we fully agree that such approaches would provide the most direct evidence for dopamine's role in shaping microbiome assembly. However, this is beyond the scope of this project and not feasible at this time. To address the reviewer's concern regarding variability, we conducted an additional axenic experiment with increased biological replication ($n$ = 6 per line). This additional experiment provided improved statistical resolution, demonstrating statistically significant dopamine variation across lines under axenic conditions (Supplementary Fig. 11).

We deleted the following sentences (Lines 187-191):

"Additionally, we observed natural variation in the dopamine levels in root exudates of the ten *B. distachyon* lines (Fig. 2b, 2c, Supplementary Table 4). While dopamine levels in root exudates under axenic conditions showed relatively high variability, dopamine levels in the presence of the SynCom were generally consistent across replicates and exhibited significant differences between certain plant lines (Fig. 2b)."

We added the following sentences (Lines 181-194):

"In the initial experiment, dopamine levels in root exudates under axenic conditions showed substantial variability across the ten *B. distachyon* lines, and statistically significant differences between lines were not consistently observed (Fig. 2b; Supplementary Table 4). To further examine potential line-to-line variation, we performed an additional axenic experiment with increased biological replication ($n$ = 6 per line), which supported reproducible differences in dopamine abundance among lines (Supplementary Fig. 11). In contrast, SynCom inoculation was consistently associated with reduced dopamine abundance across lines (Fig. 2b, 2c), suggesting microbial utilization of dopamine and/or altered dopamine production or exudation in response to microbial colonization."

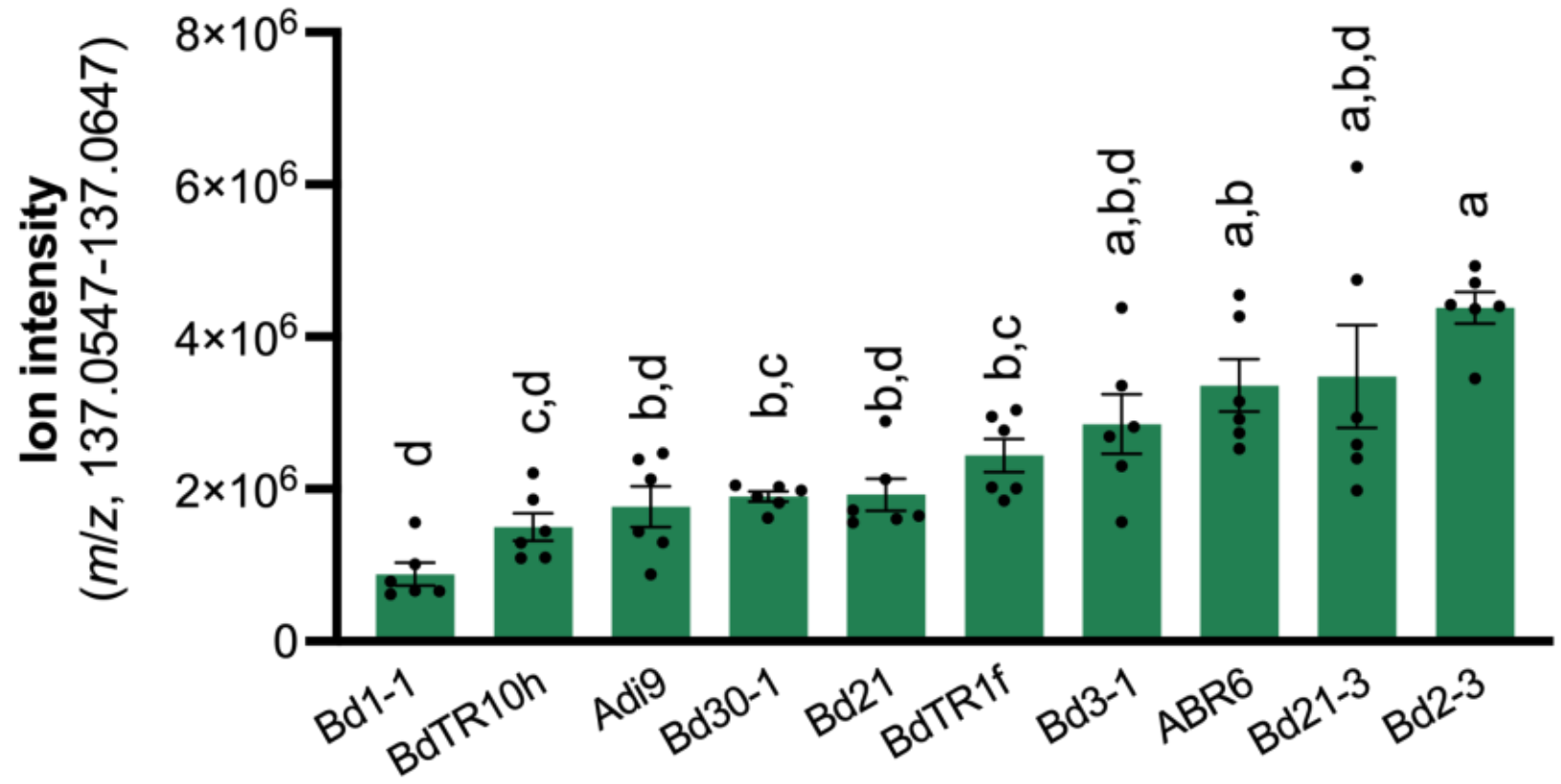


Supplementary Fig. 11. Dopamine levels in root exudates vary across ten *B. distachyon* lines under axenic conditions. Ten *B. distachyon* lines were grown in a hydroponic system in the absence of the 16-member bacterial SynCom. Plant spent media were collected at week 4 for LC-MS/MS analysis. Error bars represent the mean ± SEM of the peak height (*m/z* 137.0547-137.0647) ($n = 6$). Different letters (a-d) indicate significant differences as determined by Welch's ANOVA followed by Dunnett's T3 multiple comparison test ($P < 0.05$).

In Fig 2c, the consistent and significant depletion of dopamine in the presence of the SynCom strongly suggests that dopamine is primarily utilized as a nutrient source by the microbial community. Furthermore, the marked reduction in variation of dopamine levels across the ten B. distachyon lines upon inoculation indicates that microbial metabolism is the dominant factor controlling dopamine abundance in the rhizosphere, overwhelming any underlying plant genotypic variation. It points to a model where dopamine is a common substrate that is consumed, and its level is a consequence of microbial activity rather than a cause of specific microbial enrichment.

Response: We thank the reviewer for this comment which we have addressed through changes to the text as shown below. We agree that the consistent decrease in dopamine levels in the presence of the SynCom (Fig. 2c) suggests microbial utilization of dopamine within the community context. However, we cannot rule out the possibility that the presence of the microbial community also influences dopamine biosynthesis and/or exudation by roots. In addition, our *in vitro* bacterial growth assays showed that dopamine could either promote or suppress bacterial growth, suggesting that difference in dopamine could affect rhizosphere community structure.

We added the following sentence at Lines 221-224:
"Together with our observation that dopamine may be utilized by members of the microbial community (Fig. 2c), our findings suggest that dopamine may function both as a microbial growth substrate and as a potential modulator of microbial communities through other mechanisms."

We also added the following sentence at Lines 402-403:

"Our results further suggest a complex and dynamic interaction in which dopamine both influences and is influenced by rhizosphere microbial communities."

The data presented in Fig. 4b suggest that the effects of dopamine and norepinephrine on the soil microbial community at the phylum level are strikingly similar. This observation challenges the manuscript's overarching narrative that specifically highlights dopamine. The authors dismiss the role of norepinephrine based on its trace level in B. distachyon roots compared to dopamine. However, this soil treatment experiment directly demonstrates that the soil microbiome is highly responsive to exogenous norepinephrine. This raises a critical question: could norepinephrine play a role just as important as dopamine? The current data cannot rule out this possibility. To substantiate the claim of dopamine's distinct importance, a parallel and comparative analysis is essential. Specifically, the authors should perform the same correlation analysis between norepinephrine (and other relevant exometabolites) and the SynCom member abundances that was conducted for dopamine. If dopamine shows uniquely strong and specific correlations with the bacterial, it would strengthen their case. Conversely, if norepinephrine shows similar correlative patterns, it would necessitate a significant revision of the discussion, framing the effect as a more general catecholamine phenomenon rather than a dopamine-specific story.

Response: We thank the reviewer for this thoughtful comment and have made specific changes to address this point as described below by focusing the discussion more on catecholamines versus just dopamine. We agree that the soil treatment experiments demonstrate that norepinephrine is biologically active and capable of influencing soil microbial community structure. We do expect that abundance is an important factor for compounds that are serving as growth substrates, and dopamine was consistently detected at high abundance in both roots and root exudates, whereas norepinephrine was detected only at trace levels in roots and was not detected in root exudates using our LC–MS/MS workflow. Consequently, norepinephrine abundance could not be quantified in root exudates across samples, preventing correlation analysis between norepinephrine levels and SynCom member abundances, which is one reason why we don't feature more prominently in the paper.

You are also correct that Fig. 4b shows partially overlapping phylum-level responses. However, principal coordinate analysis (Fig. 4a) showed that dopamine- and norepinephrine-treated soils formed distinct community clusters, particularly under high-dose treatments. In addition, zOTU-level analyses (Fig.4c and Supplementary Fig.16 & 17) demonstrated that the specific microbial taxa responding to dopamine and norepinephrine differed substantially, with only limited overlap among them. These findings suggest that dopamine and norepinephrine exert overlapping but distinct roles in shaping rhizosphere microbial communities.

We also agree that our findings do not exclude the possibility that norepinephrine and other catecholamine-related metabolites may contribute to shaping rhizosphere microbial communities. Accordingly, we revised the Discussion as follows.

We replaced the following sentence at Lines 337-339:

"Functional tests in soil revealed that catecholamines, particularly dopamine, could restructure the soil microbiota by specifically enriching Actinobacteria populations, underscoring the selective influence of dopamine on rhizosphere microbial communities."

with the following sentences (Lines 339-350):

"Functional tests in soil revealed that catecholamine-related metabolites, including dopamine and norepinephrine, can restructure soil microbial communities, particularly by enriching Actinobacteria populations. Although both compounds induced partially overlapping responses at the phylum level, deeper community analyses revealed distinct treatment-specific effects at the zOTU and β-diversity levels, suggesting that structurally related catecholamines may exert both shared and compound-specific influences on rhizosphere microbial communities. Importantly, dopamine was consistently detected at high abundance in both roots and root exudates, whereas norepinephrine was detected only at trace levels in roots and was not detected in root exudates under our experimental conditions. This difference in endogenous abundance limited our ability to perform comparable correlation analyses for norepinephrine and supports the emphasis on dopamine as the dominant detectable catecholamine associated with microbial community variation in *B. distachyon*."

Specifically, the text states: 1) "Features unique to an individual line accounted for 18% of all features; for each individual line, the number varied between 6% to 12% of all features" (related to Supplementary Fig. 6b); and 2) "The proportion of increased features varied from 6% to 27%... while decreased features ranged from 2% to 18%" (related to Supplementary Fig. 7). However, Supplementary Fig. 6b is a simple pie chart without any numerical labels or a data table, making it impossible for a reader to confirm the stated 18% value or the per-line percentages. Similarly, Supplementary Fig. 7 consists of ten volcano plots, a format designed to show significance versus magnitude of change for individual features, but not to convey the overall proportion of features that were increased or decreased in a given line. The authors can modify these figures to directly visualize the quantitative summaries they describe in the text.

Response: We thank the reviewer for this helpful comment and agree that the original figures did not directly visualize the quantitative summaries described in the text. To address this concern, we have updated the figures to more clearly present the corresponding numerical information. Specifically, we added a figure, Supplementary Fig. 6c, showing the percentages of unique metabolic features detected in the root exudates of each of the ten lines under axenic conditions. In addition, we revised Supplementary Fig. 7 by adding the percentages of increased, decreased, and unchanged features for each line directly onto the figure panels. These additions allow readers to readily interpret the proportions of differential features while retaining the volcano plots for visualization of feature significance and magnitude of change.

We believe these revisions substantially improve the clarity and interpretability of the figures.

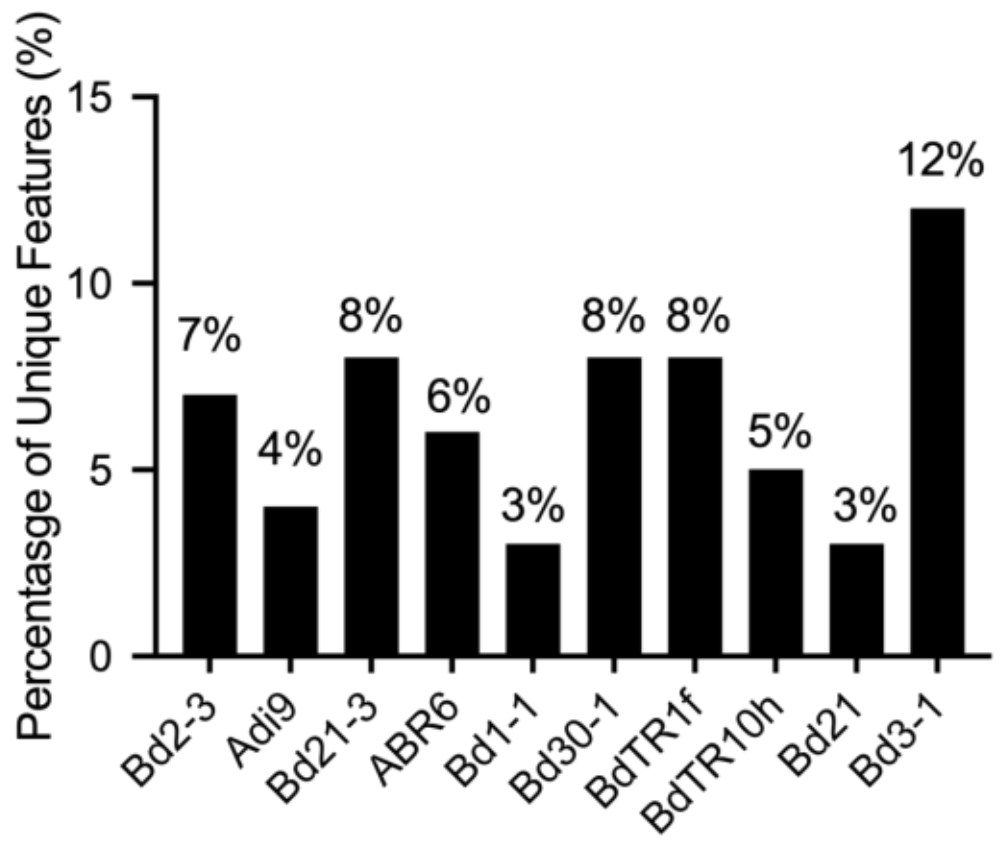


Supplementary Fig. 6c shows the percentages of unique features detected in the root exudates of the ten lines in the absence of the SynCom. Overall, features unique to an individual line accounted for 18% of all features.

Revised Supplementary Fig. 7.

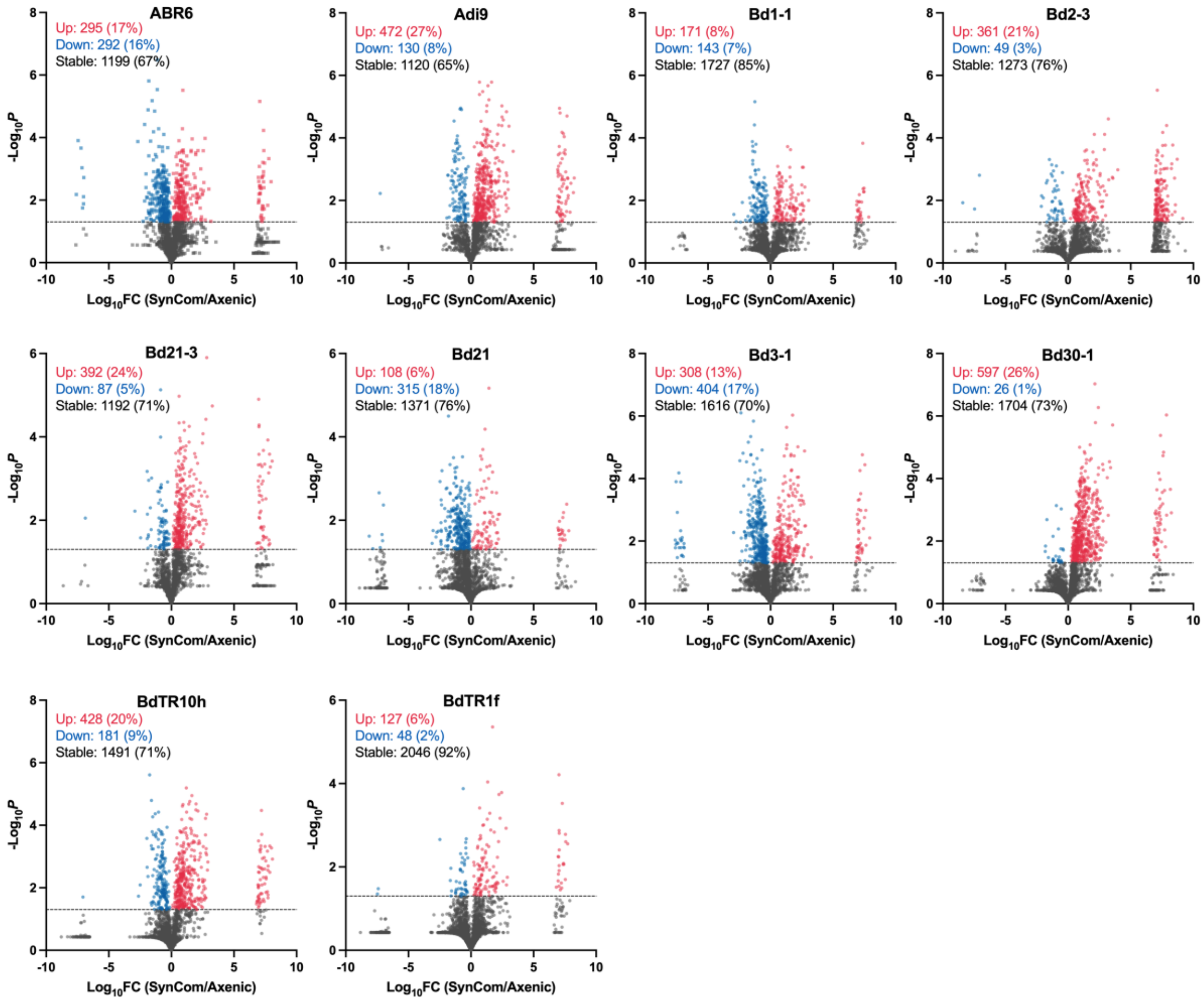


Supplementary figures use a mix of mol/L and g/gFW for dopamine quantification, which can be confusing. A consistent unit or a clear explanation for the difference should be provided in the figure legends.

Response: We thank the reviewer for this helpful comment. To clarify the use of different units, we added the following text to the legend of Supplementary Fig. 12:

"[Ring-$^{13}C_6$]-labeled dopamine was spiked into the liquid tissue extract as an internal standard. The calibration curve was used to calculate the endogenous $^{12}$C-dopamine concentration within the extract and is presented in µM units to allow direct comparison between $^{12}$C- and $^{13}$C-dopamine values."

The stress value for the NMDS plot (Supplementary Fig. 5) is not provided, which is necessary to interpret the reliability of the ordination.

Response: We thank the reviewer for this helpful comment. We have now added the stress value to the revised Supplementary Fig. 5 legend.

The choice of Bd21-3 for detailed follow-up experiments (dopamine quantification in roots, plant growth assays) is not justified regarding its representativeness among the ten lines.

Response: We thank the reviewer for this comment. We've added this information as shown below. Bd21-3 was selected for the detailed follow-up experiments primarily because it is one of the most widely used reference lines in the *B. distachyon* research community and is well-suited for controlled hydroponic growth, physiological characterization, and downstream experimental manipulation. We agree that explicitly justifying the choice of Bd21-3 would improve the manuscript. Therefore, we have revised the Materials and Methods section to clarify the rationale for selecting this line

We added the following sentence at Lines 432-434:
"Bd21-3 was selected for the follow-up experiments primarily because it is one of the most widely used reference lines and is well-suited for hydroponic growth and physiological characterization."

Dopamine was likely dissolved in a solvent (e.g., methanol, water) for soil application. A control with the solvent alone should have been included to rule out non-specific effects.

Response: We thank the reviewer for this comment. We would like to clarify that dopamine and norepinephrine were directly mixed into the soil as powders and were not dissolved in methanol or any other solvent for the soil treatment experiments. After metabolite addition, water was added across all treatments to maintain a soil moisture content of approximately 24.5%. The control treatment underwent the same experimental procedure and received the same amount of water, but without the addition of dopamine or norepinephrine.

To improve clarity, we have revised the Materials and Methods section to explicitly state that the metabolites were directly added to soil without solvent dissolution and that the control treatment received identical handling and watering addition.

We changed this sentence (Lines 536-537):

"Control soil was watered without the addition of any metabolites."

to the following sentence (Lines: 537-538):

"Control soil received the same handling and water addition but without the addition of dopamine or norepinephrine.".

Reviewer #2 (Remarks to the Author):

I thank the authors for their thoughtful, respectful and comprehensive response to both reviewers. Having carefully read the authors' response to reviewers document, I now feel much more comfortable that the conclusions written in the manuscript text are supported by the measurements conducted.

Response: We sincerely thank the reviewer for their careful re-evaluation of our manuscript and for the positive assessment.

I have only one small point of dissatisfaction: It involves Figure Panel 2b. For these data, I do not feel that the conclusions reported in the text (including the caption text) are representative of the measurements. I have various points of critique:

1) In the Caption Text of Figure 2, it states: "b shows that dopamine levels vary across ten B. distachyon lines" . In my interpretation, this is factually incorrect - according to the ANOVA-Post-Hoc-Test, the dopamine levels in all of the axenic Brachypodium lines are encompassed by the letter "a", indicating that dopamine levels do NOT reproducibly vary from line to line.

Response: Thank you for your comment. As noted above, we repeated the experiment under axenic conditions with increased biological replication ($n$ = 6 per line), which provided improved statistical resolution and supported reproducible line-to-line variation in dopamine abundance under axenic conditions (Supplementary Fig. 11).

We revised the Fig. 2b legend as follows: "b shows dopamine levels across ten *B. distachyon* lines in the absence and presence of a 16-member SynCom."

2) In the body text line 181, it states: "we observed natural variation in the dopamine levels in root exudates of the ten B. distachyon lines". Again, I don't feel that this conclusion is supported by the data, which is indicative of technical variation rather than biological.

Response: Thank you for this important point. As noted above, we repeated the experiment under axenic conditions with increased biological replication ($n$ = 6 per line), which provided improved statistical resolution and supported reproducible variation in dopamine abundance among lines under axenic conditions.

We replaced the following sentence (Lines 187-188):

"Additionally, we observed natural variation in the dopamine levels in root exudates of the ten *B. distachyon lines* (Fig. 2b, 2c, Supplementary Table 4)."

with the following sentences:

"In the initial experiment, dopamine levels in root exudates under axenic conditions showed substantial variability across the ten *B. distachyon* lines, and statistically significant differences between lines were not consistently observed (Fig. 2b; Supplementary Table 4). To further examine potential line-to-line variation, we performed an additional axenic experiment with

increased biological replication (*n* = 6 per line), which supported reproducible differences in dopamine abundance among lines (Supplementary Fig. 11)."

3) In the body text lines 185-186, it states that: "dopamine levels in the presence of the SynCom were generally consistent across replicates and exhibited significant differences between certain plant lines (Fig. 2b) . In my interpretation, this statement is in a 'grey zone', whereby the statement is factually true, but doesn't represent the major findings of the experiment. To clarify, root exudates are a notoriously variable sample type. It is to be expected that these measurements of dopamine levels in root exudates in an n=3 experiment will be highly variable. But in my interpretation, the primary finding is of this experiment is actually presented in Figure 2c, where the authors convincingly show that SynCom presence results in lower dopamine levels versus axenic, presumably because some of the strains have consumed the plant-exuded dopamine as a metabolic substrate. In contrast, I am not convinced that the authors have reproducibly measured biologically meaningful line-to-line dopamine variation. I accept that the chosen test has resulted in certain SynCom-treated lines being annotated with either 'a' or 'b', but upon closer inspection, these effect sizes are extremely small and probably biologically insignificant.

Response: We thank the reviewer for this thoughtful assessment. We agree that exudate dopamine measurements were variable, particularly with lower biological replication. We also agree that dopamine levels decreased significantly in the presence of the SynCom (Fig. 2c), suggesting potential microbial utilization and/or altered plant dopamine production in response to microbial colonization.

Accordingly, we revised the manuscript by softening the interpretation of line-to-line differences under SynCom conditions.

We deleted the sentence (Lines 188-191):

"While dopamine levels in root exudates under axenic conditions showed relatively high variability, dopamine levels in the presence of the SynCom were generally consistent across replicates and exhibited significant differences between certain plant lines (Fig. 2b)."

We added the following sentence (Lines 191-194):

"In contrast, SynCom inoculation was consistently associated with reduced dopamine abundance across lines (Fig. 2b, 2c), suggesting potential microbial utilization of dopamine and/or altered dopamine production or exudation in response to microbial colonization."

4) About the choice of statistics for Figure Panel 2B, I am not sure whether the choice of tests is appropriate. The caption text states: "statistical analyses were conducted separately for dopamine levels under axenic conditions and in the presence of the SynCom. Different letters (a-b) indicate significant differences as determined by Welch's ANOVA followed by Dunnett's T3 multiple comparison test ($P < 0.05$)." If the authors really want to present this conclusion in the published manuscript, can they please justify the following: (a) Why were axenic- and SynCom- treated

split and not bundled? (b) Why was Welch's ANOVA used instead of an alternative test? (c) Why was Dunnett's test used instead of an alternative test?

Response: We thank the reviewer for the comment. We have clarified our rationale as follows:

1) Why were axenic and SynCom-treated samples analyzed separately?
Our goal was to evaluate line-to-line variation within each biological context rather than to test an interaction between plant line and microbial condition. Because variance structure differed markedly between axenic and SynCom conditions, a pooled model would violate homoscedasticity assumptions even with Welch-type corrections; we therefore analyzed conditions separately to characterize line-level variation within each biological context.

2) Why was Welch's ANOVA used?
We used Welch's ANOVA because it does not assume equal variances across groups and is more robust for the initial exudate dataset under axenic conditions, which exhibited substantial variability across lines. This approach is widely recommended as a conservative alternative to standard ANOVA when variance equality cannot be confidently assumed.

3) Why was Dunnett's T3 used?
We used Dunnett's T3 test as the post hoc test because it is specifically designed for use following Welch's ANOVA and does not assume equal variances or equal sample sizes. This makes it appropriate and consistent with the choice of the overall test.

We have added the following sentences in the Methods (Lines 608-613):

"Welch's ANOVA and Dunnett's T3 tests were used for datasets exhibiting heteroscedasticity, including root exudate metabolite measurements, because these approaches do not assume equal variances among groups and are more robust for datasets with unequal variances and modest sample sizes. Statistical analyses for dopamine measurements under axenic and SynCom conditions were conducted separately to evaluate variation among plant lines within each biological condition independently."

Again, I thank the authors for their extensive modifications to the revised manuscript, which overall have reassured me that their conclusions are indeed supported by their measurements.

Response: We sincerely thank the reviewer for this encouraging assessment of our revised manuscript. We appreciate the careful evaluation and are pleased that the revisions clarified the analyses and strengthened support for our conclusions.
