# Supplementary material for: Insights into the role of dopamine in rhizosphere microbiome assembly
Source: Nat Commun. 2026 Jul 29;17:9212. doi: 10.1038/s41467-026-76211-1 (PMC13522444; doi:10.1038/s41467-026-76211-1)
Supplement: Supplementary file 3 [file 41467_2026_76211_MOESM1_ESM.pdf]

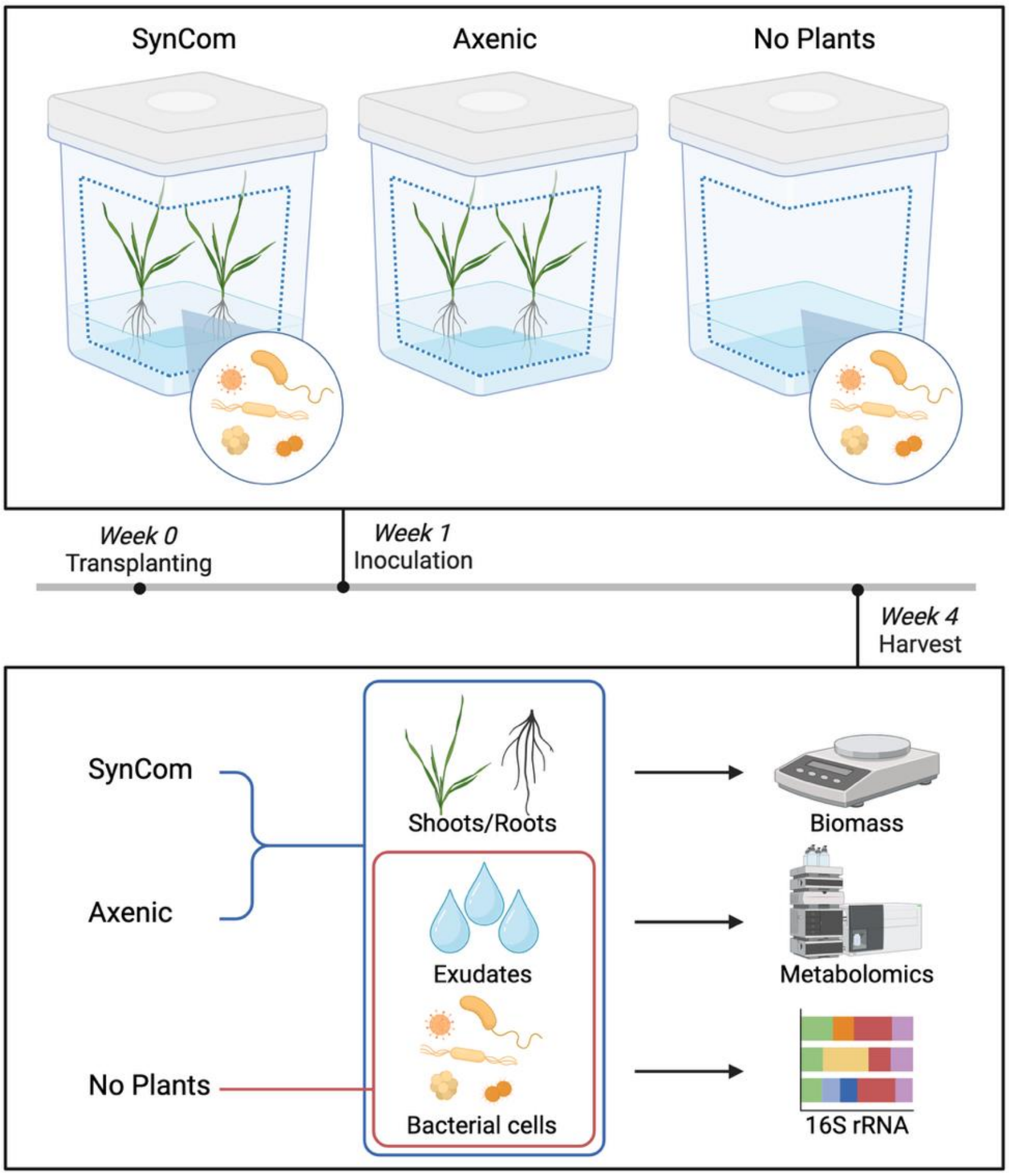

Supplementary Fig. 1. Schematic view of the experimental design for exploring the genetic variation of root exudates in plant-microbiome interactions. One-week-old hydroponically grown seedlings of ten *B. distachyon* lines were inoculated with a 16-member bacterial SynCom. Phenotypes were measured after an additional three-week incubation. Plants without the SynCom were used as the axenic controls. Exudates with the SynCom without plants were included (No Plants). We sampled the plant spent medium for exudate analysis by LC-MS/MS at week 4. The figure was created in BioRender. Ding, Y. (2026) https://BioRender.com/zo23maz.

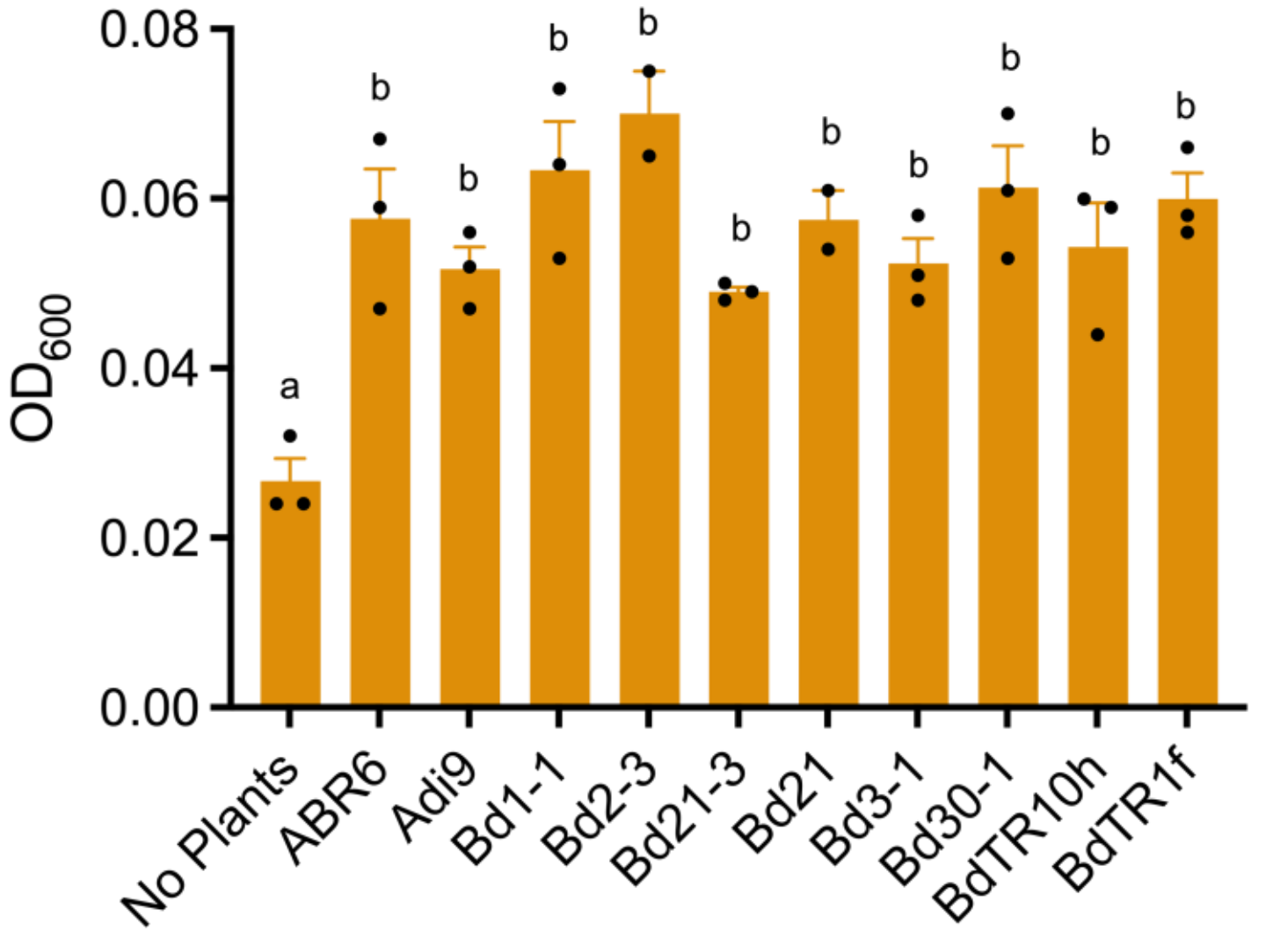

Supplementary Fig. 2. The growth of the SynCom bacteria in the plant spent medium as determined by the final OD_{600} measurement. Error bars represent mean ± SEM (*n* = 3 biologically independent samples except for Bd2-3 and Bd21 (*n* = 2)). Within the plot, different letters (a-b) represent significant differences (one-way ANOVA followed by Tukey's test corrections for multiple comparisons; $P < 0.05$). No Plants was used as the Control. *P* values were provided in the source data.

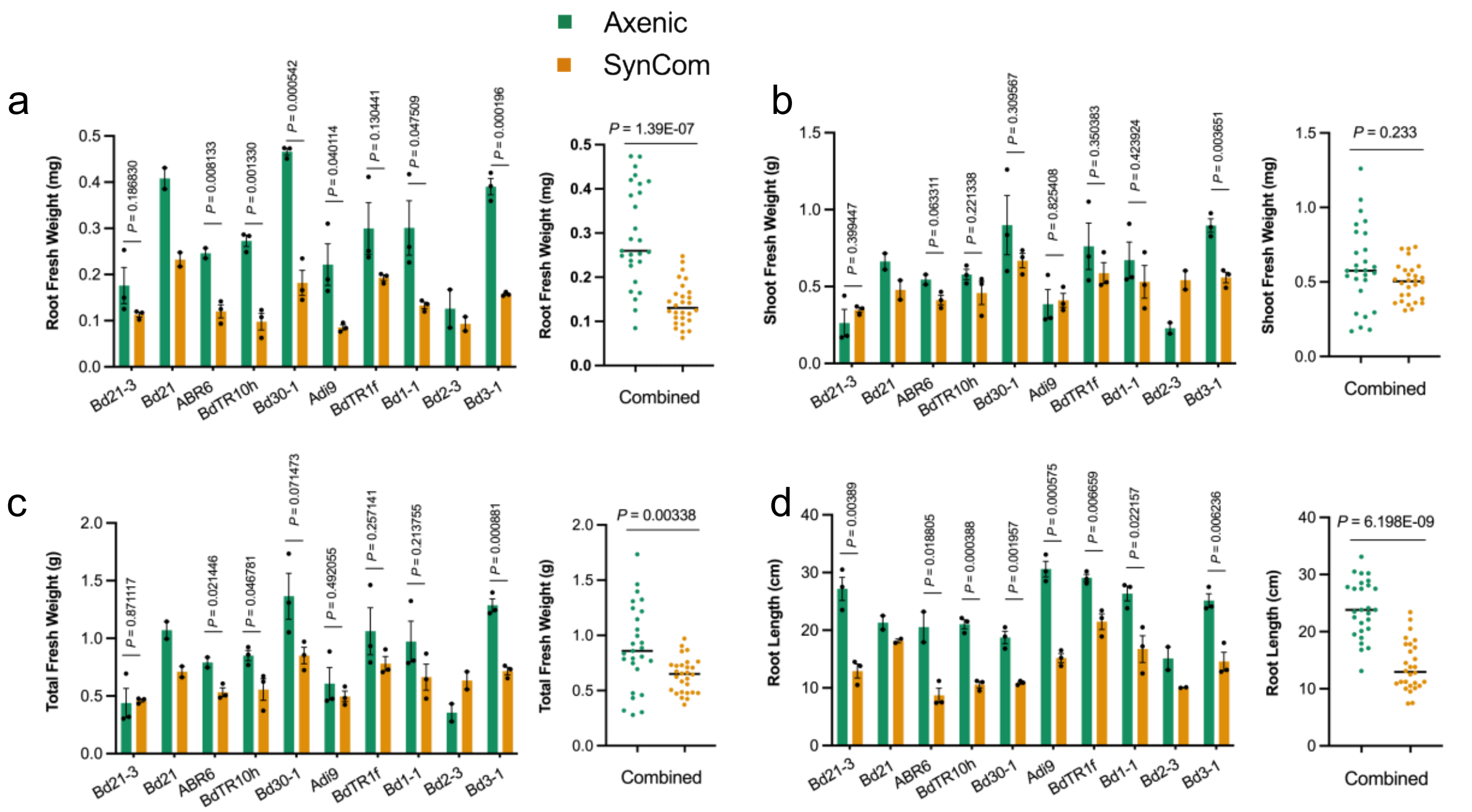

Supplementary Fig. 3. Phenotypic variation of ten *B. distachyon* lines in the absence and presence of a SynCom. Ten *B. distachyon* lines were grown in a hydroponic system for one week, and then half of them were inoculated with a 16-member bacterial SynCom. Phenotypes, including fresh root biomass (**a**), fresh shoot biomass (**b**), total fresh biomass (**c**), and root length (**d**), were measured after an additional three-week incubation. Error bars represent mean ± SEM (n = 3 biologically independent samples except for Bd2-3 and Bd21 (n = 2)). Combined analyses of each phenotype in the absence and presence of the SynCom were also performed. The *P*-value was calculated using a two-sided student's *t*-test.

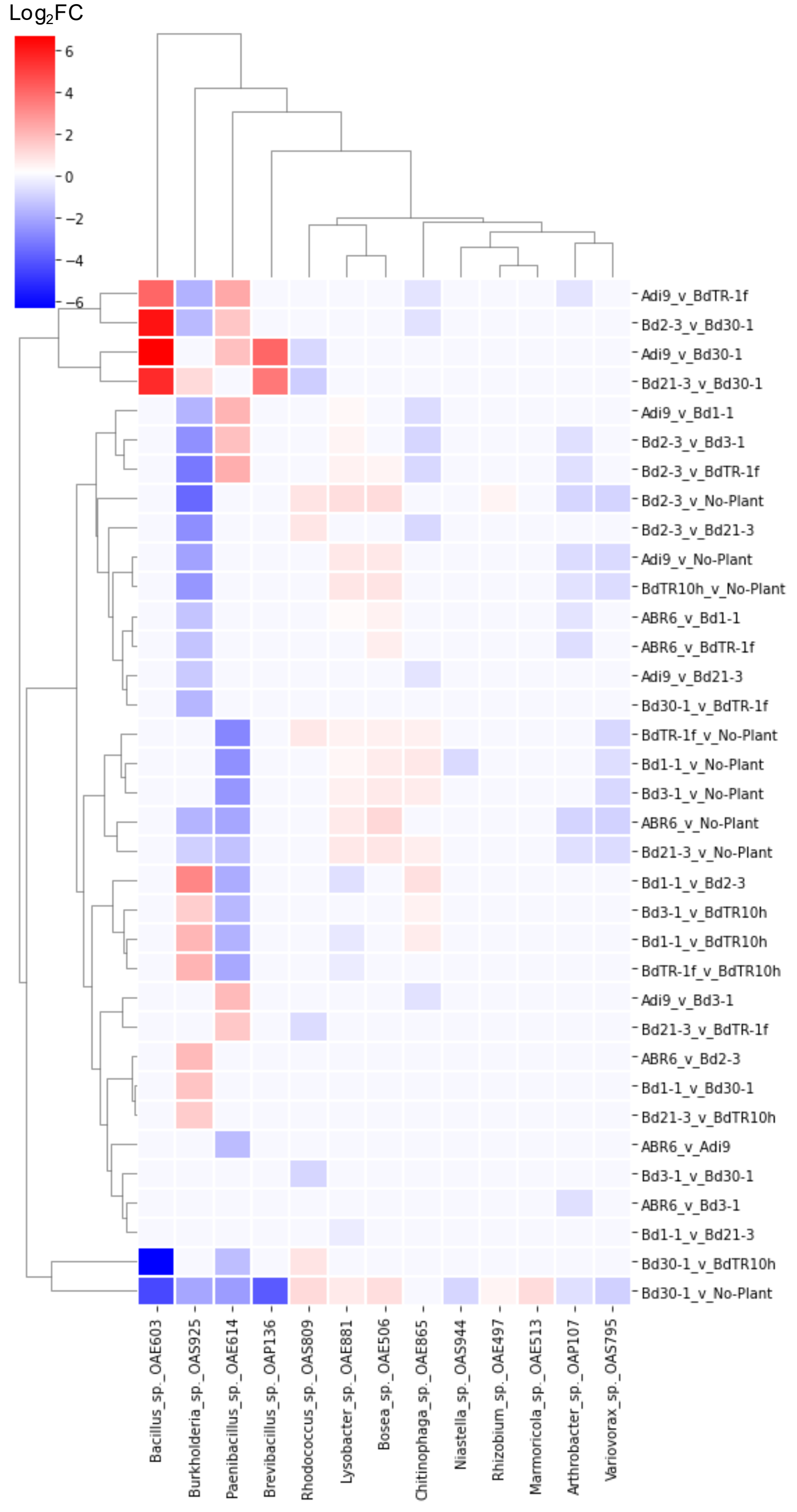

Supplementary Fig. 4. Pairwise comparisons of SynCom member abundances across *B. distachyon* lines. Deseq2 was used to compare SynCom member abundances in the spent media of *Brachypodium* lines and No Plant. Log_2 fold change (Log_2FC) values of SynCom members that were significantly different (adjusted *P*-value <0.05) were then clustered (non-significant Log_2 FCs were set to 0 for clarity). Red, >0, indicates that the isolate was significantly higher in the second condition listed whereas blue, <0, indicates that the isolate was significantly higher in the first condition listed.

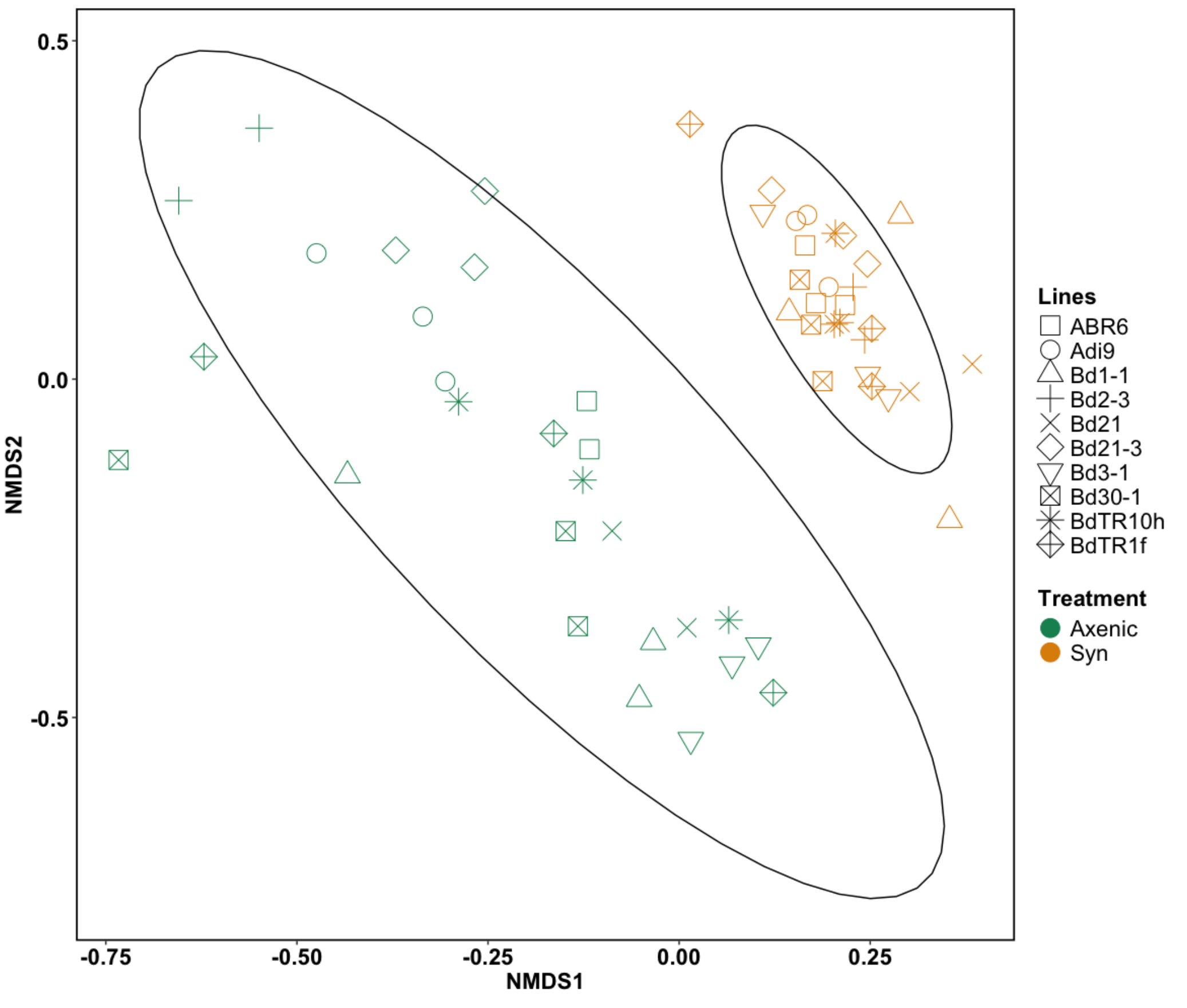

Supplementary Fig. 5. Non-metric multidimensional scaling (NMDS) plot for untargeted metabolomics analysis. The plot was constructed based on peak heights of features detected in the plant spent medium of ten *B. distachyon* lines in the absence and presence of the SynCom (*n* = 3 biologically independent samples except for Bd2-3 and Bd21 (*n* = 2)) . Stress=0.11.

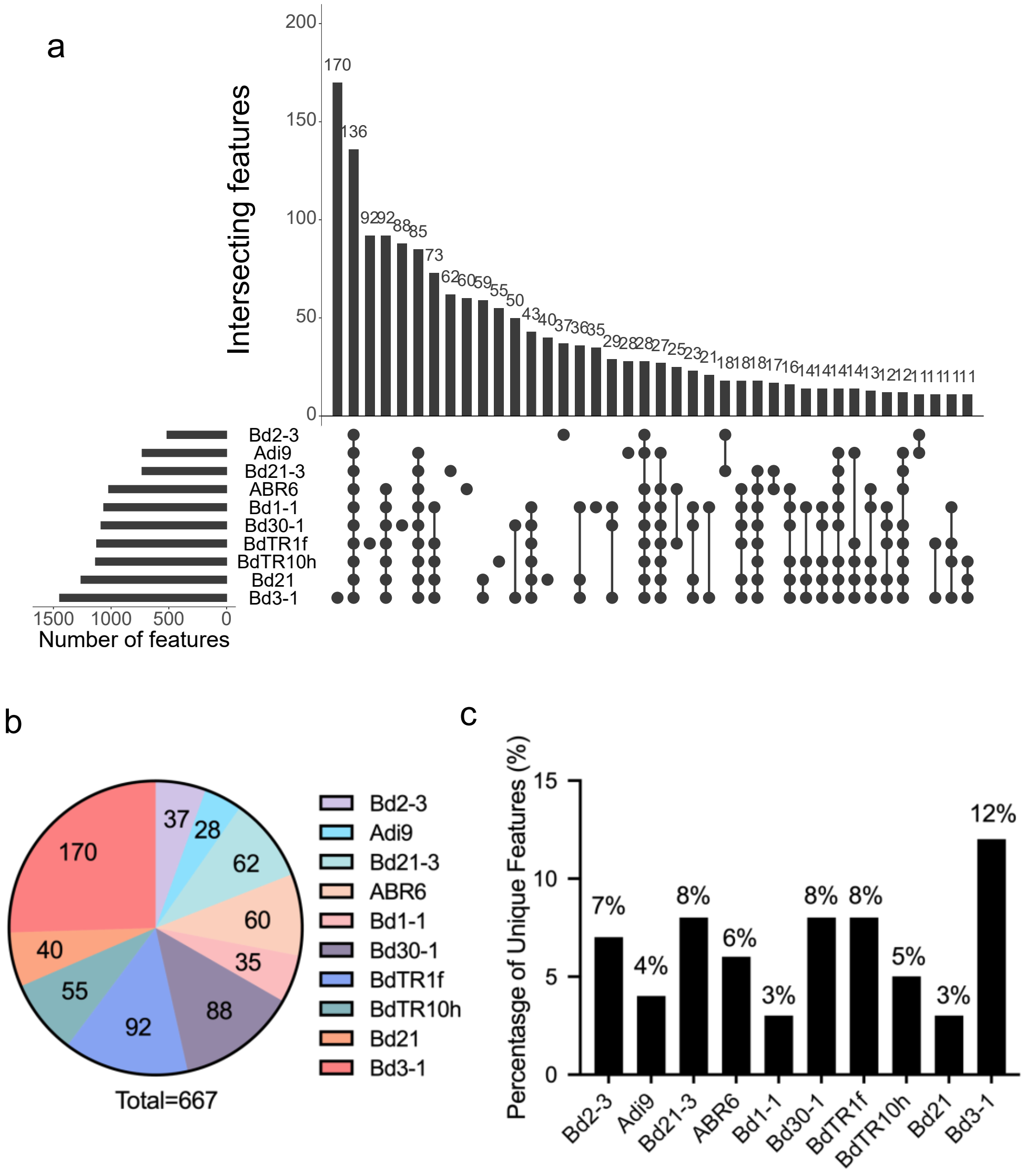

Supplementary Fig. 6. The number of features detected in root exudates. a The UpSet plot shows the number of shared filtered features detected in root exudates of ten *B. distachyon* lines in the absence of the SynCom. One hundred thirty six features are shared by the ten *Brachypodium* lines. **b** The pie chart shows the number of unique features detected in root exudates of individual *B. distachyon* lines in the absence of the SynCom. **c** shows the percentages of unique features detected in the root exudates of the ten lines in the absence of the SynCom. Overall, features unique to an individual line accounted for 18% of all features.

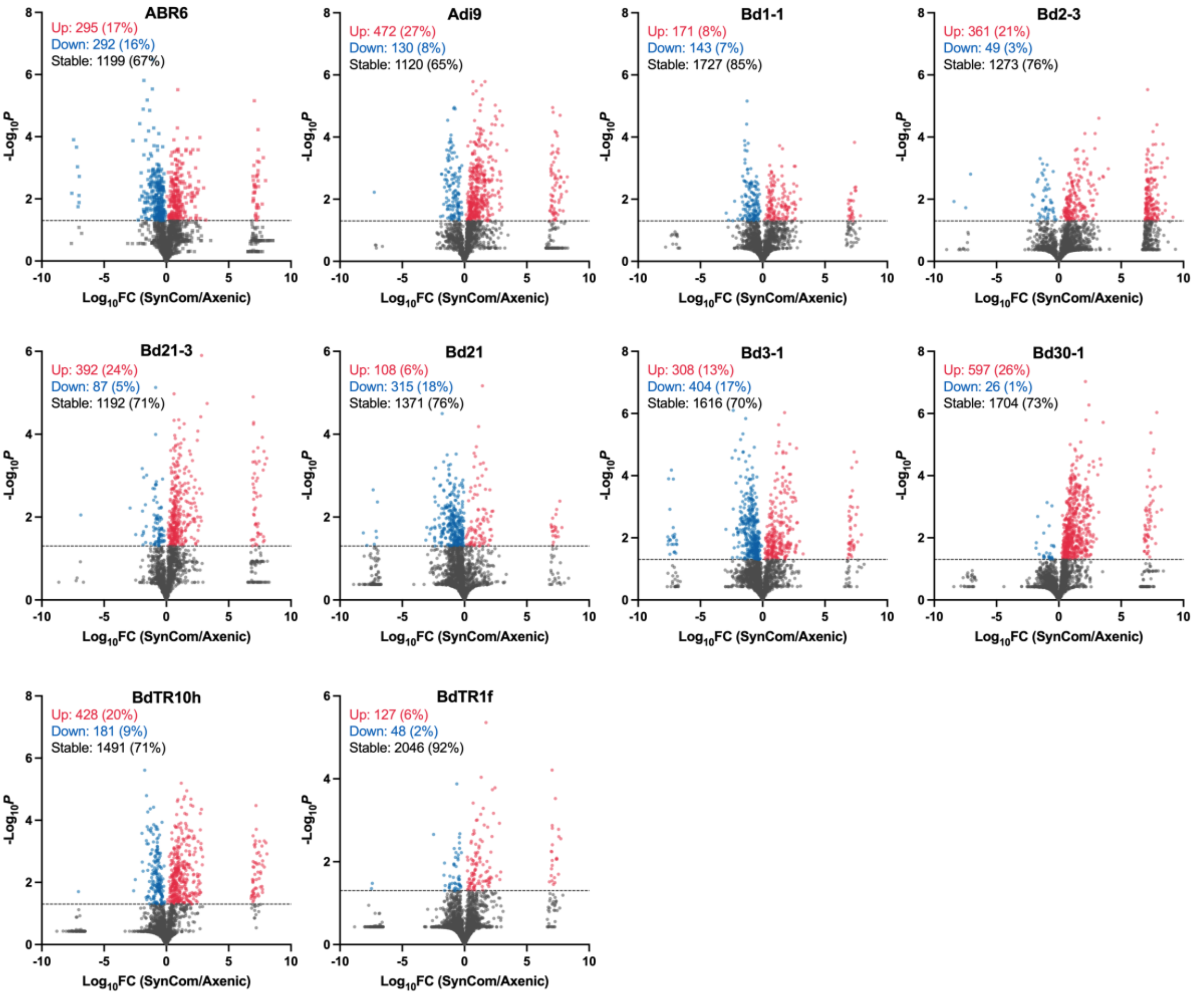

Supplementary Fig. 7. Exudate feature changes of individual *B. distachyon* lines in response to the SynCom. Volcano plots showing the Log_{10} fold changes ($Log_{10}FC$) of features detected in root exudates of individual lines treated with the SynCom versus the axenic control (x-axis) and $-Log_{10}P$ values derived from student *t*-tests (y-axis). Statistically up-regulated features are labeled in red, and down-regulated features are labeled in blue ($P < 0.05$).

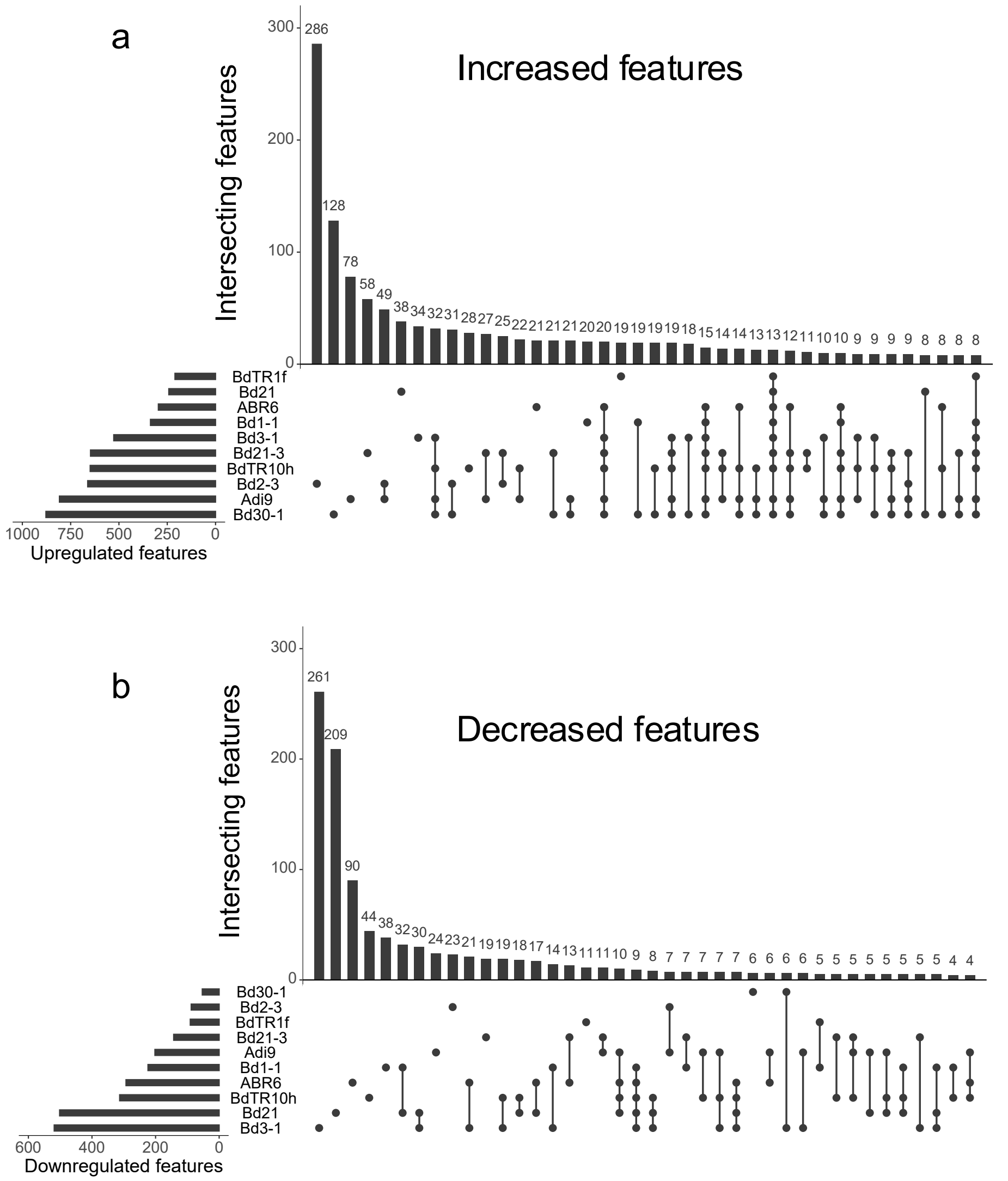

Supplementary Fig. 8. The number of significantly changed features in root exudates of ten *B. distachyon* lines. The UpSet plots show the numbers of shared features that are significantly increased (**a**) and decreased (**b**) in root exudates of ten *B. distachyon* lines in response to the SynCom.

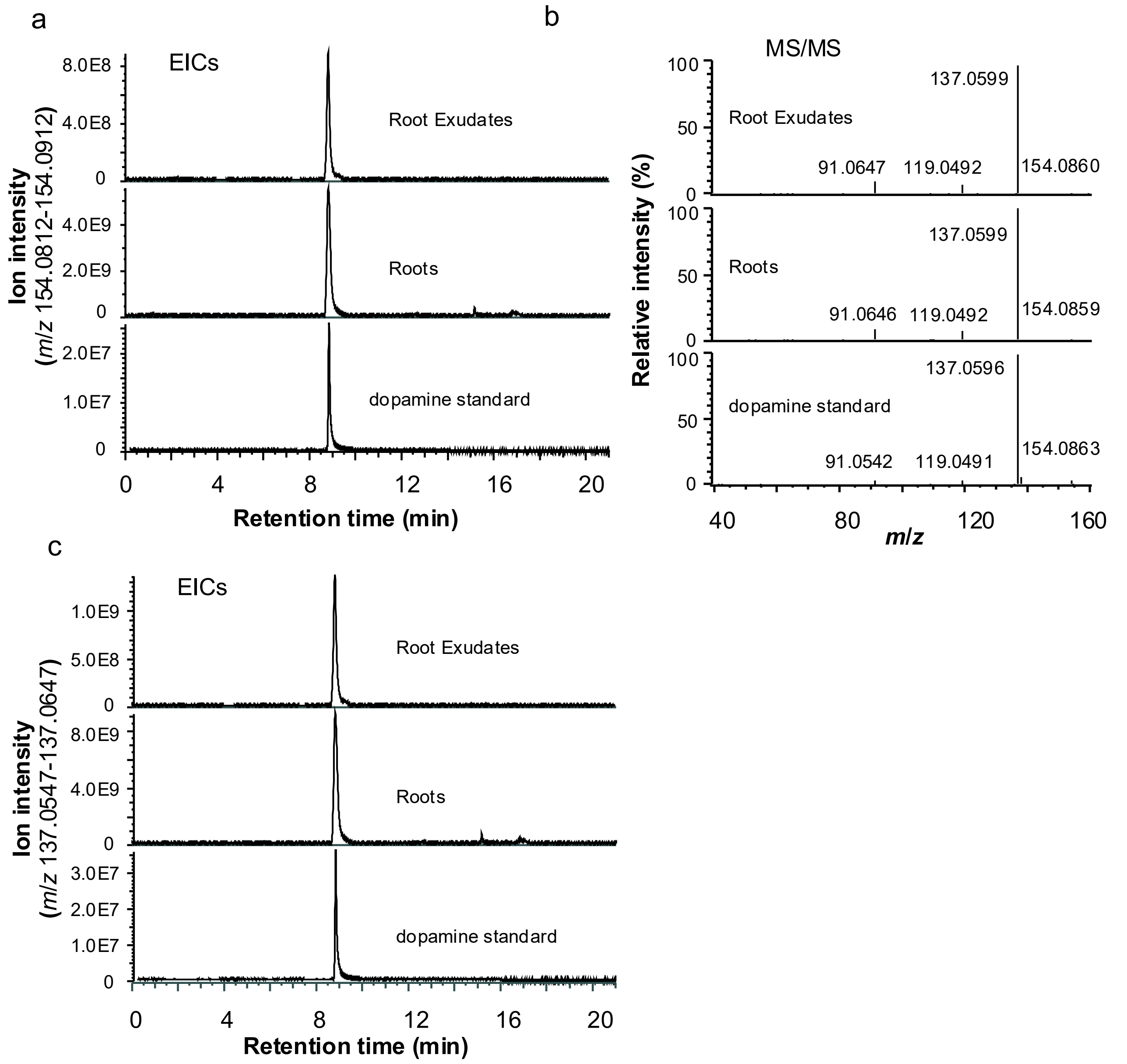

Supplementary Fig. 9. Identification of dopamine in *B. distachyon* using LC-MS/MS. a EICs of dopamine (precursor ion: *m/z* 154.0812-154.0912, $[M + H]^+$) in *B. distachyon* root exudates and roots, aligned with a dopamine standard. **b** MS/MS spectra of dopamine detected in *B. distachyon* root exudates and roots, compared to the dopamine standard. **c** EICs of the dopamine fragment ion (*m/z* 137.0547-137.0647, $[M + H - NH_3]^+$) in roots and root exudates, aligned with the dopamine standard. *B. distachyon* Bd21-3 plants were grown hydroponically, and roots along with the growth medium (containing root exudates) were harvested from 4-week-old plants for LC-MS/MS analysis.

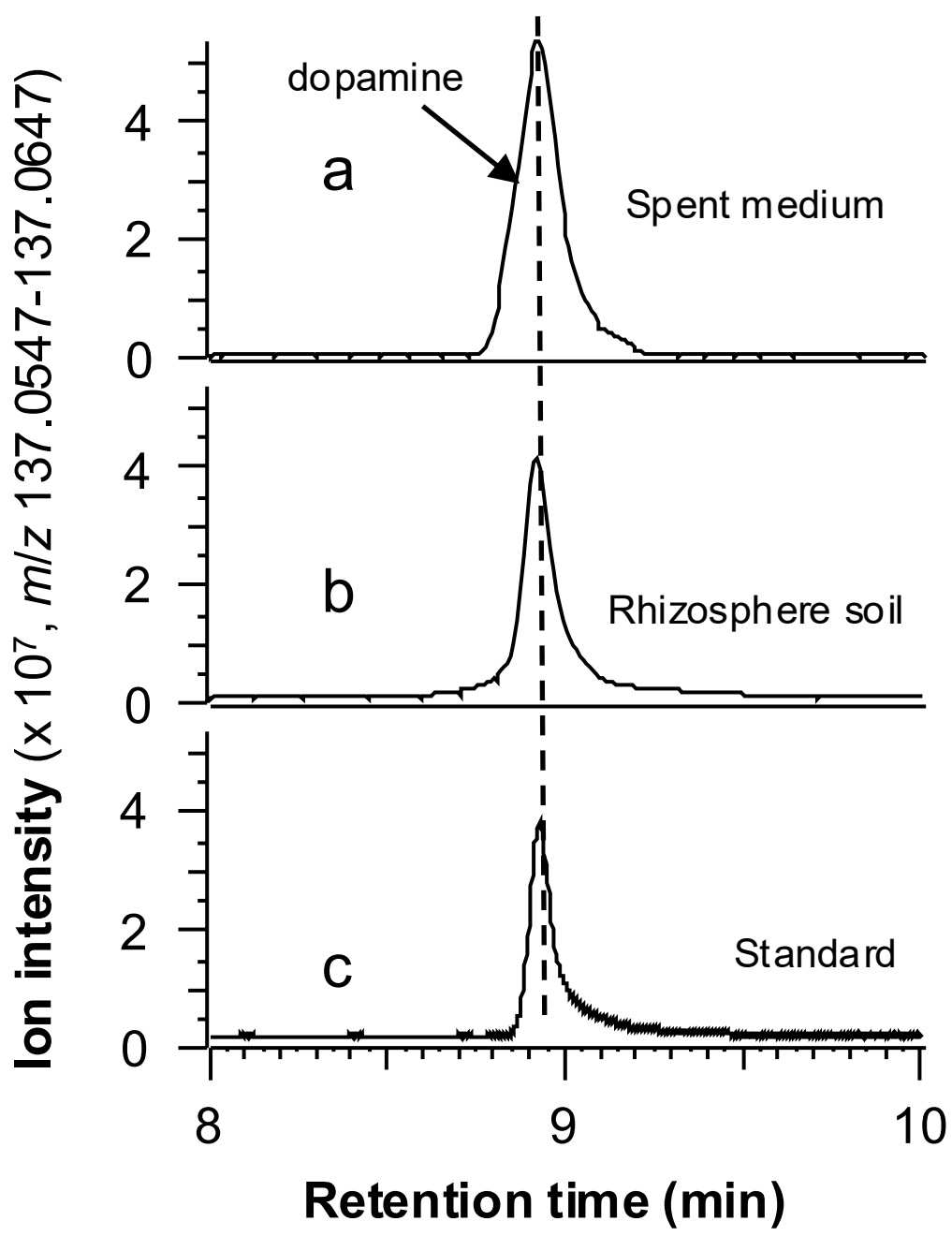

Supplementary Fig. 10. Identification of dopamine in *B. distachyon* root exudates and rhizosphere soil. Bd21-3 plants were grown in a hydroponic system and in soil. The growth medium and the rhizosphere soil were collected from 4-week-old plants for LC-MS/MS analysis. The figure shows LC-MS/MS EIC chromatograms of dopamine $[M + H - NH_3]^+$ ion with *m*/*z* 137.0547-137.0647, in the growth medium (**a**) and the rhizosphere soil (**b**) as compared to the standard (**c**).

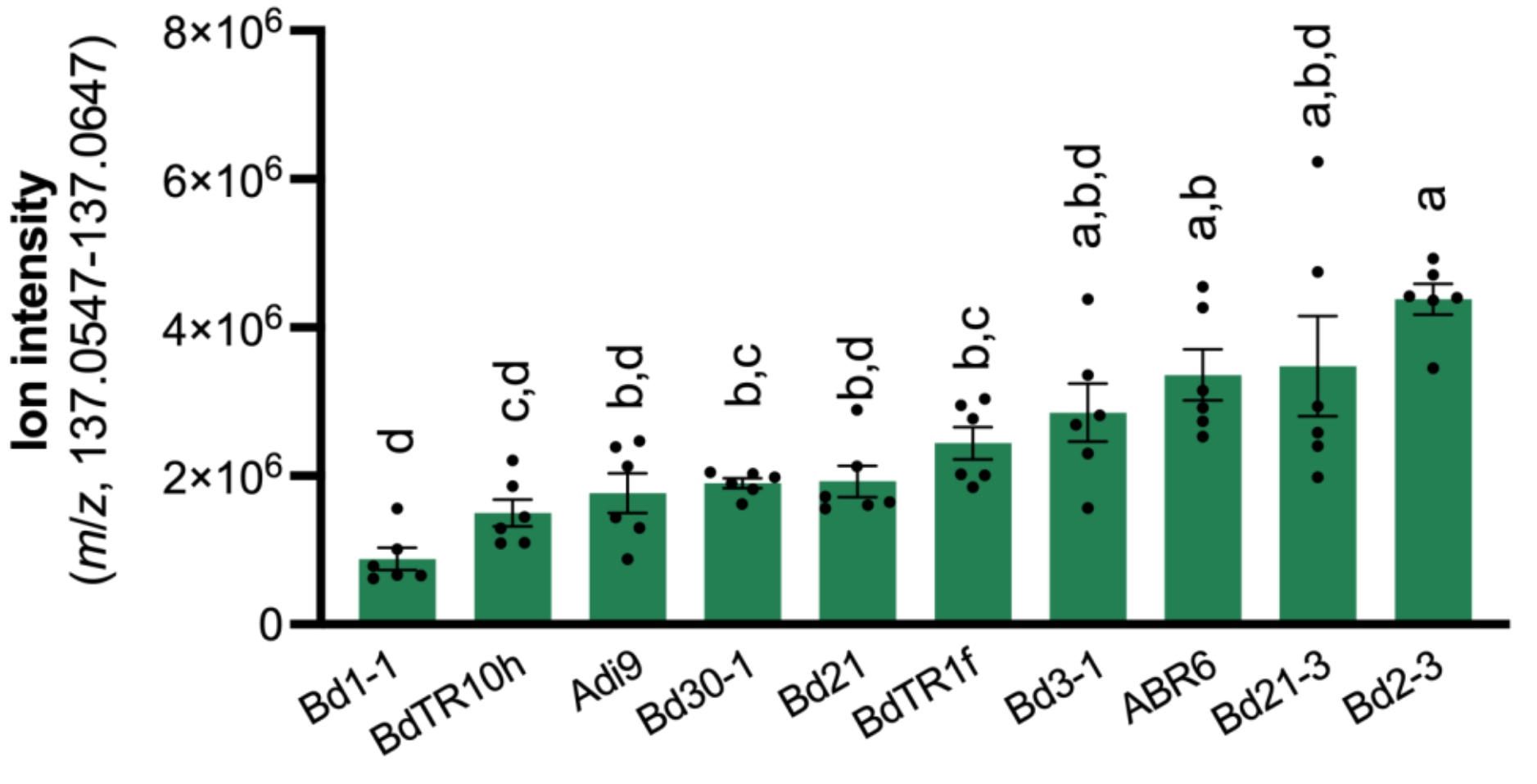

Supplementary Fig. 11. Dopamine levels in root exudates vary across ten *B. distachyon* lines under axenic conditions. Ten *B. distachyon* lines were grown in a hydroponic system in the absence of the 16-member bacterial SynCom. Plant spent media were collected at week 4 for LC-MS/MS analysis. Error bars represent the mean ± SEM of the peak height (*m/z,* 137.0547-137.0647) (n = 6 biologically independent samples). Different letters (a-d) indicate significant differences as determined by two-sided Welch's ANOVA followed by Dunnett's T3 multiple comparison test (P < 0.05). P values were provided in the source data.

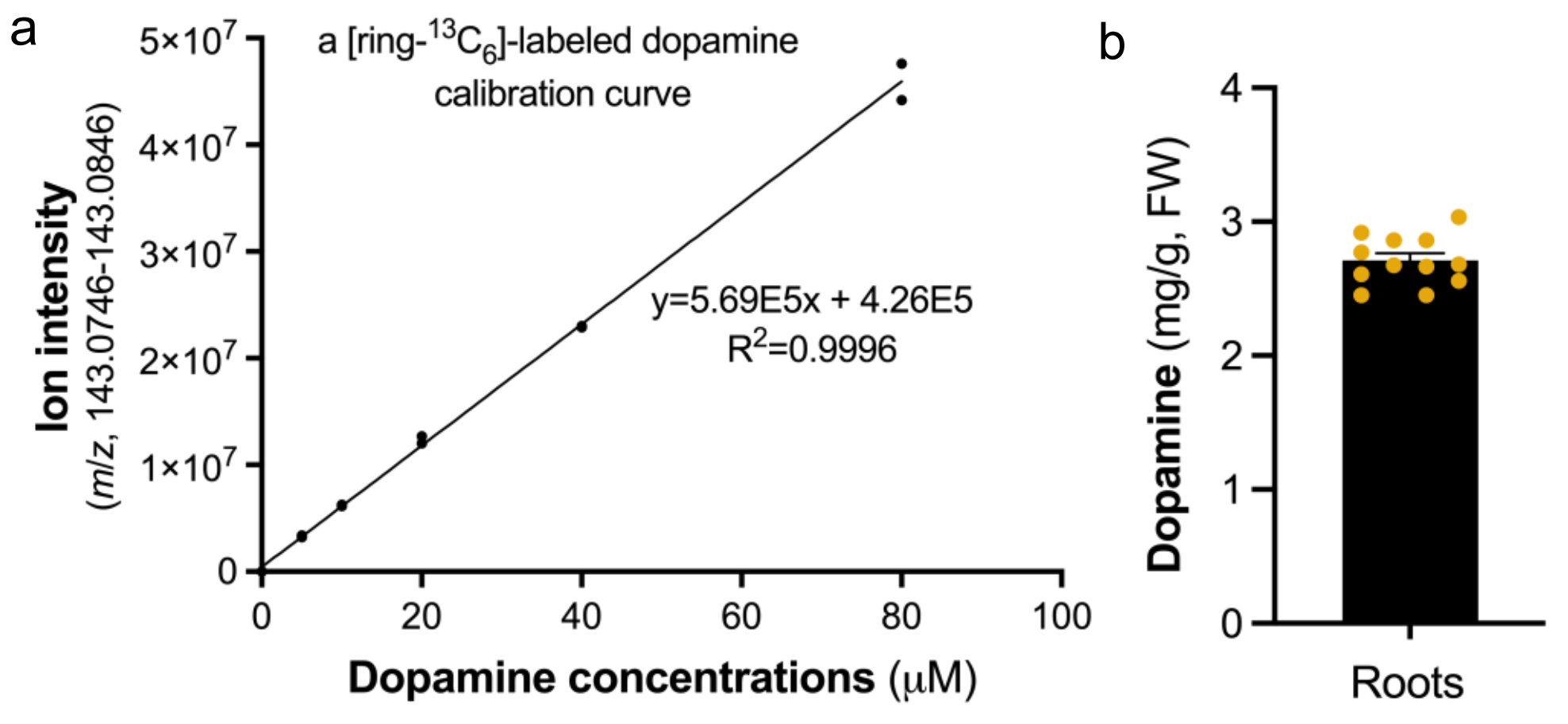

Supplementary Fig. 12. Quantification of dopamine in *B. distachyon* roots. a showing the internal dopamine calibration curve, which was derived based on the ion intensity of a [ring-$^{13}C_6$]-labeled dopamine (*m*/*z,* 143.0746-143.0846, $[M + H - NH_3]^+$) over its concentration (*n* = 2). [Ring-$^{13}C_6$]-labeled dopamine was spiked into the liquid tissue extract as an internal standard. The calibration curve was used to calculate the endogenous ^{12}C-dopamine concentration within the extract and is presented in µM units to allow direct comparison between ^{12}C- and ^{13}C- dopamine values. **b** showing endogenous dopamine levels (*m*/*z* 137.0547-137.0647, $[M + H - NH_3]^+$) in *B. distachyon* roots measured based on the internal calibration curve (**a**) of a [ring-$^{13}C_6$]-labeled dopamine (Cambridge Isotope Laboratories). FW, fresh weight. Error bars represent mean ± SEM (*n* = 12 biologically independent samples).

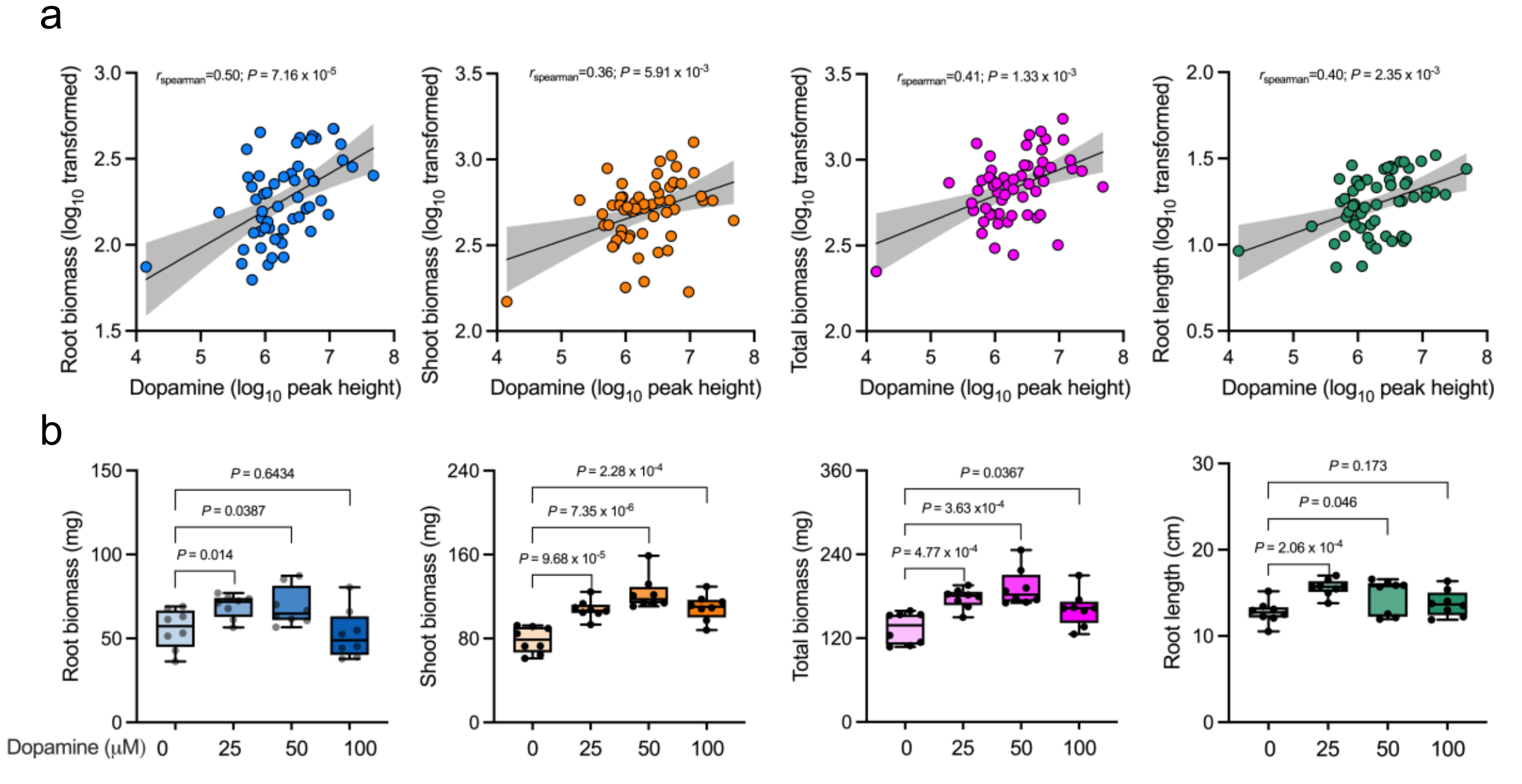

Supplementary Fig.13. Dopamine impacts plant growth in a dose-dependent manner. a Two-sided spearman correlation between exudate dopamine levels and plant growth phenotypes, including root biomass, shoot biomass, total biomass, and root length. Log_{10} transformed data was used for correlation analysis (n = 57). **b** Dopamine could promote *B. distachyon* growth. *B. distachyon* Bd21-3 plants were grown in a hydroponic system supplemented with various concentrations of dopamine. Error bars represent mean ± SEM (n = 8 biologically independent samples). The *P*-value was calculated using a two-sided student's *t*-test.

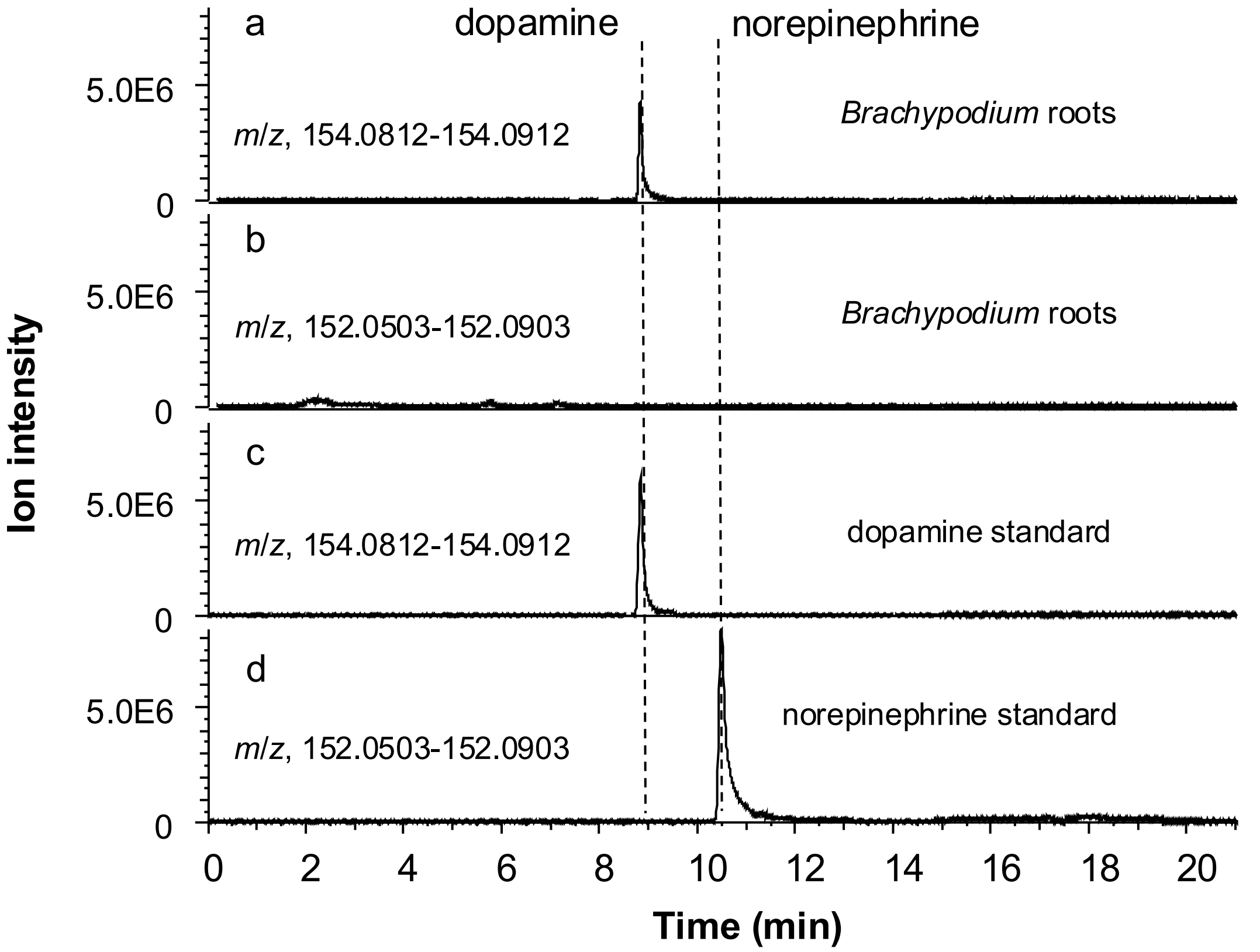

Supplementary Fig. 14. Identification of norepinephrine in *B. distachyon* roots. Bd21-3 plants were grown in a hydroponic system. The 4-week-old roots were used for LC-MS/MS analysis. The figure shows LC-MS/MS EIC chromatograms of dopamine ($[M + H]^+$) (**a** and **c**) and norepinephrine ($[M + H]^+$) (**b** and **d**) in *B. distachyon* roots as well as standards. Chromatograms are representatives of three biological replicates.

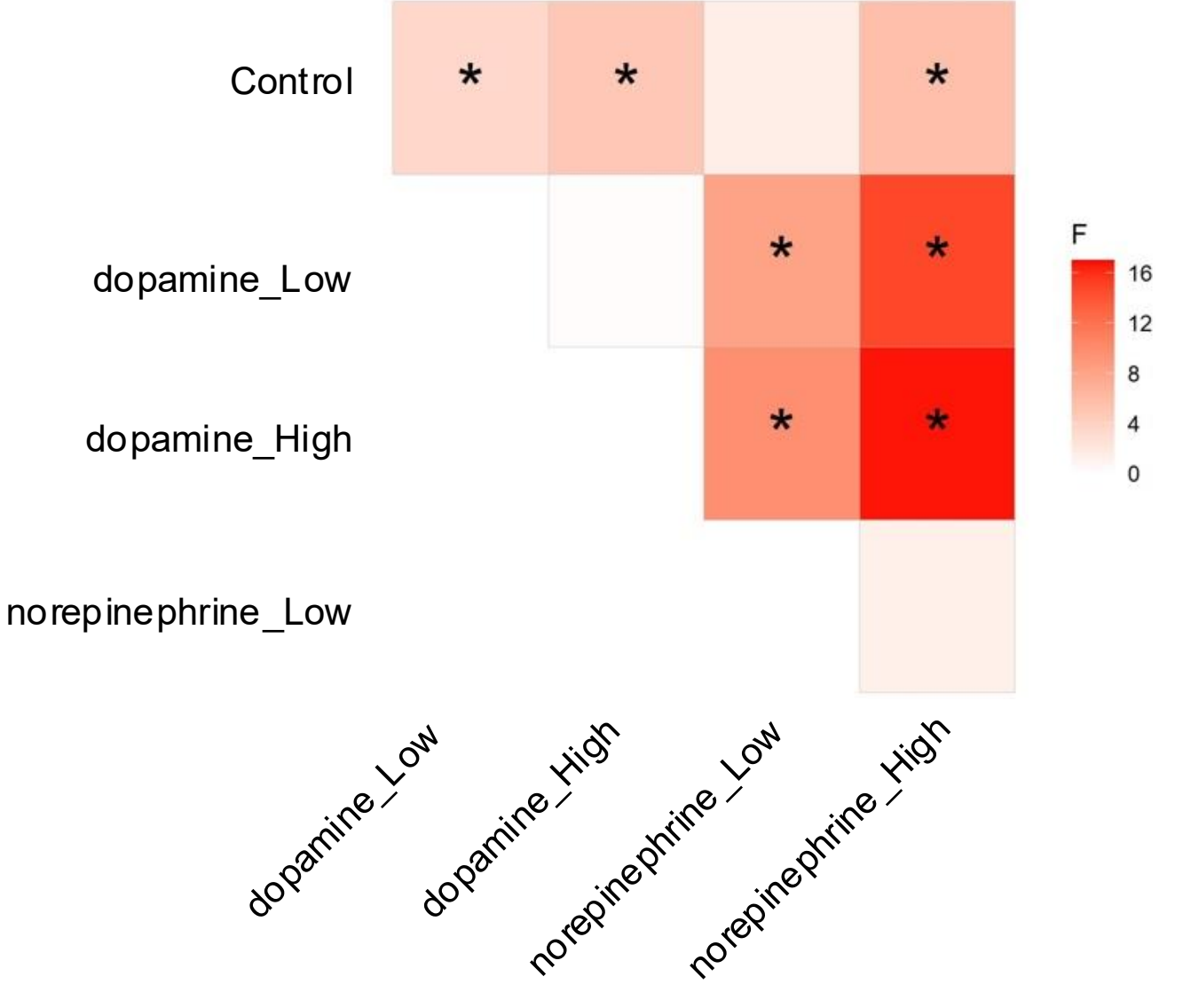

Supplementary Fig. 15. β-diversity of soil microbial communities in response to dopamine and norepinephrine. PERMANOVA was performed on the Bray-Curtis Dissimilarity among soil microbial communities in response to dopamine and norepinephrine treatments. The color of the matrix indicates the F score of the PERMANOVA between two treatments. Asterisks indicate significant β-diversity (*P*-value < 0.05).

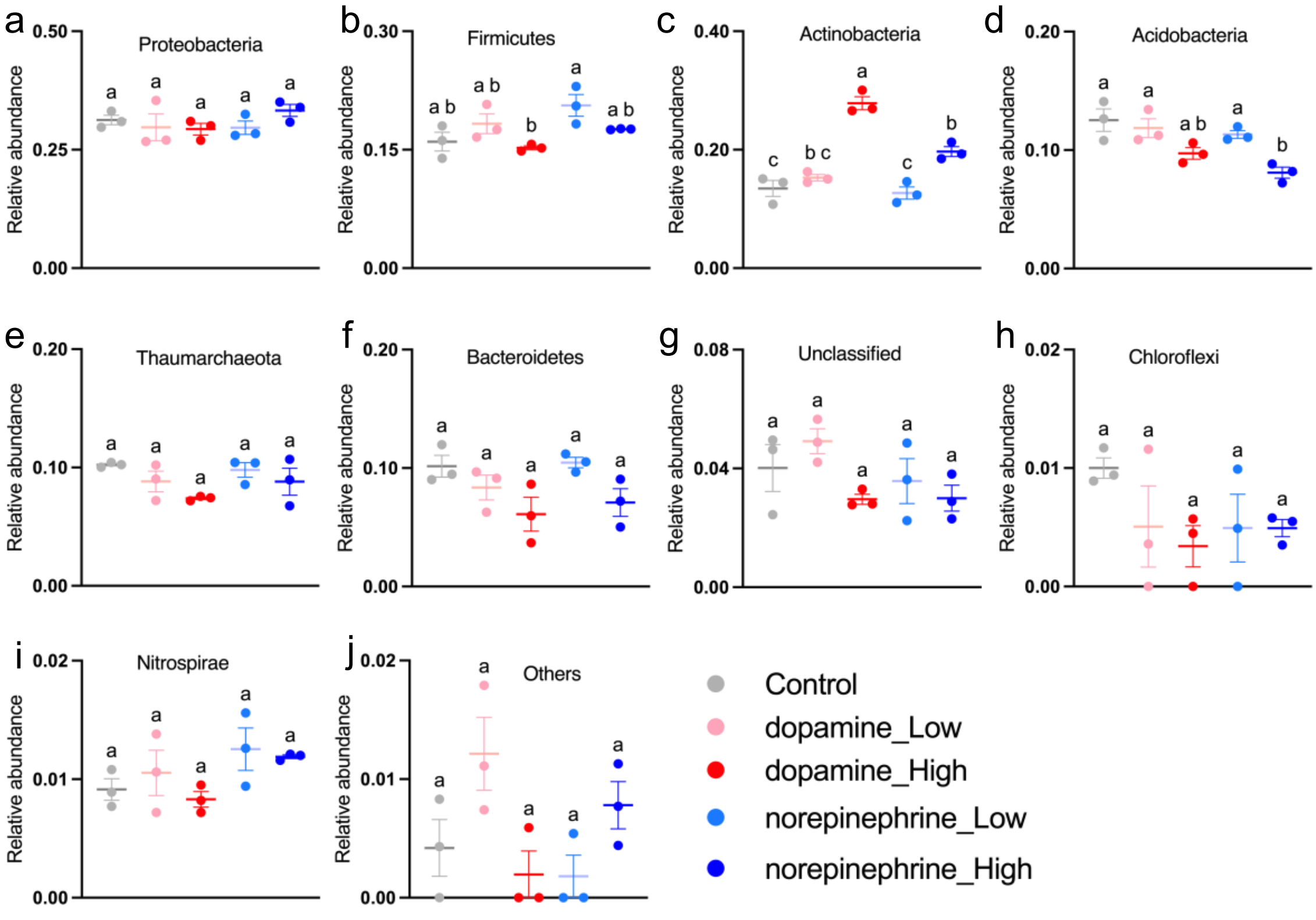

Supplementary Fig. 16. Dopamine enriched *Actinobacteria* in the soil at the phylum level. a - j showing bacterial phylum affected by dopamine and norepinephrine treatments. Error bars represent mean ± SEM (n=3 biologically independent samples). Within plots, different letters represent significant differences (one-way ANOVA followed by Tukey's test corrections for multiple comparisons; $P < 0.05$).

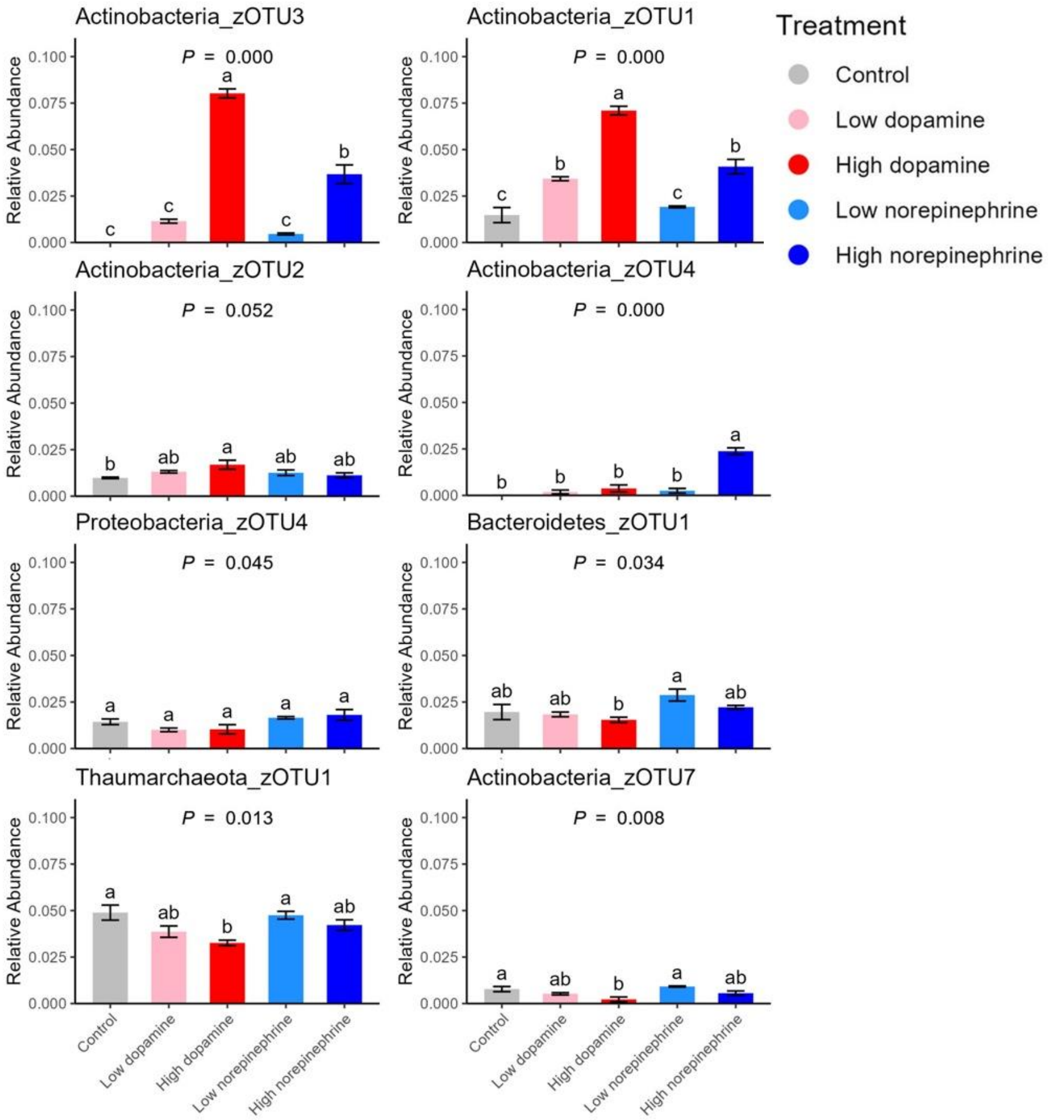

Supplementary Fig. 17. Soil microbial response to dopamine and norepinephrine treatments. The lsmeans of relative abundances of zOTUs with significant (*P*-value < 0.05; except Actinobacteria_zOTU2) ANOVA results by treatment levels. Error bars indicate the standard error of the mean (*n* =3 biologically independent samples), and lsmeans with different lower letters within each zOTU are statistically different (α = 0.05) based on Tukey's post-hoc test.
