# Supplementary material for: Insights into the role of dopamine in rhizosphere microbiome assembly
Source: Nat Commun. 2026 Jul 29;17:9212. doi: 10.1038/s41467-026-76211-1 (PMC13522444; doi:10.1038/s41467-026-76211-1)
Supplement: Supplementary file 4 [file 41467_2026_76211_MOESM2_ESM.pdf]

Description of Additional Supplementary Information

Supplementary Data 1: The soil bacterial isolates used for the construction of the 16-member SynCom in this study.

Supplementary Data 2: The 16S rRNA sequencing data in the rhizhosphere of ten *B. distachyon* lines grown in a hydroponics system.

Supplementary Data 3: Filtered features detected in root exudates of ten *B. distachyon* lines grown in a hydroponics system.

Supplementary Data 4: Dopamine peak height detected in the spent medium of ten *B. distachyon* lines.

Supplementary Data 5: The 16S rRNA sequencing data of soil treated with dopamine and norepinephrine.

Supplementary Data 6: The details of the chromatography and mass spectrometry parameters.

Supplementary Data 7: Annotation evidence for the dopamine identification using Metatlas.
